# Carborane‐Based Analogs of Celecoxib and Flurbiprofen, their COX Inhibition Potential, and COX Selectivity Index

**DOI:** 10.1002/cmdc.202500166

**Published:** 2025-05-04

**Authors:** Lea Ueberham, Jonas Schädlich, Kim Schramke, Sebastian Braun, Christoph Selg, Markus Laube, Peter Lönnecke, Jens Pietzsch, Evamarie Hey‐Hawkins

**Affiliations:** ^1^ Centre for Biotechnology and Biomedicine (BBZ) Faculty of Chemistry and Mineralogy Institute of Bioanalytical Chemistry Universität Leipzig Deutscher Platz 5 04103 Leipzig Germany; ^2^ Department of Radiopharmaceutical and Chemical Biology Institute of Radiopharmaceutical Cancer Research Helmholtz‐Zentrum Dresden‐Rossendorf (HZDR) Bautzner Landstraße 400 01328 Dresden Germany; ^3^ School of Science Faculty of Chemistry and Food Chemistry Technische Universität Dresden Mommsenstraße 4 01062 Dresden Germany; ^4^ Faculty of Chemistry and Chemical Engineering Department of Chemistry Babeş‐Bolyai University Str. Arany Janos Nr. 11 RO‐400028 Cluj‐Napoca Romania

**Keywords:** bioisosterism, carboranes, cyclooxygenase, inflammation, nonsteroidal anti‐inflammatory drug

## Abstract

The cylcooxygenase isoforms COX‐1 and COX‐2 are involved in the production of prostaglandins in physiological and pathological processes. The overexpression of COX‐2 under inflammatory conditions, its role in cancer and neurodegenerative diseases necessitates the need to develop and improve nonsteroidal anti‐inflammatory drugs. These mainly unselective COX inhibitors, e.g. aspirin, are used to reduce the symptoms of inflammation. To reduce unwanted side effects connected with unselective inhibition, the development of novel COX‐2 selective inhibitors is a major goal. Herein, the synthesis, characterization and *in vitro* biological evaluation of eight flurbiprofen‐ and celecoxib‐based carborane analogs are described. Carboranes as hydrophobic surrogates are suitable substituents that can contribute to a selectivity increase toward COX‐2 due to size exclusion. The inhibitory efficacy for COX‐1 and COX‐2 of the four *ortho*‐ and four *nido*‐carborane derivatives has been tested. The *nido* compounds are much more potent than their *closo*‐carborane analogs. The celecoxib‐based *nido*‐carborane compound **10** shows an IC_50_(COX‐2) value in the sub‐μM range and slight selectivity for COX‐2. This is in contrast to its *ortho*‐carborane counterpart **9**, which shows an inhibition preference for COX‐1. While none of these carborane derivatives outperforms their organic analogs, the flurbiprofen‐based *nido*‐carborane derivatives **14a** and **14b** surpass the known carborane‐based flurbiprofen analogs.

## Graphical abstract

Eight *nido*‐ and *ortho*‐carborane compounds based on celecoxib and flurbiprofen were synthesized and their COX inhibition was determined. The deboronation of the di‐substituted celecoxib derivative results in a switch from COX‐1 inhibitory preference to COX‐2 inhibition. The *nido*‐carborane‐flurbiprofen derivative with a methylene spacer outperformed the derivative with ethylene spacer and literature‐known flurbiprofen‐carborane conjugates. All *nido*‐carborane analogs exceed the parent *ortho*‐carborane derivatives.

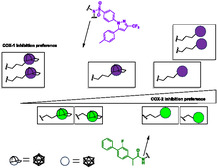

## 1 Introduction

Inflammatory processes are a (patho)physiological reaction of the human body as part of the innate immune response to negative triggers or invaders and also occur in a variety of diseases such as cancer, neurodegenerative diseases, and disorders of the immune system.^[^ ^1^, ^2^ ^]^

The cyclooxygenases COX‐1 and COX‐2 are fundamentally involved in these inflammatory processes.^[^ ^2^, ^3^ ^]^ The two isoenzymes share around 60% sequence identity and are both homodimers.^[^ ^4^ ^]^ Each monomer is divided into three domains: the epidermal growth factor, the membrane‐binding, and the catalytic domain. The catalytic domain is further structured into cyclooxygenase and peroxidase active sites.^[^ ^2^ ^]^ Cyclooxygenase‐2 has a larger active site than cyclooxygenase‐1 (≈25%).^[^ ^2^, ^4^ ^]^ The additional secondary side pocket present in COX‐2 enables a size exclusivity for larger molecules,^[^ ^2^ ^]^ thus enabling selectivity between these two isoforms which share a high similarity.^[^ ^5^ ^]^ Other differences between COX‐1 and COX‐2 include their expression, regulation, and tissue distribution.^[^ ^2^, ^6^ ^]^

Whereas COX‐1 is constitutively expressed in most tissues,^[^ ^2^ ^]^ COX‐2 is expressed constitutively in the brain, kidney, ovary, uterus and gut,^[^ ^2^, ^6^ ^]^ but also referred to as inducible enzyme, since it is expressed under inflammatory conditions.^[^ ^7^ ^]^

Both enzymes are involved in the production of prostaglandins, which take part in physiological processes, but are also key players in pathological conditions and are therefore inflammation mediators.^[^ ^8^ ^]^ For treatment, non‐steroidal anti‐inflammatory drugs (NSAIDs) have been developed. They are widely applied, with aspirin, used for over 120 years, being the most prominent representative.^[^ ^9^, ^10^ ^]^ Other commonly used NSAIDs are indomethacin, diclofenac, ibuprofen, or flurbiprofen, to name but a few.^[^ ^11^ ^]^ They act as inhibitors of COX‐1 and COX‐2 and thereby limit the production of the prostaglandins under pathological conditions.^[^ ^9^, ^12^ ^]^ Due to their unselective blocking of both isoforms, the application of NSAIDs may cause a series of adverse side effects, such as a higher risk of gastrointestinal (GI) problems.^[^ ^9^, ^11^, ^12^, ^13^, ^14^ ^]^ To maintain the positive effects of NSAIDs but reduce the unwanted ones, COX‐2 selective inhibitors (COXIBs) have been developed.^[^ ^5^ ^]^ Important members of this class are celecoxib, etoricoxib, valdecoxib, and rofecoxib; however, the last two have already been withdrawn from the market.^[^ ^9^ ^]^ Even though the application of selective COX‐2 inhibitors lowers the risk of GI trouble, new unwanted side effects have been noticed, most notably an increased risk for cardiovascular events.^[^ ^9^ ^]^ The design of compounds with a better safety profile, but still high affinity and selectivity for COX‐2, is therefore a very important research area.

**Scheme 1:**
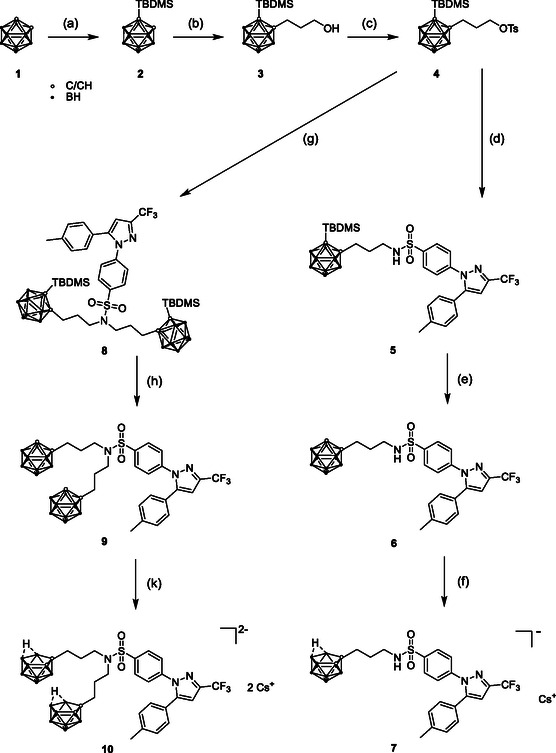
Synthesis of target compounds **6**, **7**, **9**, and **10**. Reagents and conditions: a) (i) *n*‐butyllithium (*n*‐BuLi), 0 °C → rt, 2 h, (ii) *tert*‐butyldimethylsilyl chloride (TBDMSCl), toluene/Et_2_O (2:1, *v*/*v*); b) (i) *n*‐BuLi, 0 °C → rt, 2 h, (ii) oxetane, Et_2_O, 0 °C → rt, 24 h; c) (i) *p*‐toluenesulfonyl chloride, 0 °C, (ii) pyridine, CH_2_Cl_2_, 0 °C → rt, 4 h; d) and g) celecoxib, Cs_2_CO_3_, *N*,*N*‐dimethylformamide (DMF), 0 °C → rt, 24 h; for e) and h) tetrabutylammonium fluoride (TBAF·H_2_O, tetrahydrofuran (THF), −78 °C → 0 °C; f) and k) CsF, EtOH/H_2_O (1:1, *v*/*v*), microwave 150 °C, 10 min.

In the past decades, many carborane‐based derivatives for applications in medicinal chemistry have been published,^[^ ^15^, ^16^, ^17^, ^18^, ^19^, ^20^ ^]^ including work from our group.^[^ ^21^, ^22^, ^23^ ^]^ Carboranes or *closo*‐dicarbadodecaboranes(12) are icosahedral clusters with two carbon‐hydrogen and ten boron‐hydrogen units. Depending on the relative position of the two carbon vertices, a division in *ortho* (1,2; **Scheme** **1**; compound **1**), *meta* (1,7), and *para* (1,12) isomer is possible, in analogy to substituents in benzene.^[^ ^20^ ^]^ A deboronation to *nido*‐carboranes is possible, with the *ortho* isomer being most prone to undergo this reaction.^[^ ^24^ ^]^ The *nido*‐carborane derivatives often offer improved solubility in aqueous media.^[^ ^25^ ^]^ Whereas the classification of isomers of carboranes and benzene is similar, volume‐wise (van der Waals volumes) carboranes are better compared with adamantane. Regardless of that, carboranes are often referred to as “three‐dimensional analogs of benzene” and share the delocalization of electrons over the whole structure with the difference of exhibiting σ delocalization instead of π delocalization.^[^ ^20^, ^24^ ^]^

The three‐dimensional molecules can be substituted in an orthogonal approach, since the BH and CH units differ in their electronic properties and thus facilitate regioselective substitutions,^[^ ^24^ ^]^ also including radiolabeling.^[^ ^22^, ^26^ ^]^ Another benefit meeting the requirements for compounds to be used in a physiological context is that a higher metabolic stability of carboranes can be expected *in vivo* compared to purely organic compounds.^[^ ^16^, ^17^, ^18^, ^19^, ^20^, ^21^, ^22^, ^27^ ^]^ Furthermore, the high hydrophobicity is favorable for crossing the blood–brain barrier, and noncovalent interactions of carboranes with targets *in vivo* make them ideal for medicinal purposes.^[^ ^21^ ^]^

**Figure 1:**
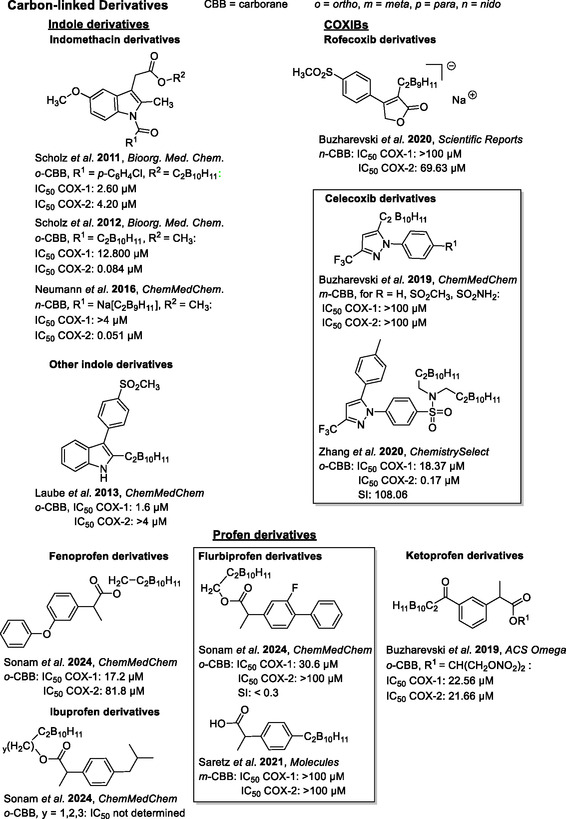
Selected examples of NSAID‐based carborane analogs (IC_50_ values for COX‐1 and COX‐2 inhibition are given, if reported). All derivatives shown here are carbon‐substituted carboranes.^[^ ^34^, ^35^, ^36^, ^37^, ^38^, ^39^, ^40^, ^41^, ^42^, ^43^, ^44^ ^]^

**Figure 2:**
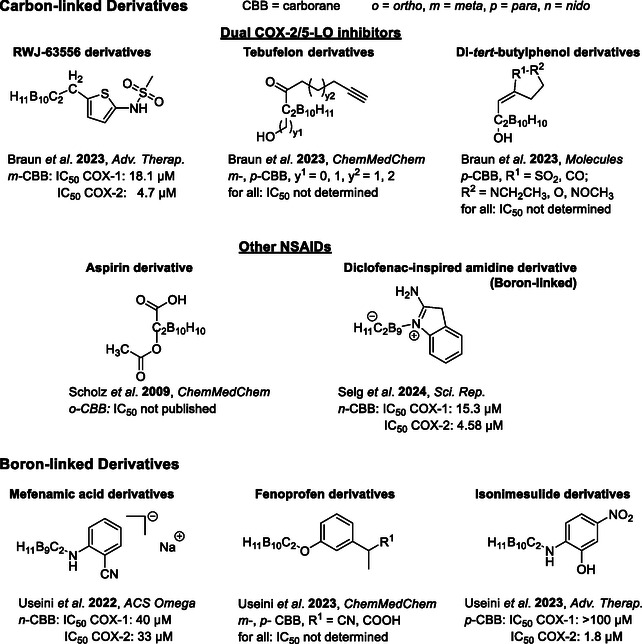
Selected examples of NSAID‐related carborane derivatives. Top: carbon‐substituted carboranes; bottom: boron‐substituted carboranes.^[^ ^28^, ^29^, ^30^, ^31^, ^32^, ^41^, ^44^, ^45^ ^]^

Carboranes are often used as substitutes of cyclopropyl, adamantyl or phenyl groups, e.g., as modifiers for NSAIDs (**Figure** **1** and **2**).^[^ ^28^, ^29^, ^30^, ^31^, ^32^, ^33^, ^34^, ^35^, ^36^, ^37^, ^38^, ^39^, ^40^, ^41^, ^42^, ^43^, ^44^, ^45^ ^]^ Among others, they could contribute to an increased selectivity towards COX‐2, because of an increase of size of the inhibitors.

As depicted in Figures 1 and 2, a broad range of various scaffolds has been modified with carboranes, most of them linked via one of the carborane carbon atoms and only a few via boron.^[^ ^28^, ^29^, ^30^ ^]^ The inhibition activity (IC_50_ values) of carborane‐based COX‐inhibitors varies in a wide range, with the *nido*‐carborane indomethacin‐ester reported by Neumann et al.^[^ ^36^ ^]^ showing a remarkable IC_50_(COX‐2) of 0.051 μM, not only outperforming the organic reference, but also being the most promising carborane‐based NSAID with COX‐2 selectivity shown to date. Compounds based on celecoxib and flurbiprofen only showed insufficient IC_50_ values in the micromolar range with only low selectivity for the COX isoforms. An exception is the celecoxib‐based compound reported by Zhang et al. (Figure 1),^[^ ^44^ ^]^ which is substituted with two carborane moieties and shows a good COX‐2 inhibitory efficacy (IC_50_(COX‐2) = 0.17 μM) and selectivity (selectivity index (SI) = 108.06). This derivative has one CH_2_ unit as spacer between the COX‐2‐selective drug and the carborane substituents. Further structure–activity relationship regarding spacer length, or influence of mono‐ and di‐substitution with carboranes was not presented by the authors.

**Figure 3:**
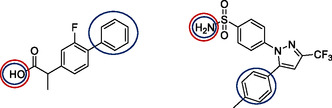
Modification sites of flurbiprofen and celecoxib with carborane derivatives; past work: dark blue circles,^[^ ^38^, ^39^, ^40^, ^41^, ^42^, ^43^, ^44^ ^]^ this work: red circles.

Based on the earlier described inhibitors, we identified carboranyl‐esters of flurbiprofen and di‐carboranyl‐substituted celecoxib derivatives with a short spacer as next promising leads for generation of carborane‐based COX‐2 inhibitors. We aimed to develop carboranyl‐amides of flurbiprofen as well as explore the structure−activity relationship (SAR) of *N*‐substituted carboranyl‐celecoxib inhibitors with prolonged spacer. We here report the synthesis, characterization, and the results of COX‐1 and COX‐2 inhibition assays of eight carborane‐based compounds in which the flurbiprofen or celecoxib scaffolds have been modified in the positions shown in **Figure** **3** with *ortho*‐ and *nido*‐carborane clusters to increase the size and enhance COX‐2 selectivity.

## 2 Results and Discussion

### 2.1 Synthesis

The synthesis of the carborane‐based mono‐substituted celecoxib derivative **6** was achieved in a five‐step reaction (Scheme 1). In the first step, one carbon atom of the *ortho*‐carborane was protected with *tert*‐butyldimethylsilyl chloride (TBDMSCl) according to Ahrens et al.^[^ ^46^, ^47^ ^]^ The second carborane‐carbon atom was lithiated, and the lithium salt was reacted with oxetane in a ring‐opening reaction to give carboranyl‐propanol as described in the literature,^[^ ^46^, ^48^ ^]^ followed by tosylation of the hydroxy group.^[^ ^49^, ^50^ ^]^ Celecoxib was reacted with **4** in an S_N_2 reaction.^[^ ^51^ ^]^ The resulting compound **5** was deprotected in the last step with tetrabutylammonium fluoride (TBAF)^[^ ^47^ ^]^ to give the target molecule **6**. As side product of the reaction with celecoxib, di‐substituted compound **8** was isolated. Treatment similar to **5** gave deprotected **9**.

The *closo*‐carborane derivatives **6** and **9** could be transformed into the corresponding *nido*‐carborane analogs **7** and **10** via deboronation with CsF in aqueous EtOH in a microwave reaction.^[^ ^52^ ^]^ Compounds **6** and **9** were purified by column chromatography. The workup for the *nido*‐carborane derivatives **7** and **10** required multiple repetitions of column chromatography and subsequent washout from the stationary phase. The four celecoxib‐based compounds **6**, **7**, **9**, and **10** were characterized by 1D and 2D NMR spectroscopy and high‐resolution ESI mass spectrometry. For **9**, single crystals could be obtained from CDCl_3_ and the molecular structure could be elucidated by X‐ray crystallography (Figure S84, Supporting Information).

**Scheme 2:**
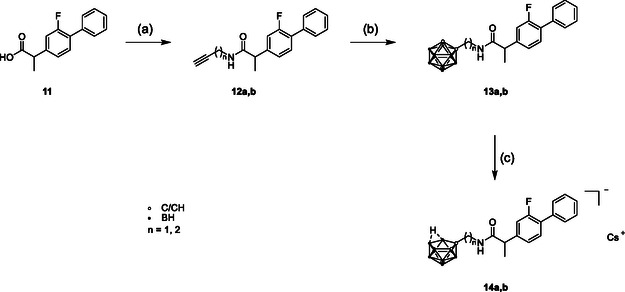
Synthesis of target compounds **13a**, **13b**, **14a**, and **14b**. Reagents and conditions: a) (i) SOCl_2_, DMF cat., CH_2_Cl_2_, 0 °C → rt, 21 h, (ii) respective amine, triethylamine, THF, 0 °C → rt, 21 h; b) B_10_H_14_, *N*,*N*‐dimethylaniline, chlorobenzene, microwave, 120–150 °C, 30 min; and c) CsF, EtOH/H_2_O (1:1, *v*/*v*), microwave, 150 °C, 10 min.

The racemic flurbiprofen derivatives **13a** and **13b** were synthesized according to **Scheme** **2**. Flurbiprofen was chlorinated and reacted with propargylamine or 3‐butyn‐1‐amine to give the respective carboxamides **12a** and **12b**.^[^ ^53^, ^54^, ^55^ ^]^ The following reaction with decaborane in a microwave reactor with *N*,*N*‐dimethylaniline as base and chlorobenzene as solvent gave the *ortho*‐carborane derivatives **13a** and **13b**.^[^ ^56^ ^]^

Both compounds were deboronated according to a literature procedure^[^ ^52^ ^]^ to give **14a** and **14b** as isomeric mixtures (C‐chiral and planar chiral). Compounds **13a,b** and **14a,b** were purified by column chromatography. However, purification of compounds **14a** and **14b** was challenging, required multiple columns and final purification by semipreparative column chromatography on an RP‐HPLC. Compounds **13a,b** and **14a,b** were characterized by 1D and 2D NMR spectroscopy and HR‐ESI mass spectrometry. The purity (≥ 95%) of all eight target compounds was confirmed by HPLC‐UV‐MS or analytical HPLC (in that case: UPLC‐MS for mass confirmation used prior to that separately) on an RP column (see Supporting Information for details, “3 HPLC Purity Determination of Compounds **6**, **7**, **9**, **10**, **13a**, **13b**, **14a**, **14b**”).

The stability of compounds **6**, **7**, **9**, **10**, **13a,b**, and **14a,b** in aqueous solution was studied by HPLC‐UV‐MS or analytical HPLC measurements as well (see Supporting Information for details, “4 HPLC Stability Determination of Compounds **6**, **7**, **9**, **10**, **13a**, **13b**, **14a**, **14b**”). All compounds except **13a** were stable for at least one  day (Supporting Information, section 4 ).

### 2.2 Biological Evaluation

Compounds **6**, **7**, **9**, **10**, **13a,b**, and **14a,b** were evaluated in an *in vitro* COX assay (using the COX Fluorescent Inhibitor Screening Assay Kit from Cayman Chemical Company) regarding their inhibition efficacy towards ovine COX‐1 and recombinant human COX‐2 at a concentration of 100 μM. For promising compounds, the IC_50_ values and the selectivity index (IC_50_(COX‐1)/IC_50_(COX‐2)) were determined with celecoxib and SC‐560 as references (**Table** **1**).

**Table 1:** COX inhibition studies with compounds **6**, **7**, **9**, **10**, **13a,b**, and **14a,b**.

Compound (*ortho‐closo*)	%‐inhibition at 100 [μM] (*n* = 2)		IC_50_ [μM] (95% CI)		SIa)	Compound (*ortho‐nido*)	%‐inhibition at 100 [μM] (*n *= 2)		IC_50_ [μM] (95% CI)		SIa)
	**COX‐1**	**COX‐2**	**COX‐1**	**COX‐2**			**COX‐1**	**COX‐2**	**COX‐1**	**COX‐2**	
**6**	9b)	54b)	n.d	120	/	**7**	94	100	11.2	8.75	1.28
**9**	38	n.i.	>320	n.d.	/	**10**	103	103	3.49	0.34	10.3
**13a**	24	44	n.d	n.d	/	**14a**	94	91	11.8	6.17	1.9
**13b**	17	27	n.d	n.d	/	**14b**	72	80	34.8	12.7	2.7
**IC**_**50**_ **[μM] as mean ± SD**											
	**COX‐1 (** ***n*** ** = 10)**		**COX‐2 (** ***n*** ** = 10)**				**COX‐1 (** ***n*** ** = 10)**		**COX‐2 (** ***n*** ** = 10)**		
**SC‐560**	0.009 ± 0.002		n.d.			**Celecoxib**	n.d.		0.102 ± 0.35		

n.d. = not determined, n.i. = no inhibition (%‐inhibition < 5 %), SI = selectivity index;

a) SI = IC_50_(COX‐1)/IC_50_(COX‐2);

b) Inhibition at 98 μM instead of 100 μM.

For the *ortho*‐carborane derivatives **6**, **9**, **13a**, and **13b**, inhibition at 100 μM varied, but did not exceed 55% for COX‐1 or COX‐2. For the celecoxib analogs **6** and **9**, opposite inhibition preferences were observed. The mono‐substituted analog **6** showed a very low inhibition at 100 μM for COX‐1, but moderate inhibition for COX‐2, with an IC_50_ value of 120 μM. The di‐substituted analog **9** showed no inhibition for COX‐2, but low inhibition for COX‐1, with an IC_50_ value > 320 μM. While these celecoxib analogs showed far lower inhibitory efficacy compared to celecoxib, the inversion of selectivity for the di‐substituted analog **9** toward COX‐1 is an interesting observation. This switch is obviously related to the additional substitution at nitrogen with a propyl‐carborane moiety. In contrast to our inactive compound **9**, the celecoxib derivative reported by Zhang et al., which is also a carborane‐based di‐substituted analog, showed a high COX‐2 inhibitory efficacy and selectivity (Figure 1).^[^ ^44^ ^]^ While this compound has only one CH_2_ group between the carborane moiety and the celecoxib scaffold, compound **9** has three CH_2_ units as linker, indicating a high influence of the chain length on COX‐2 inhibitory efficacy. In this case, a longer chain results in reduced COX inhibitory efficacy and a switch from COX‐2 to COX‐1 inhibition.

**Figure 4:**
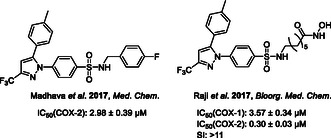
Celecoxib‐based organic analogs with different chain lengths.^[^ ^57^, ^58^ ^]^

Madhava et al. reported *N*‐substituted celecoxib derivatives, in which the derivatives with one CH_2_ spacer retained COX‐2 inhibition (**Figure** **4**).^[^ ^57^ ^]^ Raji and co‐workers published *N*‐substituted celecoxib derivatives with a different organic substituent and alkylene spacers with five to seven CH_2_ units. The compound with six CH_2_ units retained COX‐2 selectivity with an IC_50_(COX‐2) value in the sub‐micromolar range (Figure 4).^[^ ^58^ ^]^ Sonam et al. reported that in general shorter alkylene spacers in carborane‐NSAID conjugates resulted in higher COX inhibition.^[^ ^40^ ^]^ Apparently, the COX inhibitory efficacy is not only influenced by the length of the alkylene spacer but also by carboranes as substituent (structure–activity relationship, SAR).

The two *closo*‐carborane‐based flurbiprofen derivatives **13a,b** have shown low inhibition values and/or selectivity values at 100 μM; therefore, no IC_50_ values were determined.

Compared to the *closo*‐carborane derivatives **6**, **9**, and **13a,b**, a massive improvement of efficacy is observed for the corresponding *nido*‐carborane compounds **7**, **10**, and **14a,b** (IC_50_ values between 34.8 μM and 0.34 μM), as had also been reported before in the literature for other derivatives.^[^ ^28^, ^36^, ^45^ ^]^ Interestingly, the selectivity trend for the *nido*‐carborane‐celecoxib derivative **7** is retained, whereas the selectivity switched for the di‐substituted *nido*‐carborane derivative **10** from a COX‐1 to COX‐2 preference. In case of the flurbiprofen derivatives, the compound with the shorter methylene spacer **14a** was a slightly better inhibitor than the analog **14b** with an ethylene spacer. A similar trend was observed by Sonam et al. for carborane‐NSAID conjugates with varying spacer lengths.^[^ ^40^ ^]^ Compounds **14a** and **14b** outperformed the efficacy of the so far published flurbiprofen‐based carborane‐analogues.^[^ ^40^, ^41^ ^]^

## 3 Conclusion

Inflammation as part of the immune response of the human body is a process that requires special attention, as it is often associated with many well‐known diseases such as neurodegenerative diseases and cancer, but also with specific therapeutic approaches and lengthy healing processes. This is especially true when such accompanying inflammatory processes are uncontrolled, excessive, or chronic and can then lead to massive collateral damage in the affected tissues and organs.^[^ ^59^ ^]^ The discovery of the involvement of COX‐1 and COX‐2 enzymes in processes with inflammation led to the development of unselective NSAIDs and selective COXIBs as COX inhibitors. To minimize unwanted side effects of the therapeutics, which are mainly due to unselective inhibition of both enzymes instead of only COX‐2, the development of suitable drugs is ongoing. Here, we presented the synthesis of four *ortho*‐ and four *nido*‐carborane derivatives with a flurbiprofen and celecoxib scaffold. While the carborane derivatives reported here exhibited lower COX inhibition compared to their organic counterparts, the *nido*‐carborane‐flurbiprofen derivatives showed the highest activity compared to previously published carborane‐based analogs of known COX inhibitors.^[^ ^40^, ^41^ ^]^ In comparison to the disubstituted *ortho*‐carborane substituted celecoxib derivative reported by Zhang et al.^[^ ^44^ ^]^ a longer alkylene spacer in **9** led to loss of COX‐2 inhibition.

In general, the four *nido*‐carborane derivatives **7**, **10**, and **14a,b** showed better COX inhibition results, than their corresponding *ortho*‐carborane analogs **6**, **9**, and **13a,b**, with **10** exhibiting the best inhibition of all eight compounds toward COX‐2 (IC_50_ = 0.34 μM) with a slight selectivity for COX‐2 (SI = 10.3). Therefore, in future, a focus on conjugates with *nido*‐carborane could be promising.

## 4 Experimental Section

4.1

4.1.1

##### General Information

Reactions with carborane derivatives were usually carried out under nitrogen atmosphere, using Schlenk technique. Anhydrous Et_2_O and toluene were obtained with a solvent purification system (MBRAUN, MBraun Inertgas‐Systeme GmbH, Garching, Germany). DMF was dried over calcium hydride and distilled. THF was distilled from potassium/benzophenone. Dry solvents were stored over molecular sieves (4 or 5 Å) under a nitrogen atmosphere. TBDMS‐protected carboranyl‐propanol **3** was prepared according to the literature.^[^ ^46^, ^47^, ^48^ ^]^ Tosylation of the hydroxy group in **4** was performed as published.^[^ ^49^, ^50^ ^]^ Compounds **12a,b** were synthesized according to Brown et al.^[^ ^53^ ^]^ Chappie et al.^[^ ^54^ ^]^ and Merkul et al.^[^ ^55^ ^]^ All other solvents and chemicals were commercially available and used without further purification. Microwave reactions were performed within an Initiator + microwave from Biotage (Uppsala, Sweden). Reaction progress and purification were monitored by thin‐layer chromatography (TLC) using precoated silica gel 60 F_254_ Alugram plates (Xtra SIL G) from Macherey‐Nagel (Düren, Germany). Carborane‐containing TLC spots were stained with a 5%–10% PdCl_2_ solution in methanol. Chromatography was performed in air, with silica gel (60 Å, 0.035–0.070 mm particle diameter) or in an automated fashion with Isolera‐4 and an evaporative light‐scattering detector (ELSD) 1080 (Biotage) with commercially available solvents. Nuclear magnetic resonance (NMR) spectra were recorded with a Varian MERCURYplus 300 or 400 spectrometer, a Bruker AVANCE III HD 400, a Bruker Fourier 300 spectrometer or an Agilent DD2‐400 spectrometer. Measurements were performed at 400 MHz (^1^H), 128 MHz (^11^B), 76 MHz or 101 MHz (^13^C), and 376 MHz (^19^F). Chemical shifts (*δ*) are given in parts per million (ppm). ^1^H and ^13^C NMR spectra were referenced to internal deuterated solvent and ^11^B{^1^H} NMR spectra to the Ξ‐scale.^[^ ^60^ ^]^ Deuterated solvents (CDCl_3_, CD_3_OD, CO(CD_3_)_2_) were purchased from Eurisotop (Saint‐Aubin, France) with a deuteration rate of 99.80%. High‐resolution mass spectrometry (HRMS) was conducted in either positive or negative ion mode with an electrospray ionization (ESI)‐TOF microTOF instrument from Bruker Daltonik GmbH (Bremen, Germany). The target compounds were therefore dissolved in CH_3_CN (*ortho*‐carborane compounds) or MeOH (*nido*‐carborane analogs). The simulation of mass spectra was carried out with the Web‐based MS online tool of Scientific Instrument Service (SISweb, Palmer, MA). The analysis of NMR and MS data was done with MestReNova version 14.1.0.^[^ ^61^ ^]^ X‐ray analysis was performed with single crystals, obtained from CDCl_3_ at rt by slow evaporation of the solvent. The crystals were measured on a Gemini diffractometer (Rigaku Oxford Diffraction) with Mo K*α* radiation (*λ* = 71.073 pm) in *ω*‐scan mode. Data reduction was performed with CrysAlis^Pro^.^[^ ^62^ ^]^ Empirical absorption correction was performed with SCALE3 ABSPACK.^[^ ^63^ ^]^ Structure solution and anisotropic refinement were performed with SHELXT^[^ ^64^ ^]^ and SHELXL.^[^ ^65^ ^]^ Further details are available in the Supporting Information (“5 X‐ray Crystallography Data of Compound **9**”).

##### Synthesis: Synthesis of Compounds 5 and 8

A solution of tosylate **4** (0.964 g, 2.05 mmol, 1.00 eq) in dry DMF (2.0 mL) was added dropwise to a suspension of celecoxib (1.56 g, 4.10 mmol, 2.00 eq) and Cs_2_CO_3_ (1.33 g, 4.10 mmol, 2.00 eq) in dry DMF (4.0 mL) at 0 °C. The reaction mixture was allowed to warm up to rt and stirred for 24 h. To the reaction mixture H_2_O (10 mL) was added, and the aqueous phase was washed with Et_2_O (3 × 15 mL). The combined organic layer was washed with brine, dried over MgSO_4_, and was filtered. The solvent was removed under reduced pressure, and the crude product was purified by column chromatography (*n*‐hexane/ethyl acetate (EtOAc), 4:1 (*v*/*v*)). Compound **5** (0.538 g, 0.81 mmol, 40%), a white solid, was formed as main product, and compound **8** (0.305 g, 0.31 mmol, 15%) was formed as side product.

##### Synthesis: Synthesis of compounds 6 and 9

Compound **5** or **8** was dissolved in dry THF. At −78 °C, TBAF·H_2_O was added. After 40 min, it was allowed to warm up to 0 °C. After complete consumption, which was checked via TLC, H_2_O (3 mL) was added and it was washed with Et_2_O (3 × 10 mL). The organic layer was washed with brine, dried over MgSO_4_, filtered, and was concentrated. The crude was purified by column chromatography.

##### Synthesis: *N*‐[3‐(1,2‐*closo*‐dicarbadodecaborane)propyl]‐4‐[5‐(*p*‐tolyl)‐3‐(trifluoromethyl)‐1*H*‐pyrazol‐1‐yl]benzenesulfonamide (6)

(**5**: 0.280 g, 0.41 mmol, 1.00 eq; dry THF: 1.37 mL, 0.3 M; TBAF·H_2_O: 0.156 g, 0.49 mmol, 1.20 eq; column chromatography: *n*‐hexane/EtOAc 2:1 (*v*/*v*)). The white crystalline solid **6** (0.226 g, 0.40 mmol) was obtained in 97% yield. ^1^H NMR (400 MHz, CDCl_3_) *δ* 7.81 (d, ^3^ *J* = 8.3 Hz, 2H, ar‐CH), 7.49 (d, ^3^ *J* = 8.3 Hz, 2H, ar‐CH), 7.18 (d, ^3^ *J* = 7.9 Hz, 2H, ar‐CH), 7.11 (d, ^3^ *J* = 7.8 Hz, 2H, ar‐CH), 6.75 (s, 1H, ar‐CH), 4.87 (t, ^3^ *J* = 6.4 Hz, 1H, NH), 3.60 (s, 1H, carborane‐CH), 2.91 (q, ^3^ *J* = 6.4 Hz, 2H, CH_2_), 2.38 (s, 3H, CH_3_), 2.26 (m, 2H, CH_2_), 1.69 (m, 2H, CH_2_), 0.69‐3.24 (br, 10H, BH); ^11^B{^1^H} NMR (128 MHz, CDCl_3_) *δ* −2.3 (s, 1B), −5.6 (s, 1B), −9.3 (s, 1B), −12.5 (overlapping s, 7B); ^13^C{^1^H} NMR (101 MHz, CDCl_3_) *δ* 145.5 (C_q_), 144.4 (q, *J* = 38.4 Hz, C_q_), 143.0 (C_q_), 140.1 (C_q_), 139.0 (C_q_), 129.9 (ar‐CH), 128.8 (ar‐CH), 128.1 (ar‐CH), 125.8 (ar‐CH), 106.6 (ar‐CH), 74.3 (C_q_), 61.8 (carborane‐CH), 42.3 (CH_2_), 35.1 (CH_2_), 29.5 (CH_2_), 21.5 (CH_3_); ^19^ F{^1^H} NMR (376 MHz, CDCl_3_) *δ* −62.5; HRMS (ESI+) *m*/*z* for C_22_H_31_B_10_F_3_N_3_O_2_S [M + H]^+^ 566.3082, calcd 566.3092.

##### Synthesis: *N,N*‐Bis[3‐(1,2‐*closo*‐dicarbadodecaborane)propyl]‐4‐[5‐(*p*‐tolyl)‐3‐(trifluoromethyl)‐1*H*‐pyrazol‐1‐yl]benzenesulfonamide (9)

(**8**: 0.218 g 0.22 mmol, 1.00 eq; dry THF: 0.74 mL, 0.3 M; TBAF·H_2_O: 0.169 g, 0.54 mmol, 2.40 eq; column chromatography: *n*‐hexane/EtOAc 3:1 (*v*/*v*). The pale yellow solid **9** (0.142 g, 0.19 mmol) was obtained in 84% yield. ^1^H NMR (400 MHz, CDCl_3_) *δ* 7.72 (m, 2H, ar‐CH), 7.50 (m, 2H, ar‐CH), 7.19 (m, 2H, ar‐CH), 7.11 (m, 2H. ar‐CH), 6.75 (s, 1H, ar‐CH), 3.60 (s, 2H, 2 carborane‐CH), 3.01 (t, ^3^ *J* = 6.9 Hz, 4H, 2 CH_2_), 2.39 (s, 3H, CH_3_), 2.23 (m, 4H, 2 CH_2_), 1.73 (m, 4H, 2 CH_2_), 0.67‐3.24 (br, 20H, BH); ^11^B{^1^H} NMR (128 MHz, CDCl_3_) *δ* −2.2 (s, 2B); −5.5 (s, 2B), −9.2 (s, 3B), −12.4 (overlapping s, 13B); ^13^C{^1^H} NMR (101 MHz, CDCl_3_) *δ* 145.5 (C_q_), 144.5 (m, C_q_), 143.2 (C_q_), 140.1 (C_q_), 137.9 (C_q_), 130.0 (ar‐CH), 128.9 (ar‐CH), 128.2 (ar‐CH), 125.9 (ar‐CH), 106.6 (ar‐CH), 74.1 (C_q_), 61.8 (carborane‐CH), 48.6 (CH_2_), 35.2 (CH_2_), 29.0 (CH_2_), 21.5 (CH_3_); ^19^ F{^1^H} NMR (377 MHz, CDCl_3_) *δ* −62.5; HRMS (ESI+) *m*/*z* for C_27_H_47_B_20_F_3_N_3_O_2_S [M + H]^+^ 751.5341, calcd 751.5311.

##### Synthesis: Synthesis of Compounds 13a and 13b

Chlorobenzene (3.4 mL) and *N*,*N*‐dimethylaniline were added under N_2_ to a microwave vial filled with the respective compound **12a** or **12b** and decaborane. The yellow suspension was stirred at rt for 1 min and at 120 °C–130 °C for 30 min in the microwave (780 rpm). The reaction mixture was washed with EtOAc and water three times. The combined organic layer was washed with aqueous HCl (0.5 N) and brine and was dried over Na_2_SO_4_ followed by filtration. The solvent was removed under reduced pressure, and the crude product was purified by column chromatography.

##### Synthesis: (*R/S*)‐2‐[2‐fluoro‐(1,1′‐biphenyl)‐4‐yl]‐*N*‐(1,2‐*closo*‐dicarbadodecaborane)‐methylpropanamide (13a)

(**12a**: 100.0 mg, 0.355 mmol, 1.00 eq; decaborane: 70.5 mg, 0.577 mmol, 1.62 eq; *N*,*N*‐dimethylaniline: 0.15 mL, 144 mg, 1.18 mmol, 3.33 eq; column chromatography: *n*‐hexane/EtOAc, 83:17 (*v*/*v*) → 100% EtOAc). Compound **13a** (65.4 mg, 0.164 mmol) was obtained as a white solid in 46% yield. ^1^H NMR (400 MHz, CDCl_3_) *δ* 7.54 (d, ^3^ *J* = 7.4 Hz, 2H, ar‐CH), 7.46 (td, ^3^ *J* = 7.8, 3.0 Hz, 3H, ar‐CH), 7.39 (t, ^3^ *J* = 7.3 Hz, 1H, ar‐CH), 7.12 (m, 2H, ar‐CH), 6.20 (t, ^3^ *J* = 6.8 Hz, 1H, NH), 3.86 (d, ^3^ *J* = 6.6 Hz, 3H, carborane‐CH, CH_2_), 3.61 (q, ^3^ *J* = 7.1 Hz, 1H, CH), 1.56 (d, ^3^ *J* = 7.1 Hz, 3H, CH_3_), 0.35‐3.10 (br, 10H, BH); ^11^B{^1^H} NMR (128 MHz, CDCl_3_) *δ* −1.7 (s, 1B), −5.0 (s, 1B), −9.7 (s, 2B), −12.3 (overlapping s, 6B); ^13^C{^1^H} NMR (101 MHz, CDCl_3_) *δ* 174.5 (C_q_), 161.3 (C_q_), 158.8 (C_q_), 141.6 (d, *J* = 7.4 Hz, C_q_), 135.3 (d, *J* = 1.3 Hz, C_q_), 131.6 (d, *J* = 4.0 Hz, ar‐CH), 129.1 (d, *J* = 2.9 Hz, ar‐CH), 128.7 (ar‐CH), 128.1 (ar‐CH), 123.7 (d, *J* = 3.4 Hz, ar‐CH), 115.5 (d, *J* = 23.6 Hz, ar‐CH), 74.9 (C_q_), 60.5 (carborane‐CH), 46.4 (d, *J* = 1.5 Hz, CH), 44.7 (CH_2_), 18.3 (CH_3_); ^19^ F{^1^H} NMR (377 MHz, CDCl_3_) *δ* −116.5; HRMS (ESI–) *m*/*z* for C_18_H_26_B_10_ClFNO [M + Cl]^−^ 435.2650, calcd 435.2654.

##### Synthesis: (*R/S*)‐2‐[2‐fluoro‐(1,1′‐biphenyl)‐4‐yl]‐*N*‐(1,2‐*closo*‐dicarbadodecaborane)‐ethylpropanamide (13b)

(**12b**: 130.2 mg, 0.441 mmol, 1.00 eq; decaborane: 85.1 mg, 0.696 mmol, 1.58 eq; *N*,*N*‐dimethylaniline: 0.17 mL, 163 mg, 1.35 mmol, 3.06 eq; column chromatography: *n*‐hexane/EtOAc, 80:20 (*v*/*v*) → 100% EtOAc and *n*‐hexane/EtOAc, 80:20 (*v*/*v*) → 50% EtOAc (twice)). Compound **13b** (72.6 mg, 0.176 mmol) was obtained as a white solid in 40% yield. ^1^H NMR (400 MHz, CDCl_3_) *δ* 7.53 (m, 2H, ar‐CH), 7.44 (m, 3H, ar‐CH), 7.39 (m, 1H, ar‐CH), 7.10 (m, 2H, ar‐CH), 5.61 (t, ^3^ *J* = 6.1 Hz, 1H, NH), 3.55 (m, 2H, carborane‐CH, CH), 3.34 (m, ^3^ *J* = 6.9 Hz, 2H, CH_2_), 2.40 (t, ^3^ *J* = 7.0 Hz, 2H, CH_2_), 1.54 (d, ^3^ *J* = 7.2 Hz, 3H, CH_3_), 0.62‐3.08 (br, 10H, BH); ^11^B{^1^H} NMR (128 MHz, CDCl_3_) *δ* −2.2 (s, 1B), −5.4 (s, 1B), −9.2 (s, 2B), −12.5 (overlapping s, 6B); ^13^C{^1^H} NMR (101 MHz, CDCl_3_) *δ* 173.9 (C_q_), 161.3 (m, C_q_), 158.8 (C_q_), 142.1 (d, *J* = 7.4 Hz, C_q_), 135.3 (C_q_), 131.5 (d, *J* = 4.0 Hz, ar‐CH), 129.1 (d, *J* = 2.9 Hz, ar‐CH), 128.7 (ar‐CH), 128.0 (ar‐CH), 123.7 (d, *J* = 3.4 Hz, ar‐CH), 115.4 (d, *J* = 23.6 Hz, ar‐CH), 72.7 (C_q_), 61.6 (carborane‐CH), 46.7 (d, *J* = 1.5 Hz, CH), 38.9 (CH_2_), 37.2 (CH_2_), 18.3 (CH_3_); ^19^ F{^1^H} NMR (377 MHz, CDCl_3_) *δ* −116.6; HRMS (ESI+) *m*/*z* for C_19_H_29_B_10_FNO [M + H]^+^ 414.3209, calcd 414.3236.

##### Synthesis: General Procedure for the Synthesis of  *nido*‐Carborane Derivatives

A microwave vial was filled with the respective *ortho*‐carborane **derivative 6, 9**, **13a** or **13b**, CsF and a mixture of degassed EtOH/H_2_O (3.0 mL, 1:1, *v*/*v*). The suspension was stirred for 1 min at rt and 10 min at 150 °C in the microwave (780 rpm). The solvent was removed under reduced pressure, and the crude product was purified by column chromatography.

##### Synthesis: Cesium *N*‐[3‐(7,8‐*nido*‐dicarbadodecahydroundecaborate(−1))propyl]‐4‐[5‐(*p*‐tolyl)‐3‐(trifluoromethyl)‐1*H*‐pyrazol‐1‐yl]benzenesulfonamide (7, racemate)

(**6:** 41.5 mg, 0.073 mmol, 1.00 eq; CsF: 91.9 mg, 0.605 mmol, 8.25 eq; column chromatography: *n*‐hexane/EtOAc, 4:1 (*v*/*v*)  → 100% EtOAc, stationary phase was washed with MeOH, filtered, solvent was removed under reduced pressure). Compound **7** (29.7 mg, 0.043 mmol) was obtained in 59% yield as a white solid. ^1^H NMR (400 MHz, CO(CD_3_)_2_) *δ* 7.93 (d, ^3^ *J* = 8.3 Hz, 2H, ar‐CH), 7.57 (d, ^3^ *J* = 8.3 Hz, 2H, ar‐CH), 7.25 (s, 4H, ar‐CH), 6.98 (s, 1H, ar‐CH), 2.36 (s, 3H, CH_3_), 1.61 (m, 4H, 2 CH_2_), −0.52‐3.80 (br, 9H, BH), −2.72 (m, 1H, μ‐carborane‐CH); ^11^B{^1^H} NMR (128 MHz, CO(CD_3_)_2_) *δ* −12.1 (s, 2B), −15.0 (s, 1B), −17.7 (s, 1B), −19.7 (s, 2B), −23.0 (s, 1B), −34.3 (s, 1B), −38.2 (s, 1B); ^13^C{^1^ H} NMR (76 MHz, CO(CD_3_)_2_) *δ* 146.5 (C_q_), 144.0 (m, C_q_), 143.0 (C_q_), 141.9 (C_q_), 140.4 (C_q_), 130.5 (C_q_), 129.8 (ar‐CH), 128.9 (ar‐CH), 126.7 (ar‐CH), 106.7 (d, *J* = 2.0 Hz, ar‐CH), 44.4 (CH_2_), 37.6 (carborane‐CH), 32.0 (CH_2_), 21.3 (CH_3_); ^19^ F{^1^H} NMR (377 MHz, CO(CD_3_)_2_) *δ* −63.8; HRMS (ESI–) *m*/*z* for C_22_H_30_B_9_F_3_N_3_O_2_S [M–Cs ]^−^ 555.2888, calcd 555.2885.

##### Synthesis: Di‐cesium *N,N*‐bis[3‐(7,8‐*nido*‐dicarbadodecahydroundecaborate(−1))propyl]‐4‐[5‐(*p*‐tolyl)‐3‐(trifluoromethyl)‐1*H*‐pyrazol‐1‐yl]benzenesulfonamide (10, racemate)

(**9**: 68.0 mg, 0.091 mmol, 1.00 eq; CsF: 218.7 mg, 1.44 mmol, 15.9 eq; column chromatography: CH_2_Cl_2_/*n*‐hexane 1:1 (*v*/*v*) →  (9: 68.0 mg, 0.091 mmol, 1.00 eq; CsF: 218.7 mg, 1.44 mmol, 15.9 eq; column chromatography: CH_2_Cl_2_/*n*‐hexane 1:1 (*v*/*v*) → 100% CH_2_Cl_2_ → CH_2_Cl_2_/MeOH 9:1 (*v*/*v*), stationary phase was washed with MeOH, filtered, solvent was removed under reduced pressure). Compound **10** (62.7 mg, 0.063 mmol) was obtained in 70% yield as a white solid. ^1^H NMR (400 MHz, CD_3_OD) *δ* 7.99 (m, 2H, ar‐CH), 7.54 (m, 2H, ar‐CH), 7.23 (m, 2H, ar‐CH), 7.17 (m, 2H, ar‐CH), 6.89 (s, 1H, ar‐CH), 3.15 (m, 4H, 2 CH_2_), 2.36 (s, 3H, CH_3_), 1.63 (m, 8H, 4 CH_2_), −0.63‐3.06 (br, 18H, BH), −2.69 (s, 2H, 2 μ‐carborane‐CH); ^11^B{^1^H} NMR (128 MHz, CD_3_OD) *δ* −12.2 (s, 4B), −14.7 (s, 2B), −17.7 (s, 1B), −19.6 (overlapping s, 5B), −22.5 (s, 2B), −34.2 (s, 2B), −38.1 (s, 2B); ^13^C{^1^H} NMR (101 MHz, CD_3_OD) *δ* 147.4 (C_q_), 144.8 (m, C_q_), 143.6 (C_q_), 141.3 (m, C_q_), 141.2 (C_q_), 130.8 (ar‐CH), 130.1 (ar‐CH), 129.5 (ar‐CH), 127.4 (ar‐CH), 106.8 (ar‐CH), 37.8 (CH_2_), 30.8 (CH_2_), 21.5 (CH_3_); ^19^ F{^1^H} NMR (377 MHz, CD_3_OD) *δ* −64.6; HRMS (ESI–) *m*/*z* for C_27_H_47_B_18_F_3_N_3_O_2_S [M – 2Cs + H]^−^ 729.5133, calcd 729.5125.

##### Synthesis: Cesium (*R/S*)‐2‐[2‐fluoro‐(1,1′‐biphenyl)‐4‐yl]‐*N*‐(7,8‐*nido*‐dicarbadodecahydroundecaborate(−1))‐methylpropanamide (14a, racemate)

(**13a**: 38.1 mg, 0.095 mmol, 1.00 eq; CsF: 52.2 mg, 0.344 mmol, 3.6 eq; after reaction addition of EtOAc, solvent removed under reduced pressure, column chromatography four times: *n*‐hexane/EtOAc, 4:1 (*v*/*v*) → 100% EtOAc, *n*‐hexane/EtOAc, 1:1 (*v*/*v*) → 100% EtOAc, twice *n*‐hexane/CH_2_Cl_2_, 1:1 (*v*/*v*) → 100% CH_2_Cl_2_ → CH_2_Cl_2_/MeOH, 9:1 (*v*/*v*), after every column, the stationary phase was washed with EtOAc and MeOH or solely MeOH, filtered, solvent was removed under reduced pressure. An analytical sample of compound **14a** was further purified by semi‐preparative high‐performance liquid chromatography (HPLC) to provide **14a** in high purity for NMR measurements and COX assay. An HPLC (LC‐20 A Prominence HPLC by Shimadzu, consisting of degasser unit DGU‐20A5R, two separate pumping units LC‐A20R, sample manager SIC‐20ACHT, column oven CTO‐20AC, PDA‐detector SPD‐M20A, communication‐bus module CBM‐20 A, and fraction collector FRC‐10 A) was used. The chosen column was an Aeris Peptide 5 μm XB‐C_18_ column (100 Å, 250 mm × 21.2 mm. The eluent system consisted of water + 0.1% CF_3_COOH (eluent A) and CH_3_CN + 0.1% CF_3_COOH (eluent B) and the following gradient was applied: 0–3 min 50% eluent B, 3–34 min 50%–95% eluent B, 34–38 min 95% eluent B; 38–39 min 95%–50% eluent B; 39–42 min 50% eluent B. The flow rate was set to 10 mL min^−1^. The retention time was 19.5 min. Fractions were diluted with the 2.5‐fold volume of water and lyophilized. For ^1^H NMR measurement and COX assay, 0.522 mg (0.001 mmol) of compound **14a** was obtained as a white solid. The ^11^B{^1^H} and ^19^F{^1^H} NMR spectra have been measured before final purification with semipreparative column. The ^1^H NMR spectrum was measured afterward. ^1^H NMR (400 MHz, (CD_3_)_2_SO) *δ* 7.52 (d, ^3^ *J* = 7.6 Hz, 2H, ar‐CH), 7.43 (m, 4H, ar‐CH), 7.24 (m, 2H, ar‐CH), 3.76 (q, ^3^ *J* = 7.1 Hz, 1H, CH), −0.91–4.21 (br, 9H, BH); ^11^B{^1^H} NMR (128 MHz, CD_3_OD) *δ* −12.0 (s, 2B), −15.6 (s, 1B), −17.4 (s, 1B), −19.8 (s, 2B), −22.8 (s, 1B), −34.1 (s, 1B), −38.3 (overlapping s, 1B); ^19^ F{^1^H} NMR (377 MHz, CD_3_OD) *δ* −120.6, −120.7; HRMS (ESI–) *m*/*z* for C_18_H_26_B_9_FNO [M – Cs]^−^ 389.2899, calcd 389.2872.

##### Synthesis: Cesium (*R/S*)‐2‐[2‐fluoro‐(1,1'‐biphenyl)‐4‐yl]‐*N*‐(7,8‐*nido*‐dicarbadodecahydroundecaborate(−1))‐ethylpropanamide (14b, racemate)

(**13b**: 38.5 mg, 0.093 mmol, 1.00 eq; CsF: 264.9 mg, 1.74 mmol, 18.7 eq; column: *n*‐hexane/ EtOAc, 1:1 (*v*/*v*) → 100% EtOAc, stationary phase was washed with EtOAc and MeOH, filtered, solvent was removed under reduced pressure, second column: *n*‐hexane/CH_2_Cl_2_, 1:1 (*v*/*v*) → 100% CH_2_Cl_2_ → CH_2_Cl_2_/MeOH, 9:1 (*v*/*v*). An analytical sample of compound **14b** was further purified by semi‐preparative HPLC to provide **14b** in high purity for NMR measurements and COX assay. Compound **14b** was purified by HPLC analogously to compound **14a**. Multiple runs have been performed. The retention times were in a range between 19.2 min and 22.0 min. For ^1^H NMR measurements and COX assay, 0.785 mg (0.001 mmol) of **14b** was obtained as a white solid. The ^11^B{^1^H} and ^19^F{^1^H} NMR spectra have been measured before final purification with semipreparative column chromatography. The ^1^H‐NMR spectrum was measured afterward. ^1^H NMR (400 MHz, (CD_3_)_2_SO) *δ* 7.52 (d, ^3^ *J* = 7.6 Hz, 2H, ar‐CH), 7.43 (m, 4H, ar‐CH), 7.20 (m, 2H, ar‐CH), 3.59 (q, ^3^ *J* = 7.2 Hz, 1H, CH), 1.34 (d, ^3^ *J* = 7.0 Hz, 3H, CH_3_), −0.82‐4.49 (br, 9H, BH); ^11^B{^1^H} NMR (128 MHz, CD_3_OD) *δ* −12.5 (s, 2B), −15.0 (s, 1B), −17.3 (s, 1B), −20.4 (s, 1B), −22.4 (s, 1B), −34.2 (s, 1B), −38.4 (s, 2B); ^19^ F{^1^H} NMR (377 MHz, CD_3_OD) *δ* −120.6, −120.7; HRMS (ESI–) *m*/*z* for C_19_H_28_B_9_FNO [M – Cs]^−^ 403.3039, calcd 403.3029.

##### Purity Determination

The purity was determined with an high performance liquid chromatography ultra‐violet mass spectrometry (HPLC‐UV‐MS) system, consisting of an UltiMate 3000 UHPLC System (Thermo Scientific, Germering, Germany), with a DA detector (DAD‐3000RS), coupled to an MSQ Plus single quadrupole mass spectrometer from Thermo Scientific (Austin, TX, USA). A few milligrams of each compound were dissolved in 1 mL CH_3_CN for the *ortho*‐carborane derivatives or 1 mL MeOH or dimethyl sulfoxide (DMSO) for the *nido*‐carborane compounds. Approximately 50–200 μL of the stock solution were transferred into a separate vial and 5–20 μL were injected into the HPLC‐UV‐MS system and measured on a Poroshell 120 EC‐C_18_ column (100 × 3 mm, 2.7 μm) from Agilent Technologies (Waldbronn, Germany) at 25 °C. As eluents were used liquid chromatography mass spectrometry (LC‐MS) grade water + 0.1% formic acid (eluent A) and CH_3_CN + 0.1% formic acid (eluent B). The flow was set to 0.7 mL min^−1^, and the wavelengths monitored have been 254 nm, 320 nm and 366 nm. One measurement took 15 or 20 min. The elution method was as following: 0–1.5 min 40% eluent B, 1.5–10 min 40%–100% eluent B, for the 15 min method: 10–12 min 100% eluent B and 12–15 min 40% eluent B, for the 20 min method: 10–15 min 100% eluent B and 15–20 min 40% eluent B. Prior to every sample measurement, a blank run was performed with the corresponding pure solvent. The mass was detected in positive or negative electrospray ionization mode. For compound **14a** and **14b**, the following setup has been used. A UPLC I‐Class (Waters Corporation, Milford, MA, USA) was employed, comprising a binary gradient pump BSM, autosampler FTN, column manager CM, and diode array detector PDAeλ. These were coupled to the Waters Xevo TQ‐S, with a flow rate of 0.4 mL min^−1^ and an Aquity PREMIER Peptide BEH C_18_ column (Waters Corporation, Milford, MA, USA, 100 × 2.1 mm, 1.7 μm, 300 Å). The eluents were (A) water + 0.1% HOAc and (B) acetonitrile/methanol 1:1 (*v*/*v*) + 0.1% HOAc. The gradient was as follows: 0–0.5 min 45% eluent B, 0.5–5.5 min 45%–95% eluent B, 5.5–7 min 95% eluent B, 7–8 min 95%–45% eluent B, 8–8.5 min 45% eluent B. ESI mode was used throughout. The purity measurements have been performed on an analytical HPLC Agilent 1200 device (Agilent Technologies, Santa Clara, CA) equipped with a γ‐ray detector (Gabi, Raytest, Straubenhardt, Germany). The eluent system consisted of water + 0.1% (*v*/*v*) trifluoroacetic acid (eluent A) and CH_3_CN (eluent B). The HPLC system was based on a Puropher C18e (5 μm) Lichrochart 125‐3 cartridge (including the according pre‐column) and a gradient elution was performed: 0–0.5 min 45% eluent B, 0.5–7 min 45%–95% eluent B, 7–8.5 min 95% eluent B, 8.5–9.5 min 95%–45% eluent B, then 9.5–15 min 45% eluent B, flow: 0.75 mL min^−1^, column temperature 40 °C. All compounds had a purity ≥ 95%. The recorded chromatograms and spectra are available in the Supporting Information.

##### Stability Measurements

The stability was evaluated for all compounds in a mixture of DMSO, dried over molecular sieve, and LC‐MS grade water (1:1, *v*/*v*) with the same HPLC‐UV‐MS systems as described under “Purity Determination”. The column, eluent system, flow and MS tool were operated in the same way. For the stability determination, a few milligrams of each compound were dissolved in 1 mL DMSO. To dissolve the compounds, they were vortexed and heated up if necessary. The stock solution was further diluted with water. For that, 50–100 μL of the stock solution was transferred into a vial and the same amount of LC‐MS grade water was added. The two solvents were mixed thoroughly, and the measurement with HPLC‐UV‐MS was directly started. Additionally, measurements were performed in a varying time frame, from 15 or 20 min to 1 day. Usually, the injection volume was 10 μL, and before the measurement of the samples, a blank run with only DMSO/LC‐MS grade water (1:1, *v*/*v*) was performed. One measurement had a duration of 15 or 20 min (compounds **6, 7, 9, 10, 13a, 13b**) and 40 min (compounds **14a** and **14b**), respectively. For the latter compounds, the aforementioned system (Agilent 1200 system) and the following gradient was used: 0–3 min 45% eluent B, 3–28 min 45%–95% eluent B, 28–34 min 95% eluent B, 34–35 min 95%–45% eluent B, then 35–40 min 45% eluent B, flow: 0.75 mL min^−1^.

##### COX‐Inhibition Assays

For the cyclooxygenase assays, the fluorescence‐based COX assay “COX Fluorescent Inhibitor Screening Assay Kit” (Cayman Chemical Company, Ann Arbor, MI, USA) was used according to the manufacturer's instructions. The COX inhibition was determined against ovine COX‐1 and human recombinant COX‐2 as published elsewhere.^[^ ^66^ ^]^ As controls, celecoxib (COX‐2 selective) and SC‐560 (COX‐1 selective) have been used within the experiments. All compounds have been screened at a concentration of 100 μM (exception: compound **6**, concentration: 98 μM). For compounds showing more than ≈50 % inhibition in this screening, the IC_50_ values have been determined. IC_50_ values were determined with GraphPad Prism 10 version 10.2.3 by fitting to the equation *y* = A_2_ + (A_1_–A_2_)/(1 + (x/x_0_)^p^) and are given as absolute IC_50_ values. All concentrations have been tested at least in duplicate.

## Conflict of Interest

The authors declare no conflict of interest.

## Supporting information

Supplementary Material

## Data Availability

The data that support the findings of this study are available in the supplementary material of this article.
