# Supplementary material for: Carborane‐Based Analogs of Celecoxib and Flurbiprofen, their COX Inhibition Potential, and COX Selectivity Index
Source: ChemMedChem. 2025 May 4;20(11):e202500166. doi: 10.1002/cmdc.202500166 (PMC12132917; doi:10.1002/cmdc.202500166)
Supplement: Supplementary file 1 — Supplementary Material [file CMDC-20-e202500166-s001.pdf]

Supporting Information

Carborane-based Analogs of Celecoxib and Flurbiprofen, Their COX Inhibition Potential and COX Selectivity Index

Lea Ueberham,[a] Jonas Schädlich,[b, c] Kim Schramke,[a] Sebastian Braun,[a] Christoph Selg,[a] Markus Laube,[b] Peter Lönnecke,[a] Jens Pietzsch[b, c] and Evamarie Hey-Hawkins*[a, d]

[a] L. Ueberham, K. Schramke, Dr. S. Braun, Dr. C. Selg, Dr. P. Lönnecke, Prof. Dr. Dr. h.c. mult. E. Hey-Hawkins
Centre for Biotechnology and Biomedicine (BBZ)
Faculty of Chemistry and Mineralogy
Institute of Bioanalytical Chemistry
Universität Leipzig
Deutscher Platz 5, 04103 Leipzig, Germany
E-mail: hey@uni-leipzig.de

[b] J. Schädlich, Dr. M. Laube, Prof. Dr. J. Pietzsch
Department of Radiopharmaceutical and Chemical Biology
Institute of Radiopharmaceutical Cancer Research
Helmholtz-Zentrum Dresden-Rossendorf (HZDR)
Bautzner Landstraße 400, 01328 Dresden, Germany

[c] J. Schädlich, Prof. Dr. J. Pietzsch
School of Science
Faculty of Chemistry and Food Chemistry
Technische Universität Dresden
Mommsenstraße 4, 01062 Dresden, Germany

[d] Prof. Dr. Dr. h.c. mult. E. Hey-Hawkins
Faculty of Chemistry and Chemical engineering
Department of Chemistry
Babeș-Bolyai University
Str. Arany Janos Nr. 11, RO-400028 Cluj-Napoca, Romania

Contents

1 NMR Spectra of Compounds **6, 7, 9, 10, 13a, 13b, 14a, 14b** S2
2 HR-ESI Mass Spectra of Compounds **6, 7, 9, 10, 13a, 13b, 14a, 14b** S30
3 Purity Determination of Compounds **6, 7, 9, 10, 13a, 13b, 14a, 14b** by HPLC........ S35
4 Stability Determination of Compounds **6, 7, 9, 10, 13a, 13b, 14a, 14b** by HPLC S44
5 X-ray Crystallography Data of Compound **9**........ S49
6 COX Inhibition Assay........ S52

1 NMR Spectra of Compounds 6, 7, 9, 10, 13a, 13b, 14a, 14b

Compound 6

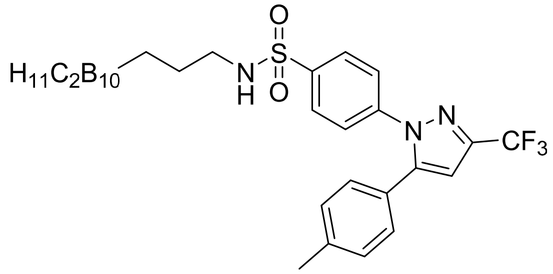

-

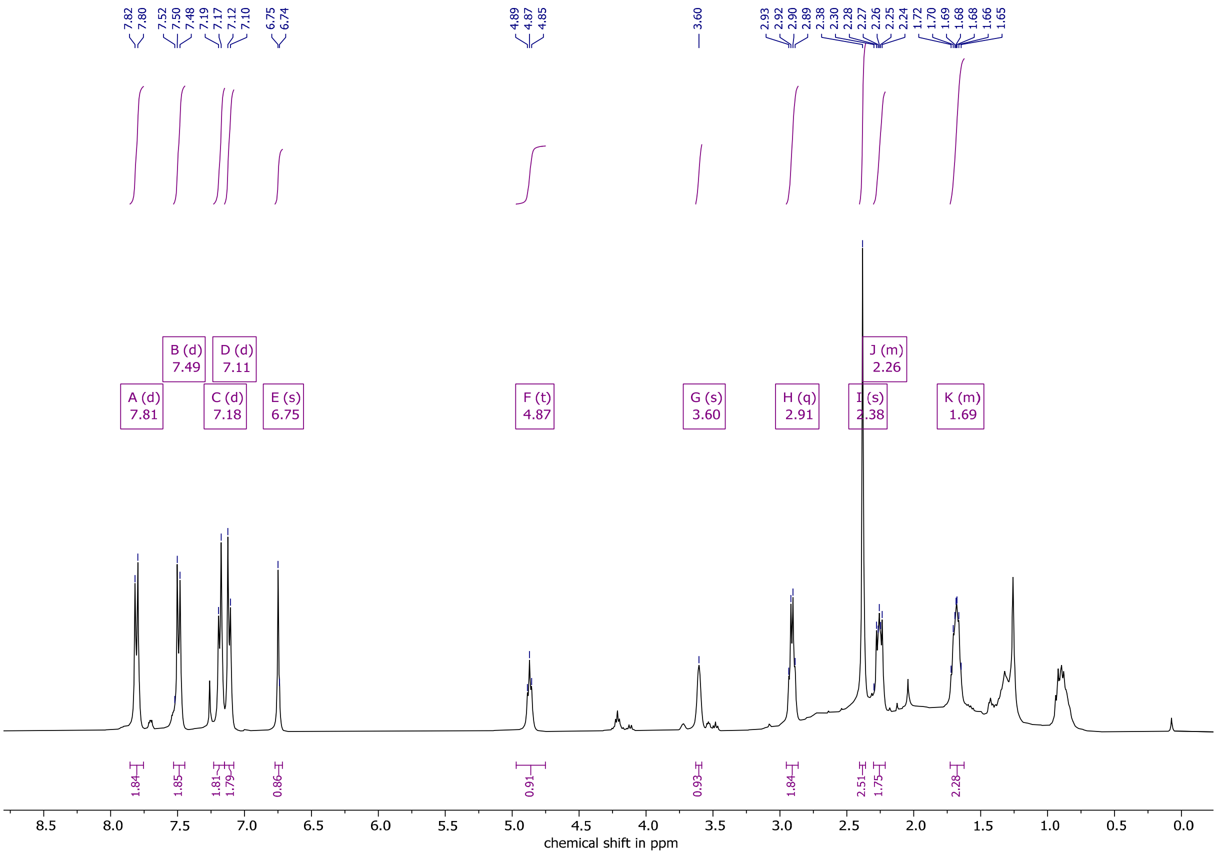

Figure S1. ^{1}H NMR spectrum of compound **6** in $CDCl_3$.

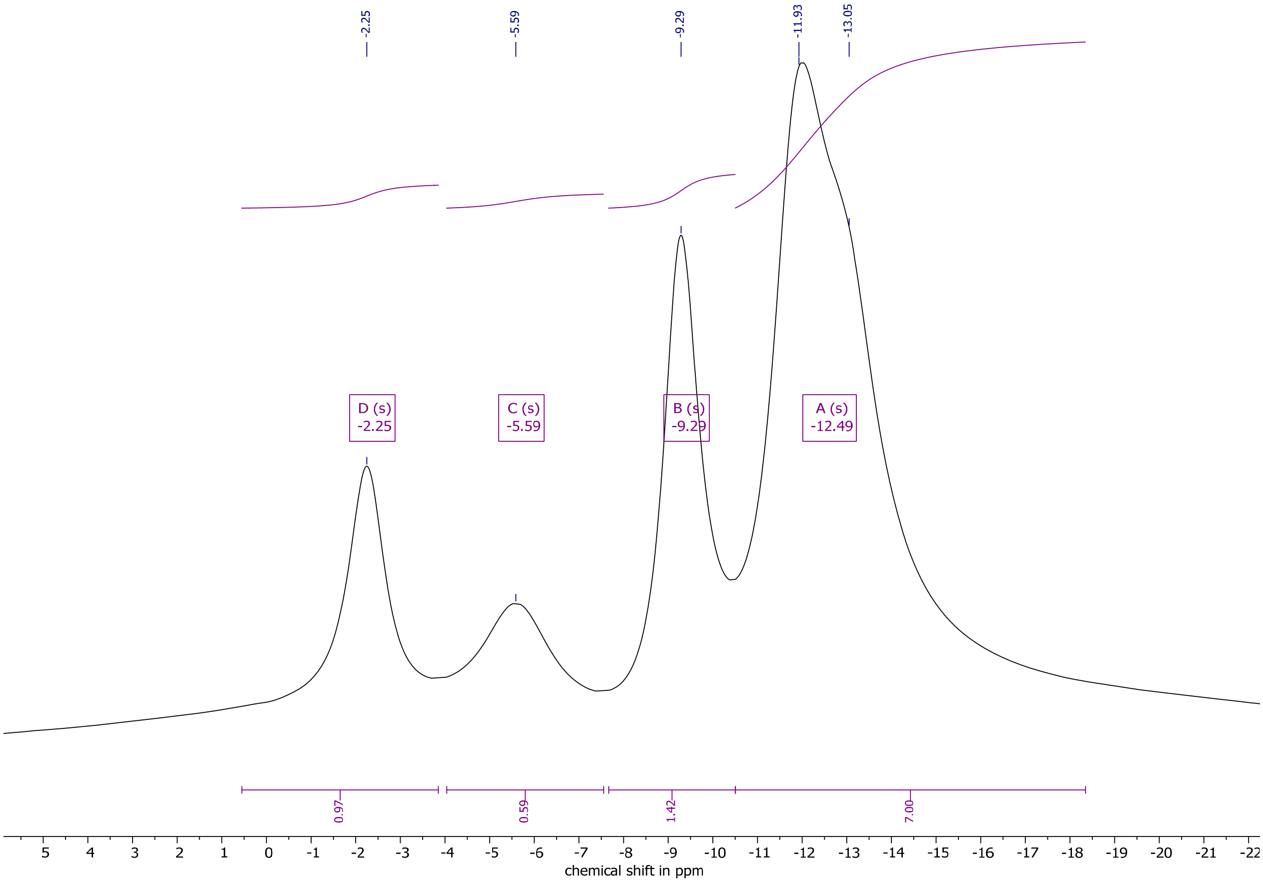

Figure S2.^{11}B{^{1}H} NMR spectrum of compound **6** in $CDCl_3$.

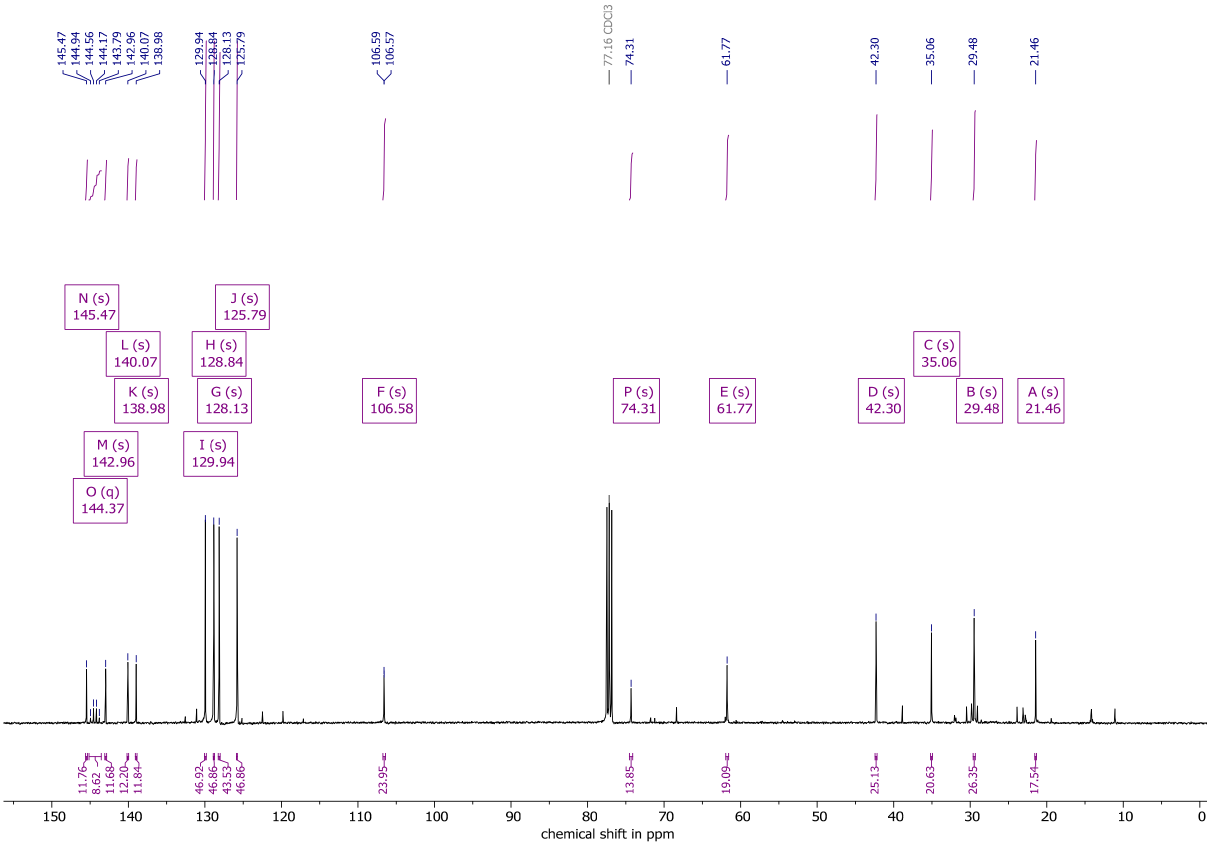

Figure S3. ^{13}C{^{1}H} NMR spectrum of compound **6** in $CDCl_3$.

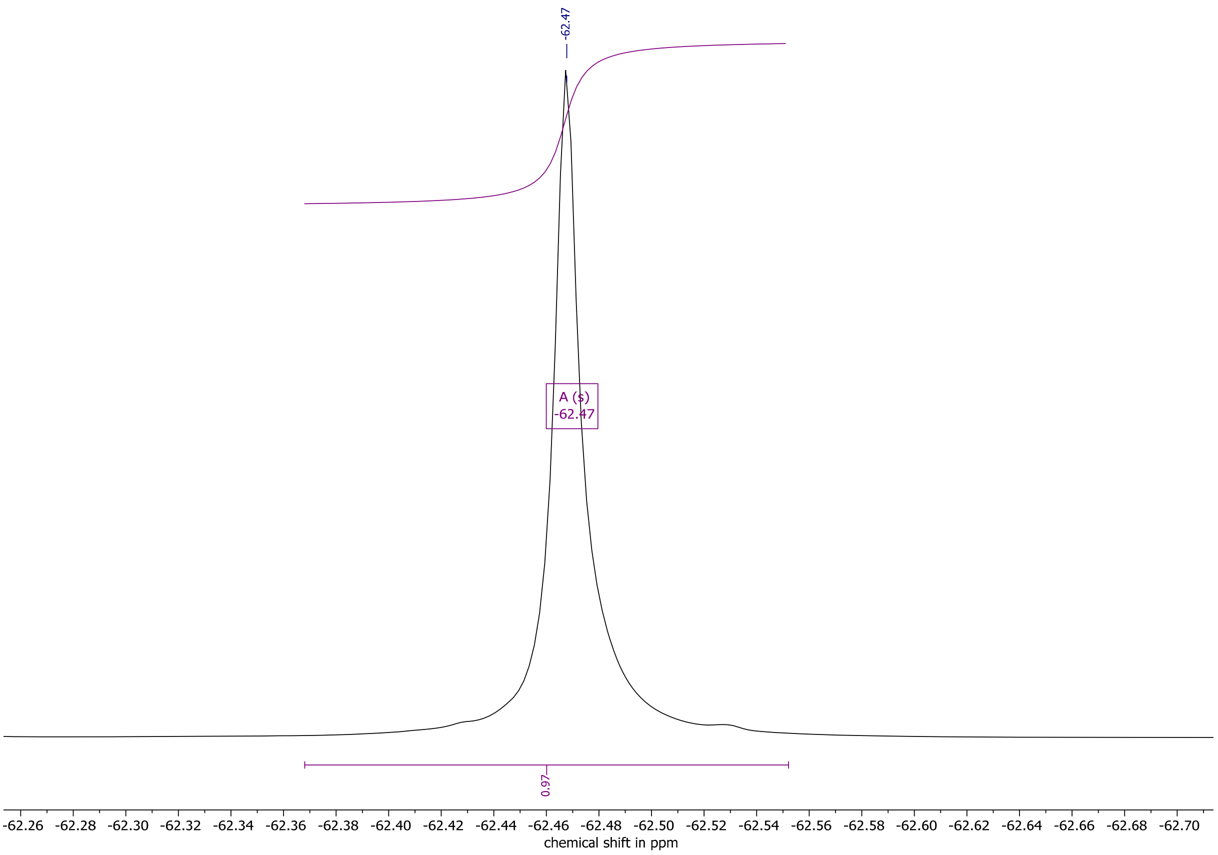

Figure S4. $^{19}F\{^{1}H\}$ NMR spectrum of compound **6** in $CDCl_3$.

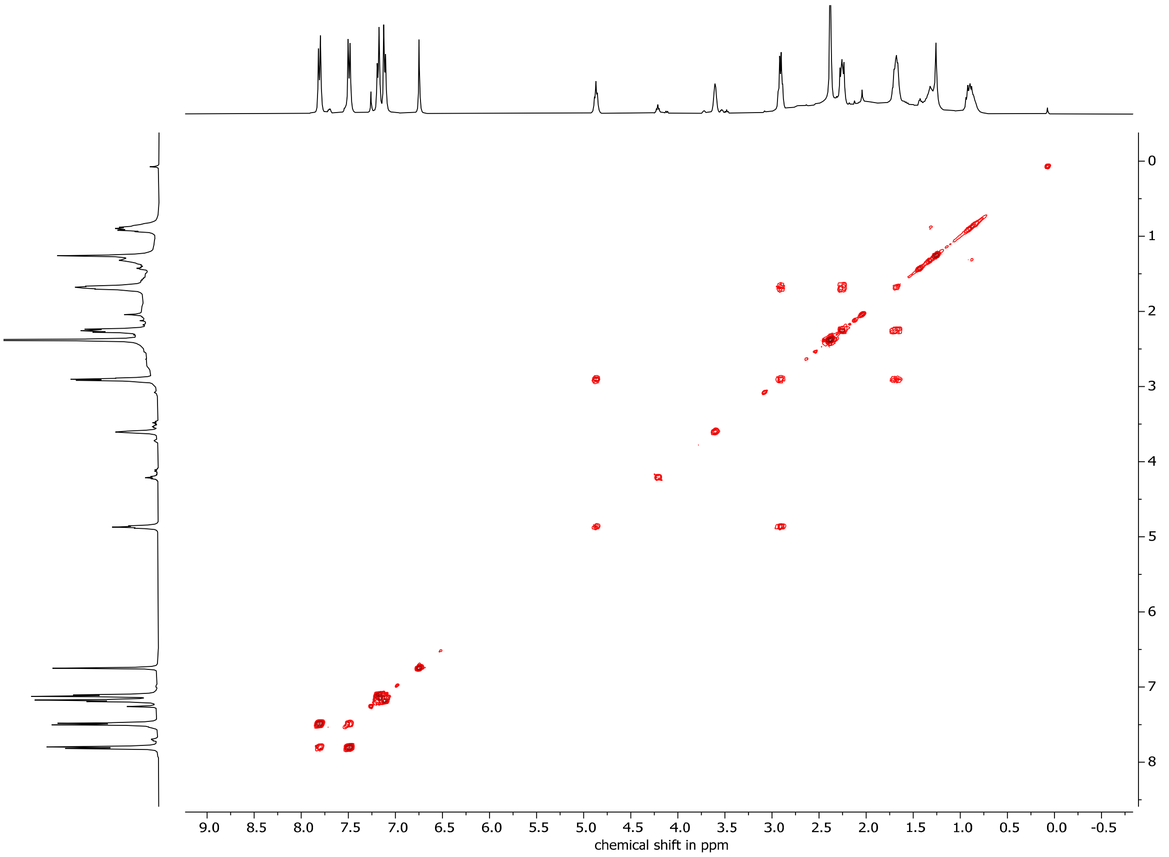

Figure S5. COSY (^{1}H, ^{1}H) NMR spectrum of compound **6** in $CDCl_3$.

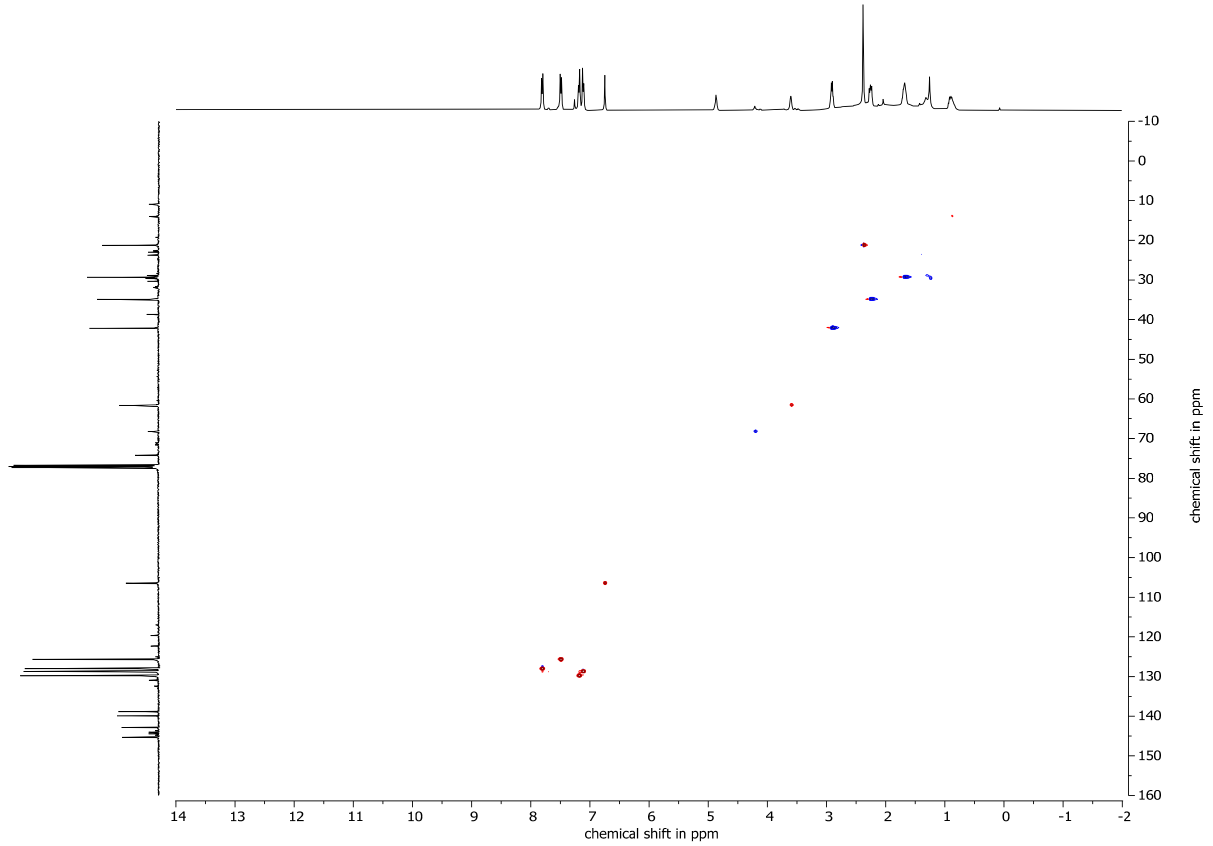

Figure S6. HSQC (^{1}H, ^{13}C) NMR spectrum of compound **6** in $CDCl_3$.

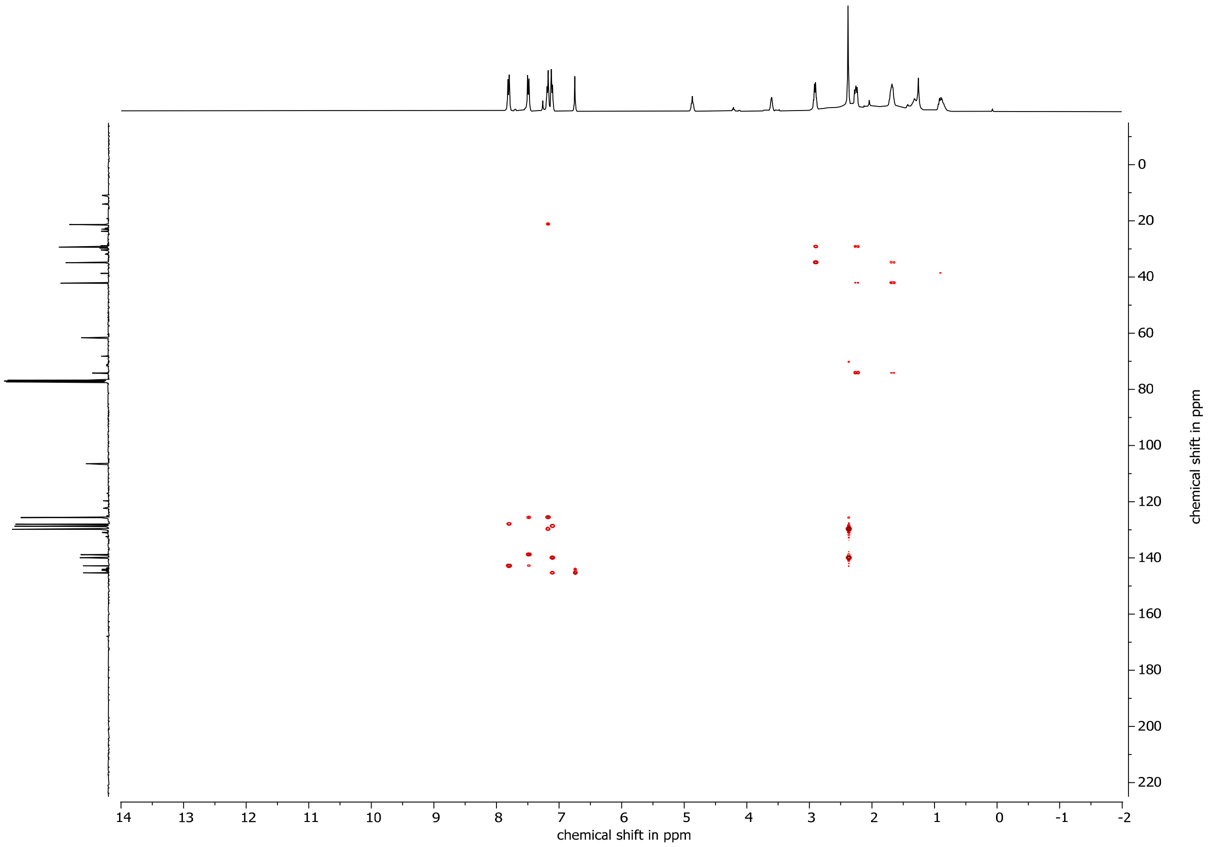

Figure S7. HMBC (^{1}H, ^{13}C) NMR spectrum of compound **6** in $CDCl_3$.

Compound 7

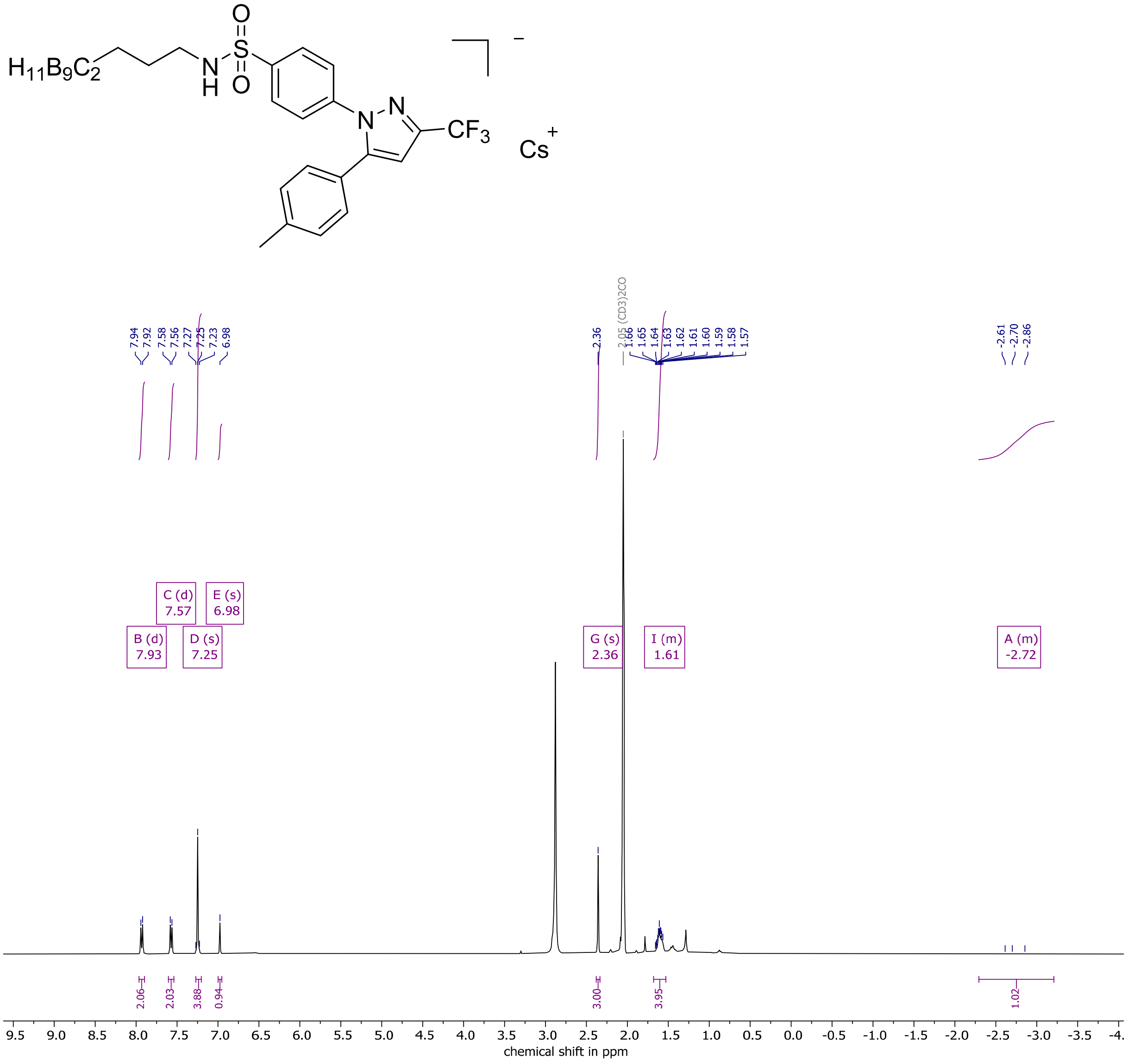

Figure S8. ^{1}H NMR spectrum of compound **7** in $CO(CD_3)_2$.

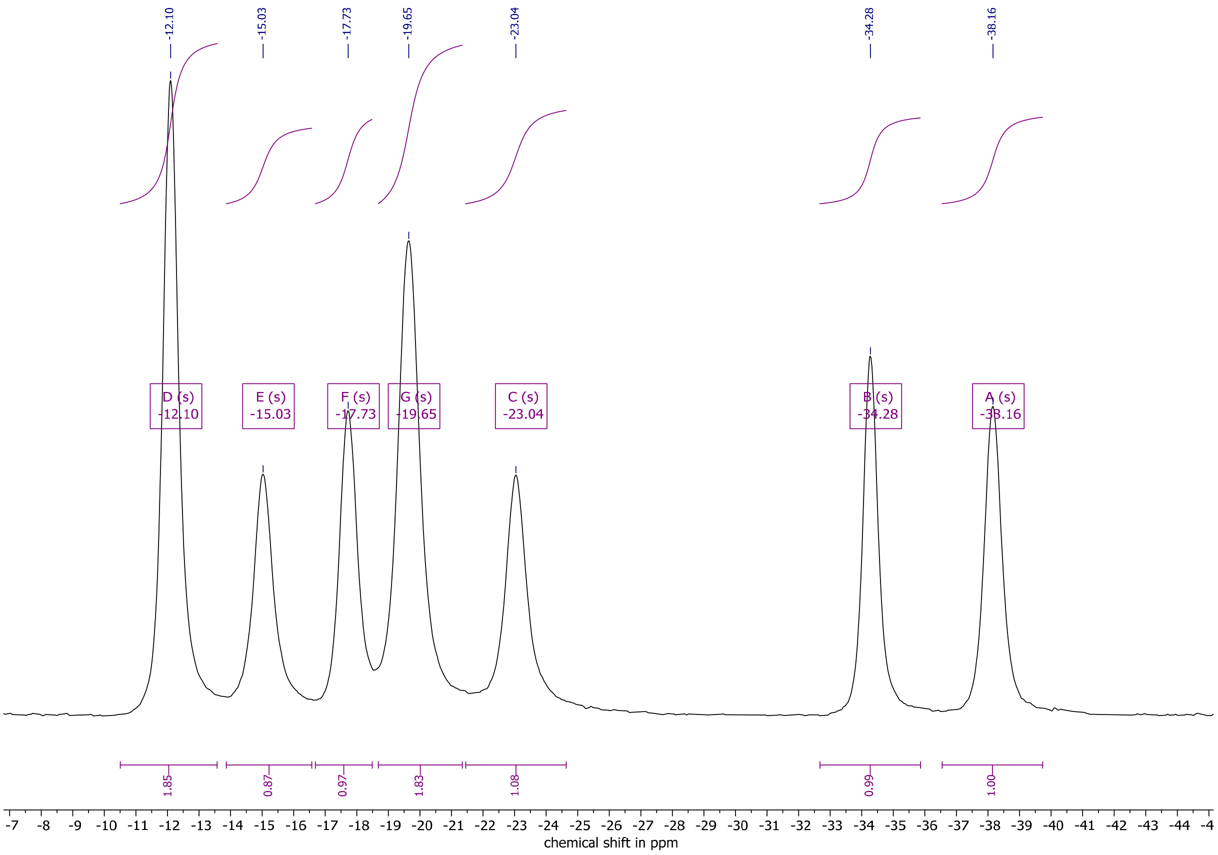

Figure S9. $^{11}B\{^{1}H\}$ NMR spectrum of compound **7** in $CO(CD_3)_2$.

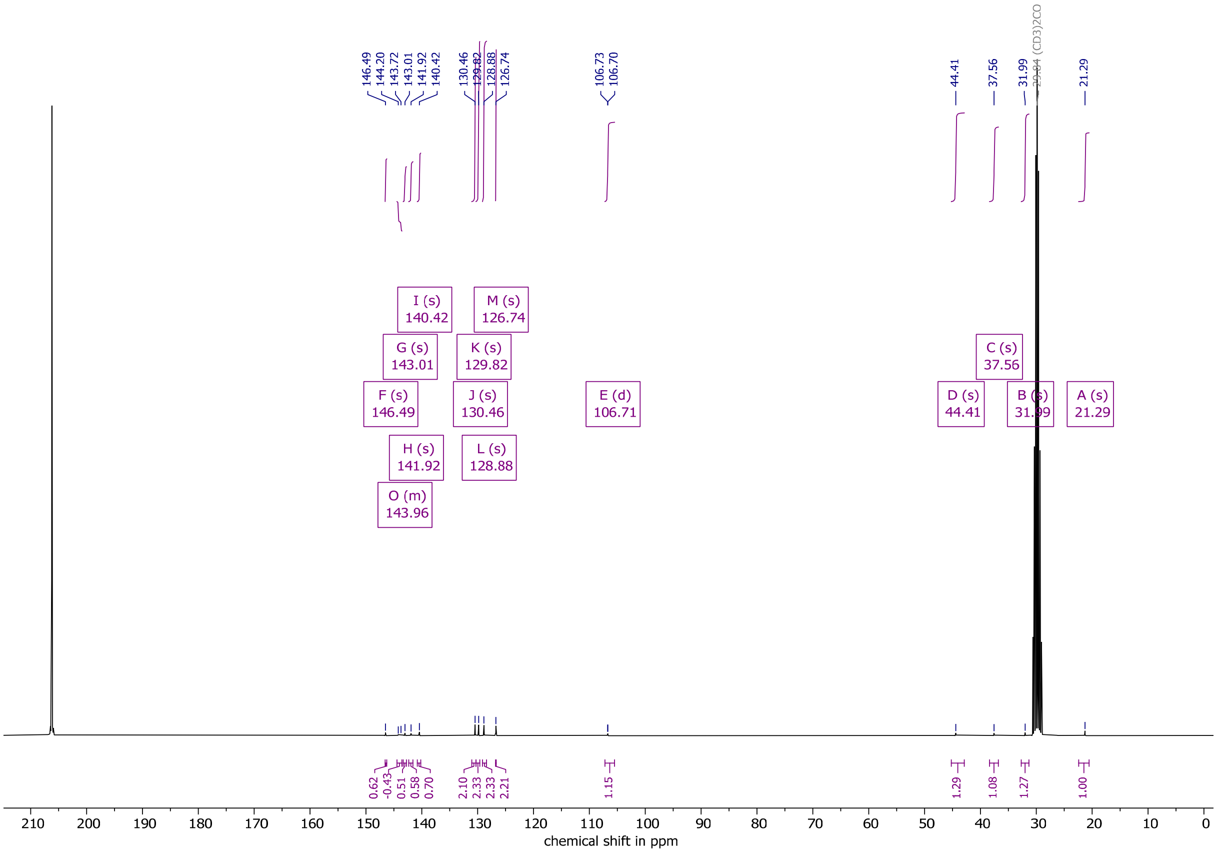

Figure S10. $^{13}C\{^{1}H\}$ NMR spectrum of compound **7** in $CO(CD_3)_2$.

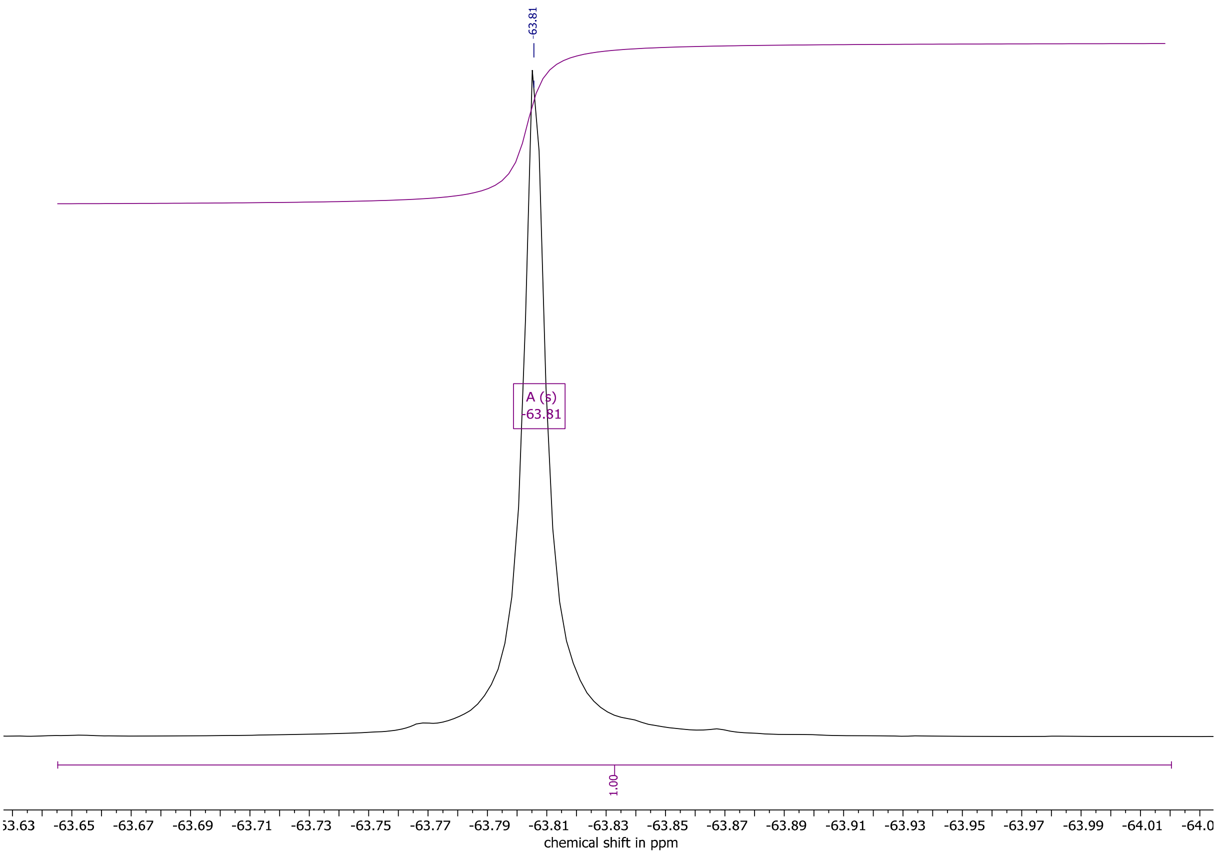

Figure S11. $^{19}F\{^{1}H\}$ NMR spectrum of compound **7** in $CO(CD_3)_2$.

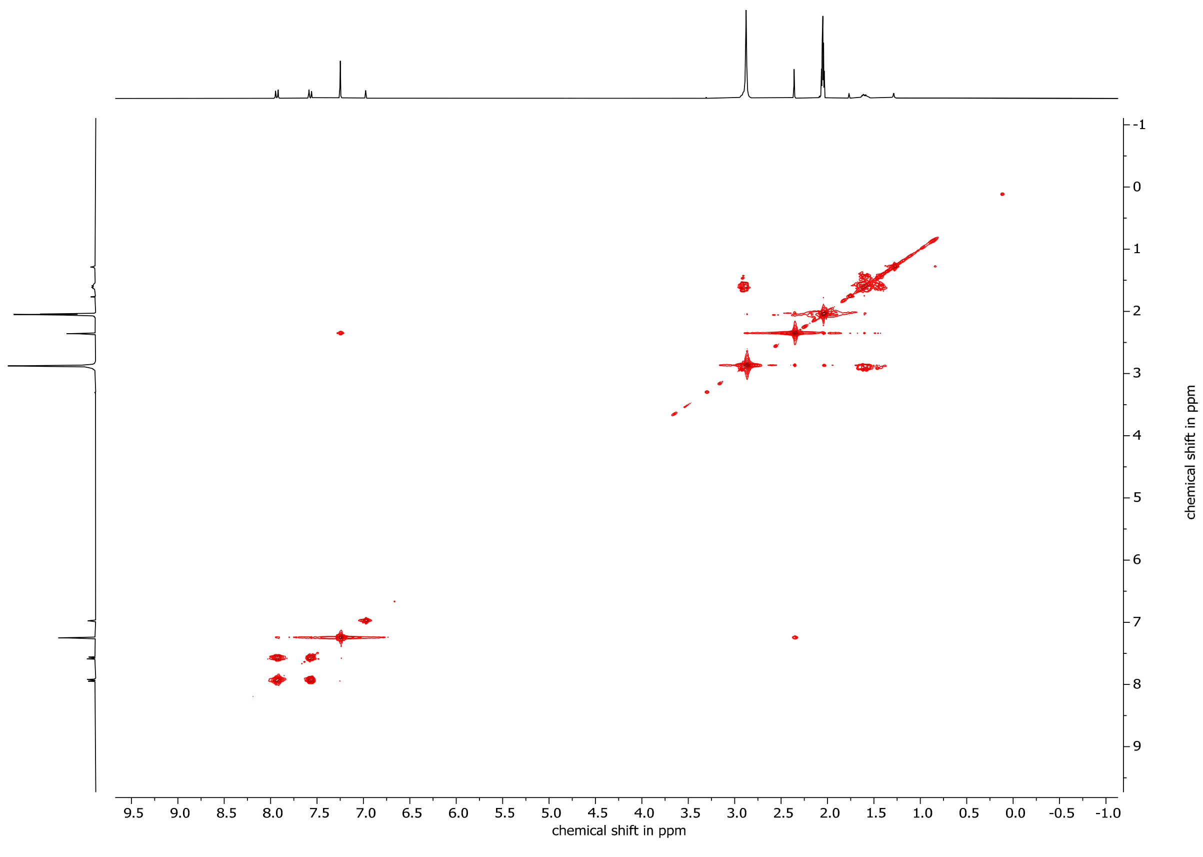

Figure S12. COSY (^{1}H, ^{1}H) NMR spectrum of compound **7** in $CO(CD_3)_2$.

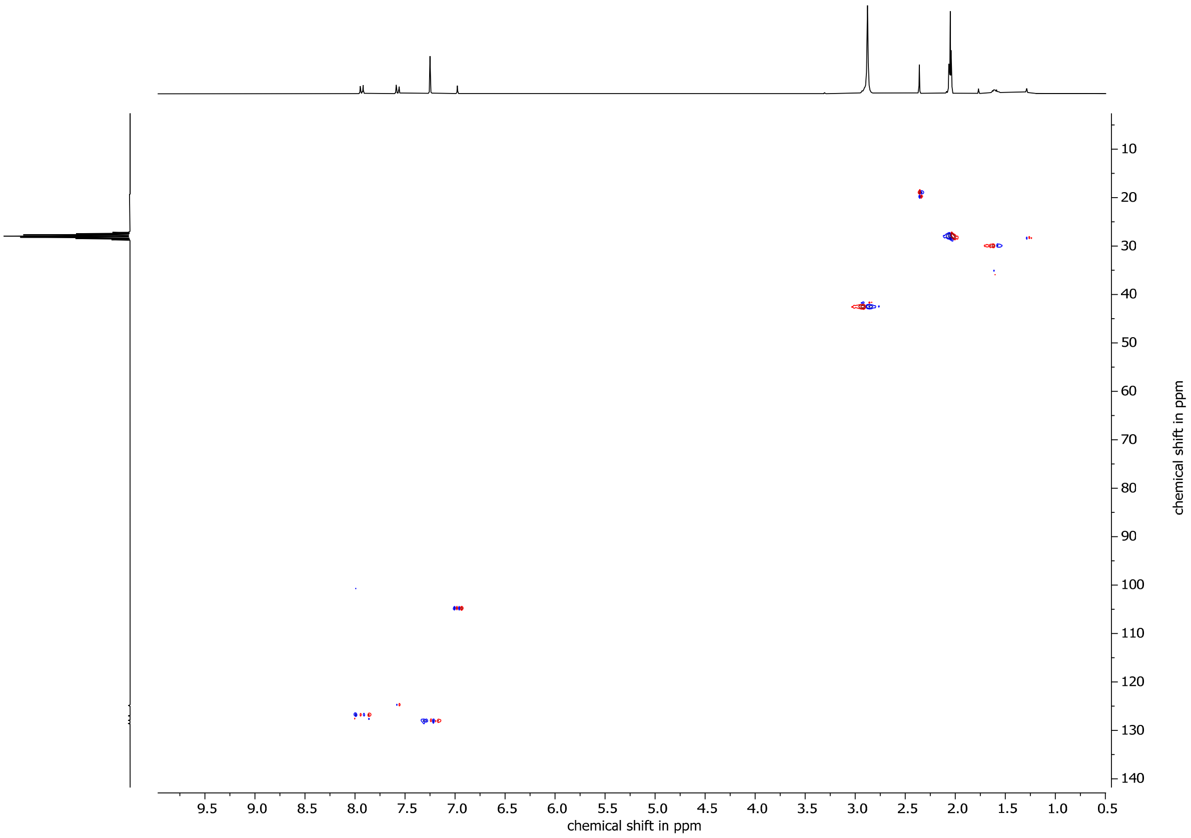

Figure S13. HSQC (^1H, ^{13}C) NMR spectrum of compound **7** in $CO(CD_3)_2$.

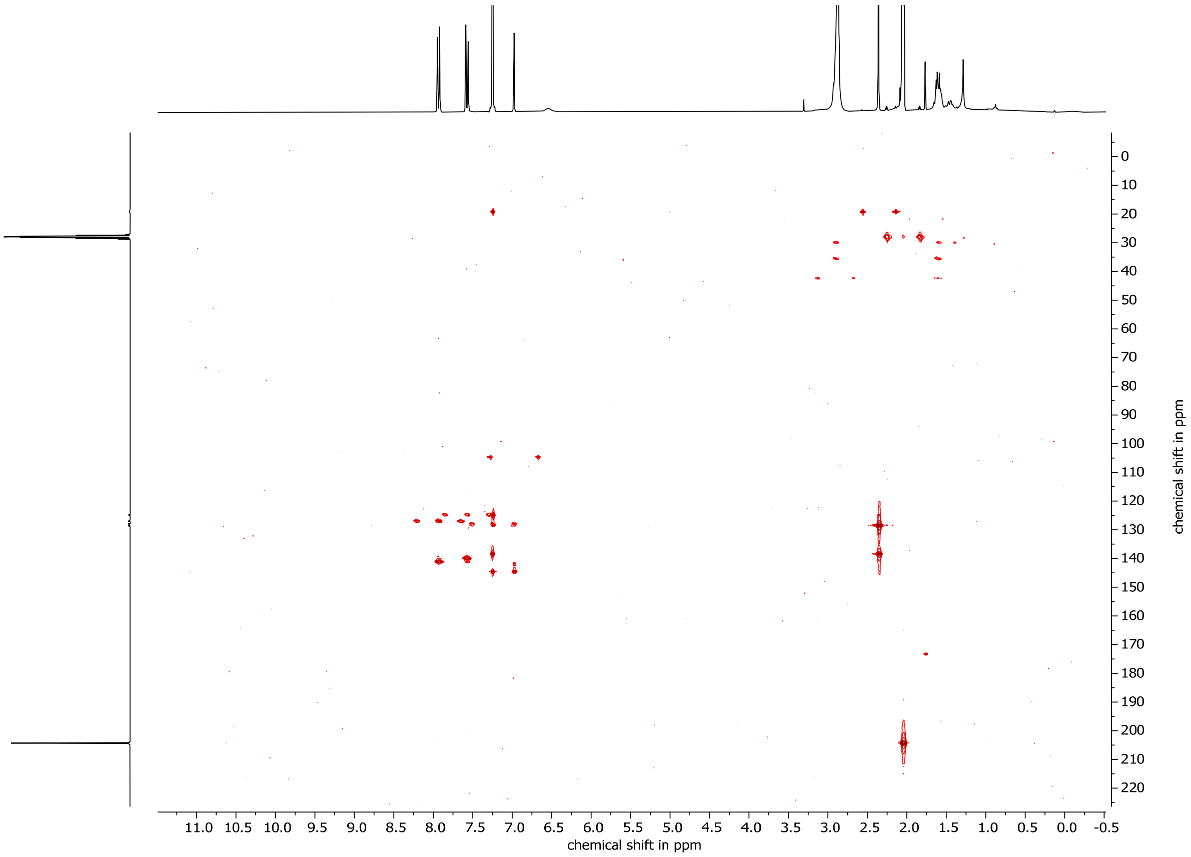

Figure S14. HMBC (^1H, ^{13}C) NMR spectrum of compound **7** in $CO(CD_3)_2$.

Compound 9

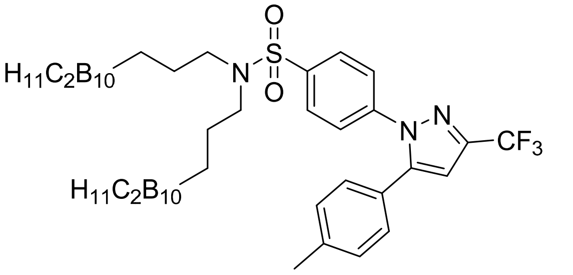

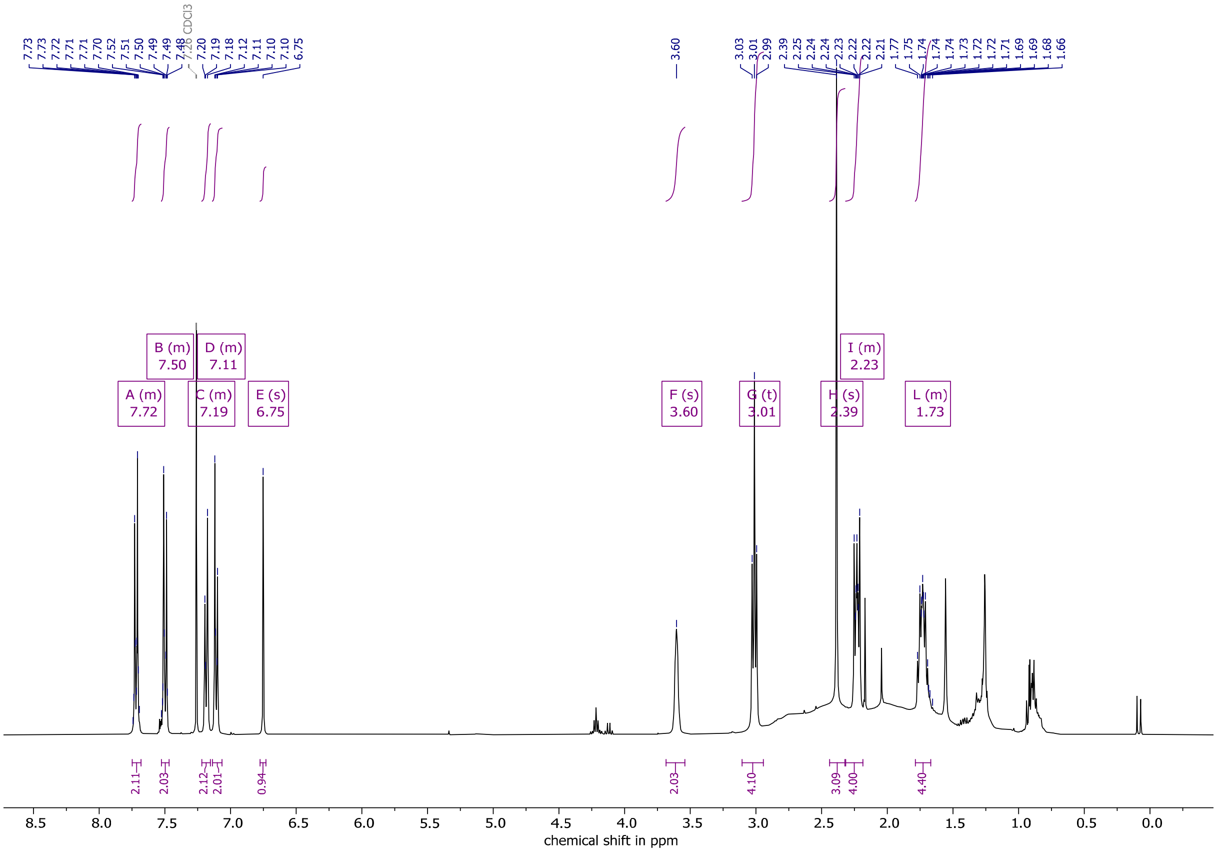

Figure S15. ^{1}H NMR spectrum of compound **9** in $CDCl_3$.

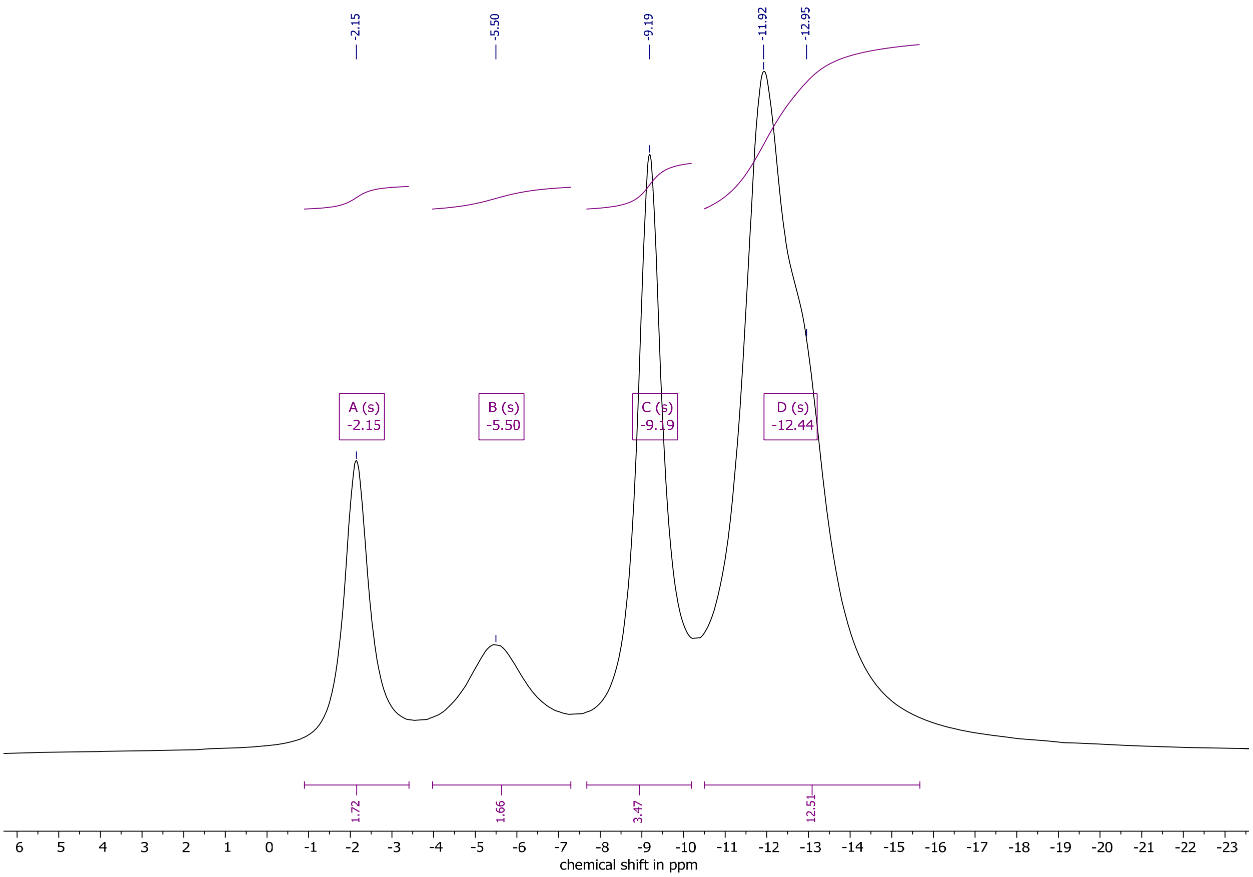

Figure S16. $^{11}B\{^{1}H\}$ NMR spectrum of compound **9** in $CDCl_3$.

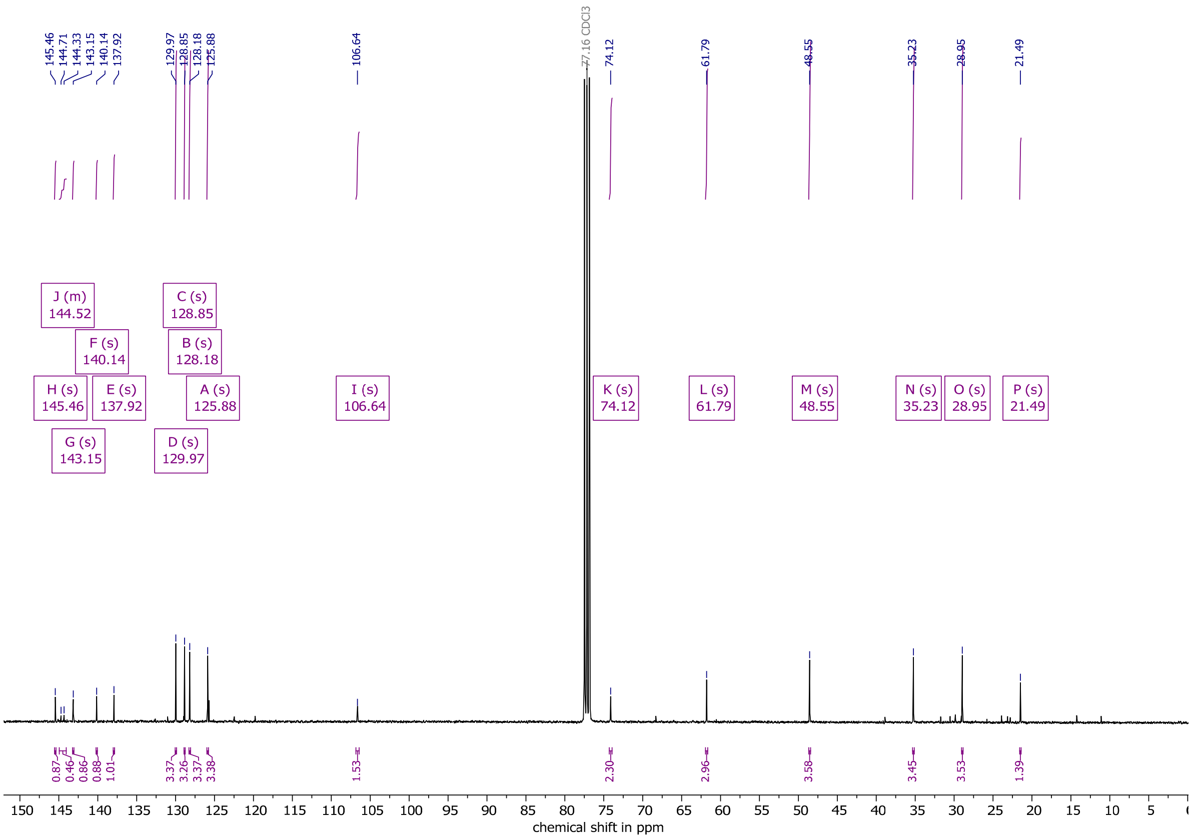

Figure S17. $^{13}C\{^{1}H\}$ NMR spectrum of compound **9** in $CDCl_3$.

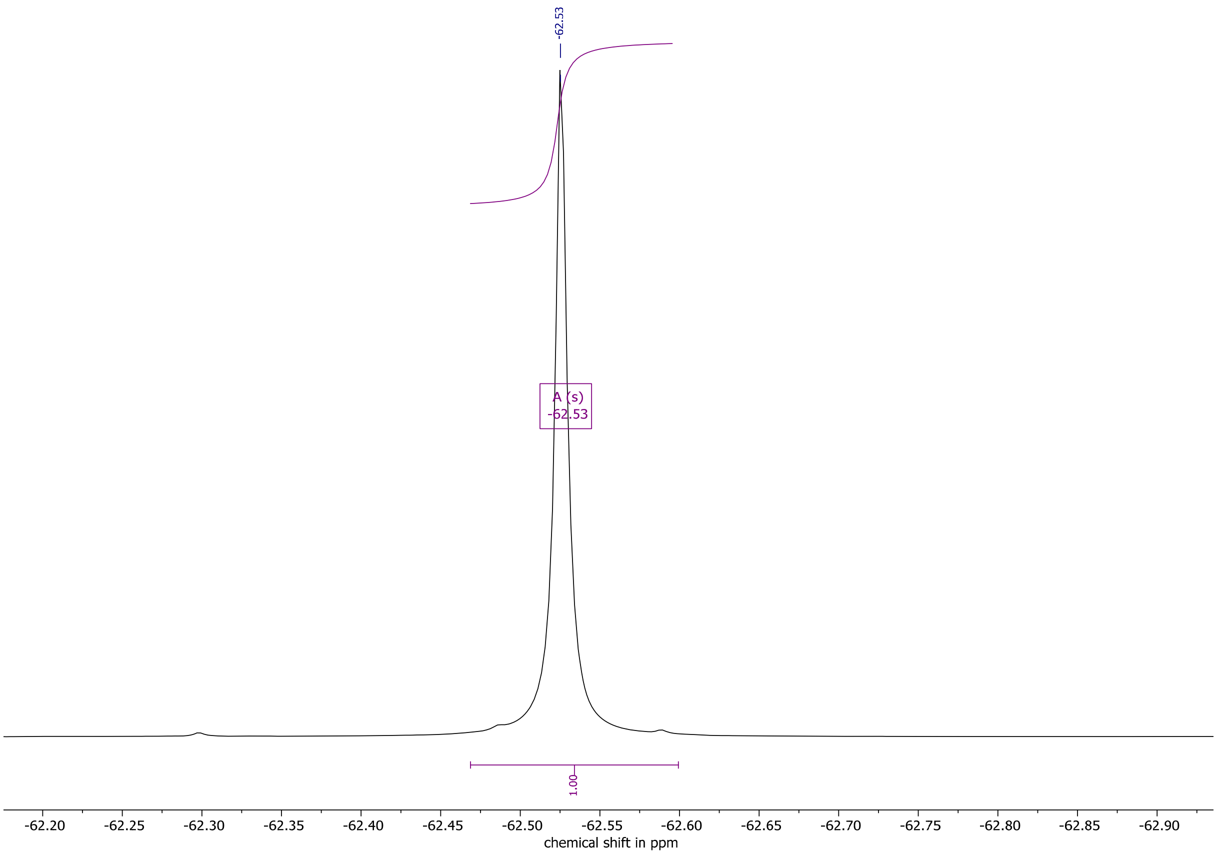

Figure S18. $^{19}F\{^{1}H\}$ NMR spectrum of compound **9** in $CDCl_3$.

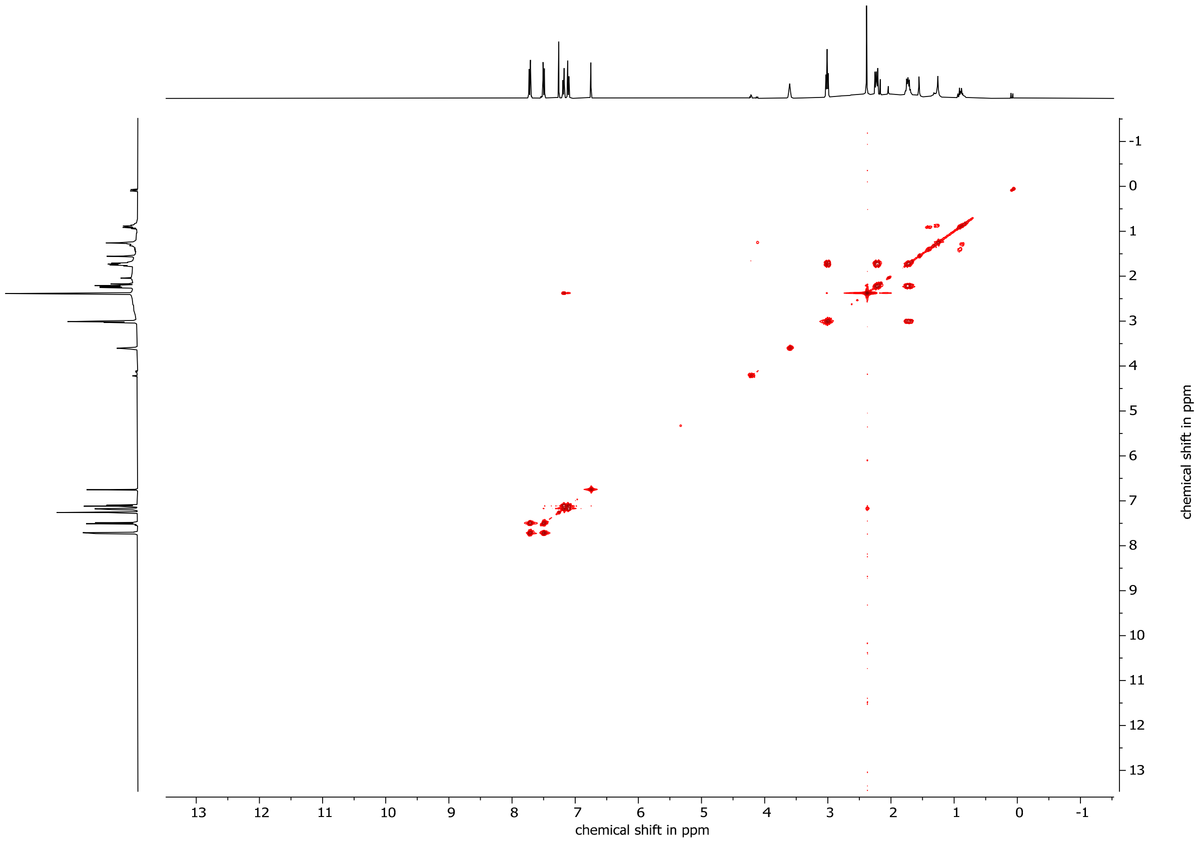

Figure S19. COSY (^{1}H, ^{1}H) NMR spectrum of compound **9** in $CDCl_3$.

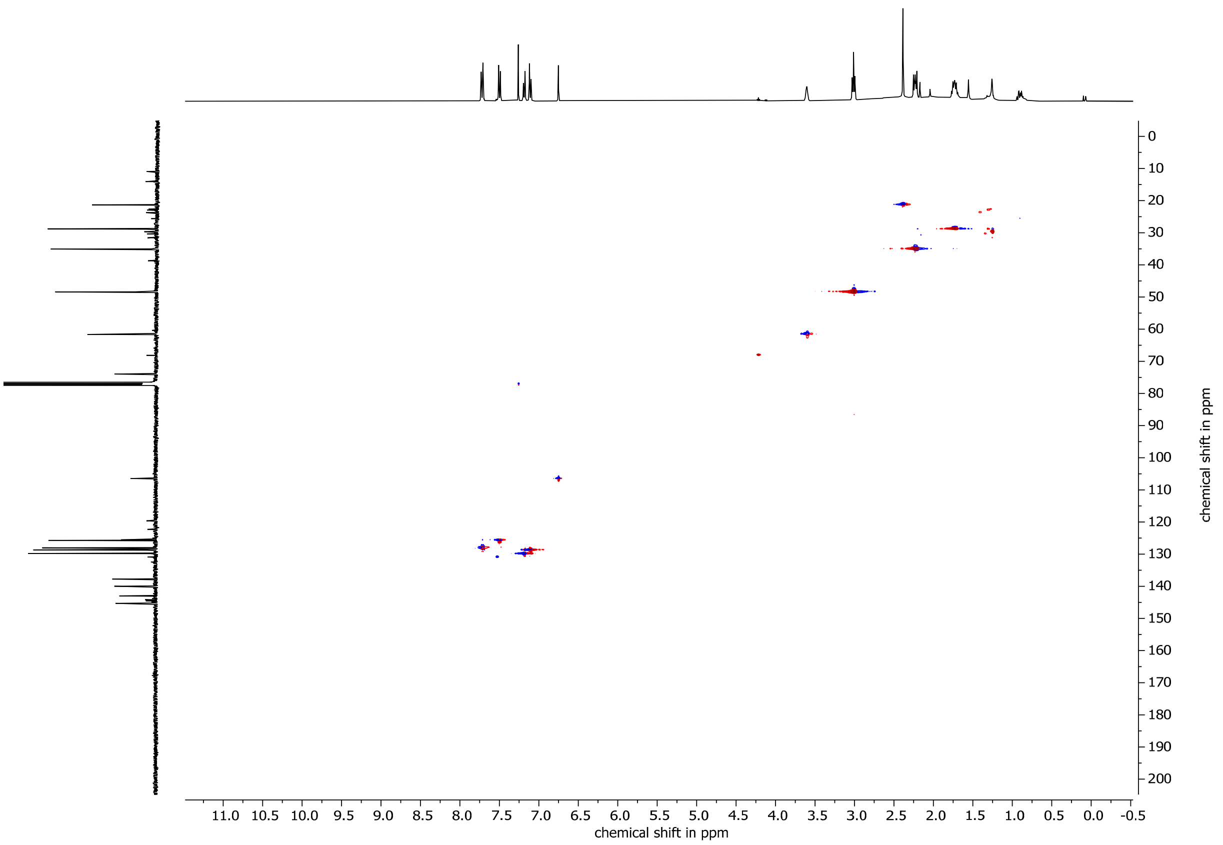

Figure S20. HSQC (^1H, ^{13}C) NMR spectrum of compound **9** in $CDCl_3$.

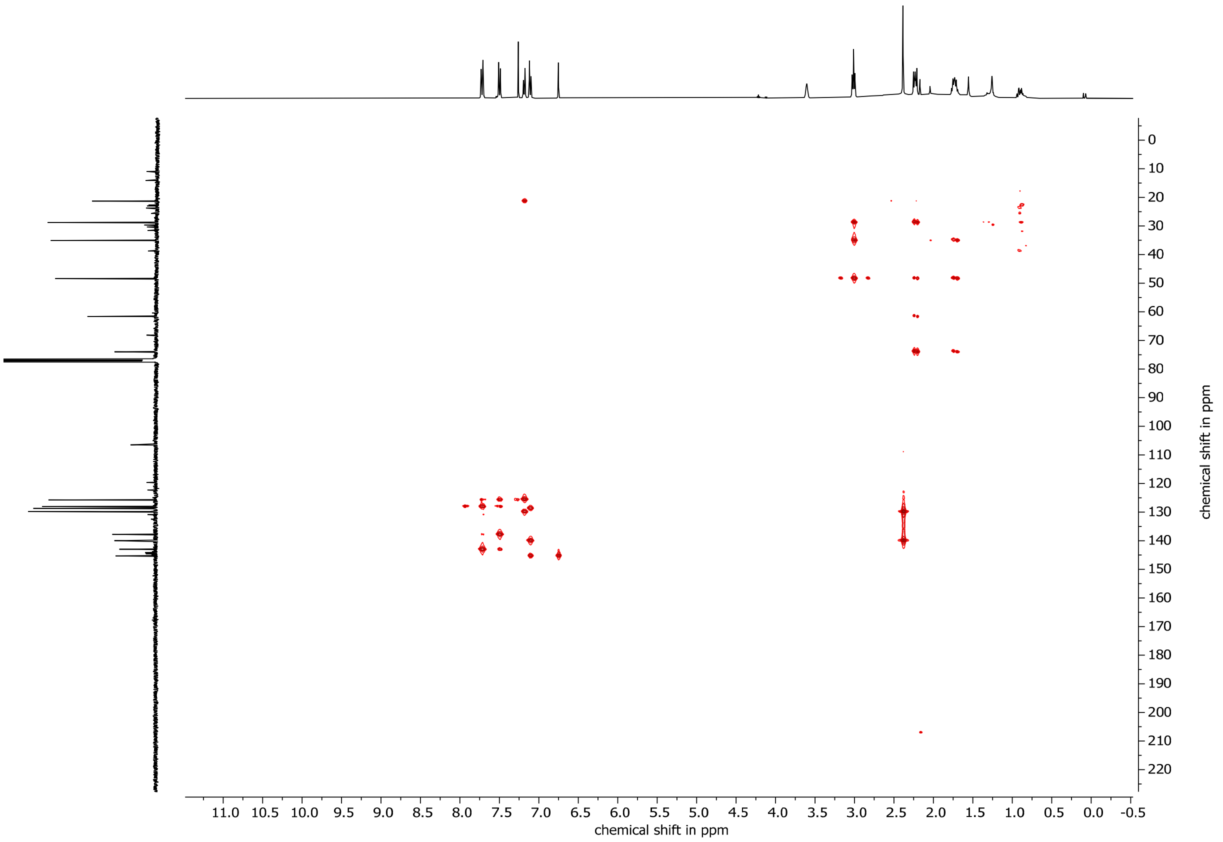

Figure S21. HMBC (^1H, ^{13}C) NMR spectrum of compound **9** in $CDCl_3$.

Compound 10

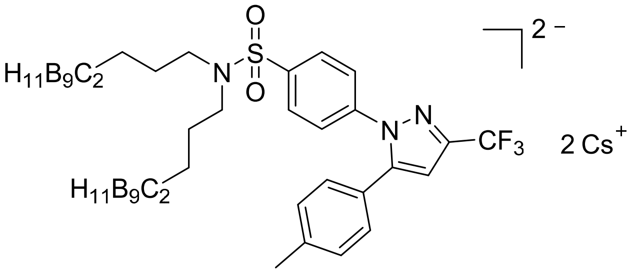

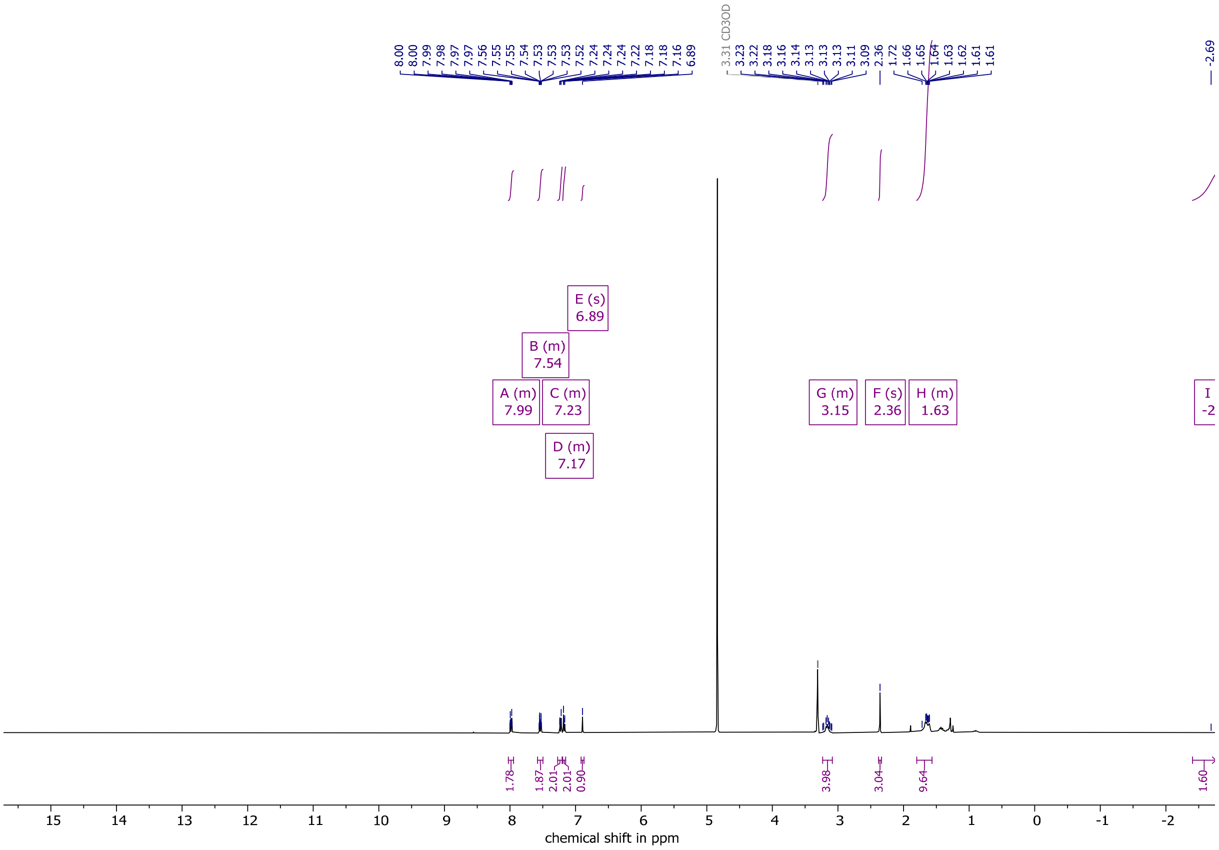

Figure S22. ^{1}H NMR spectrum (full spectrum) of compound **10** in CD_3OD.

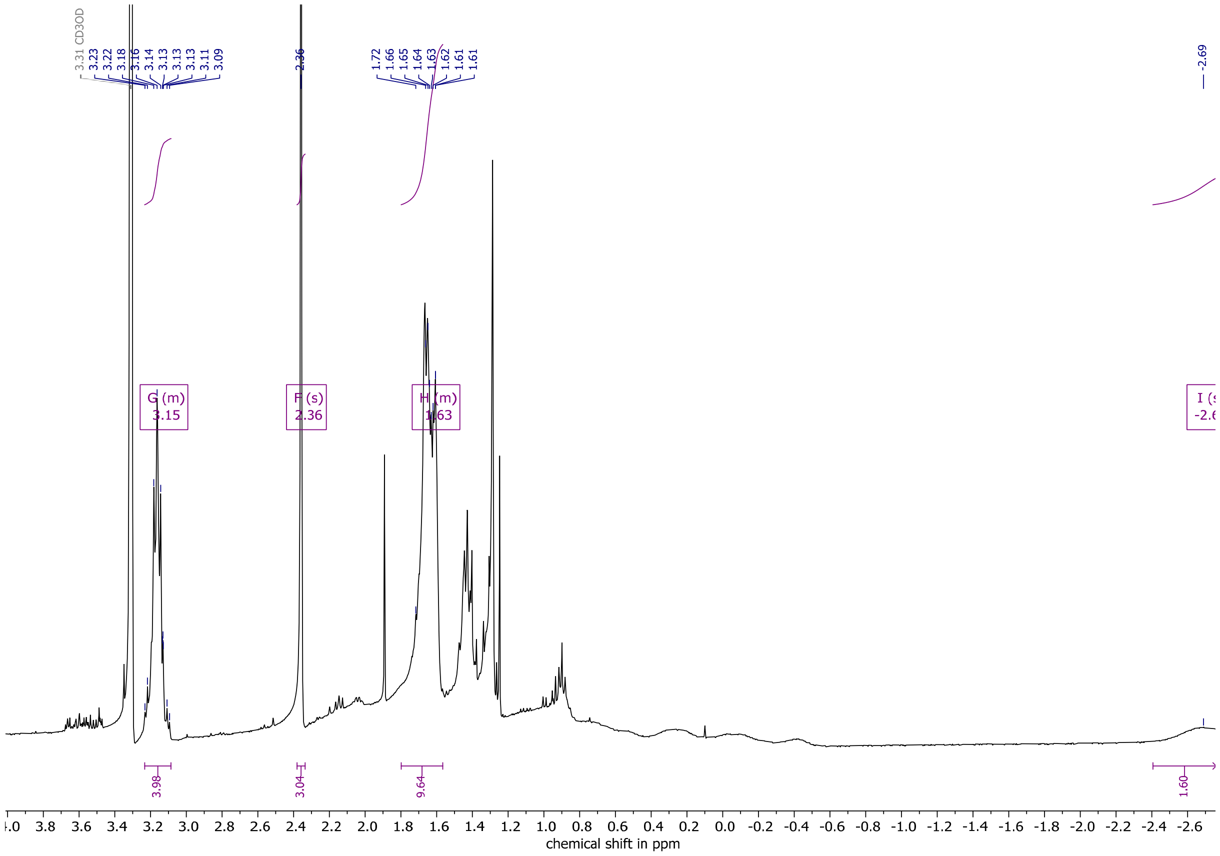

Figure S23. ^{1}H NMR spectrum (zoomed-in spectrum) of compound **10** in CD_3OD.

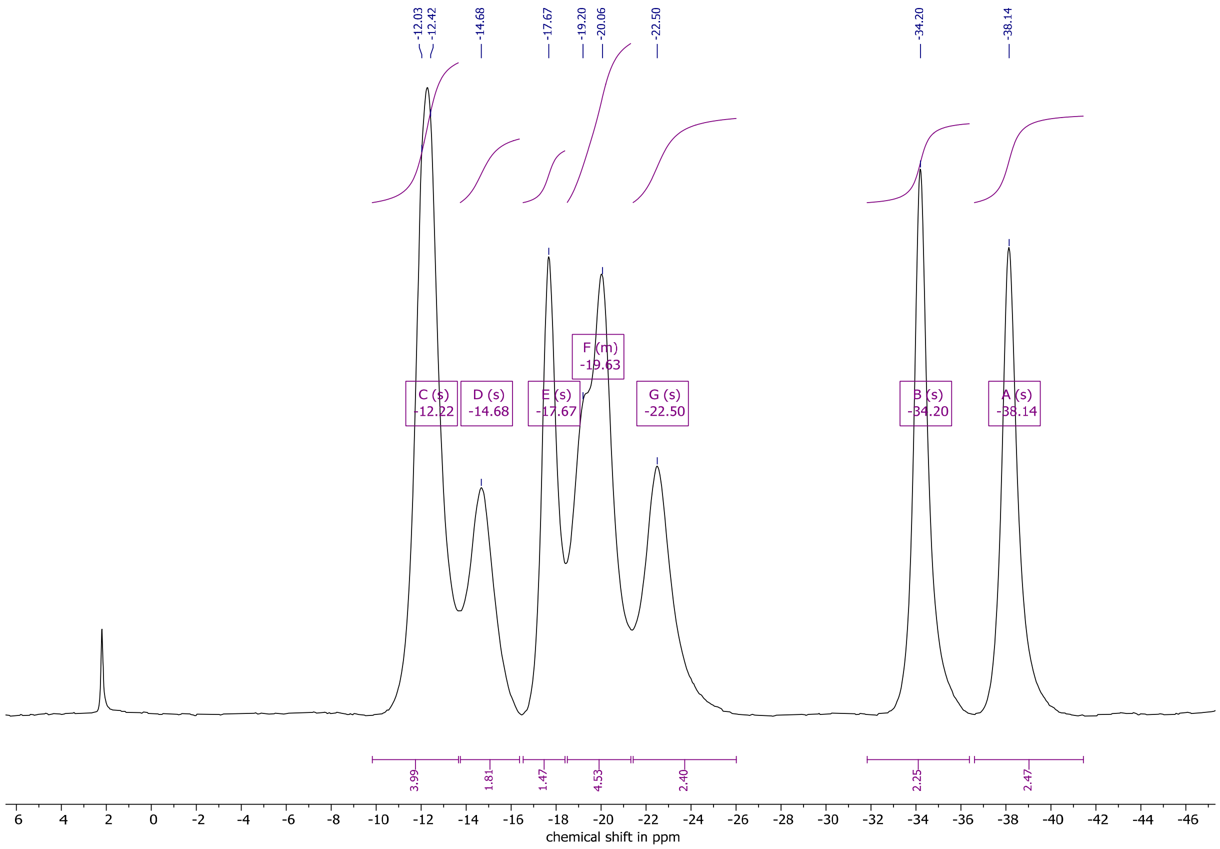

Figure S24. $^{11}B\{^{1}H\}$ NMR spectrum of compound **10** in CD_3OD.

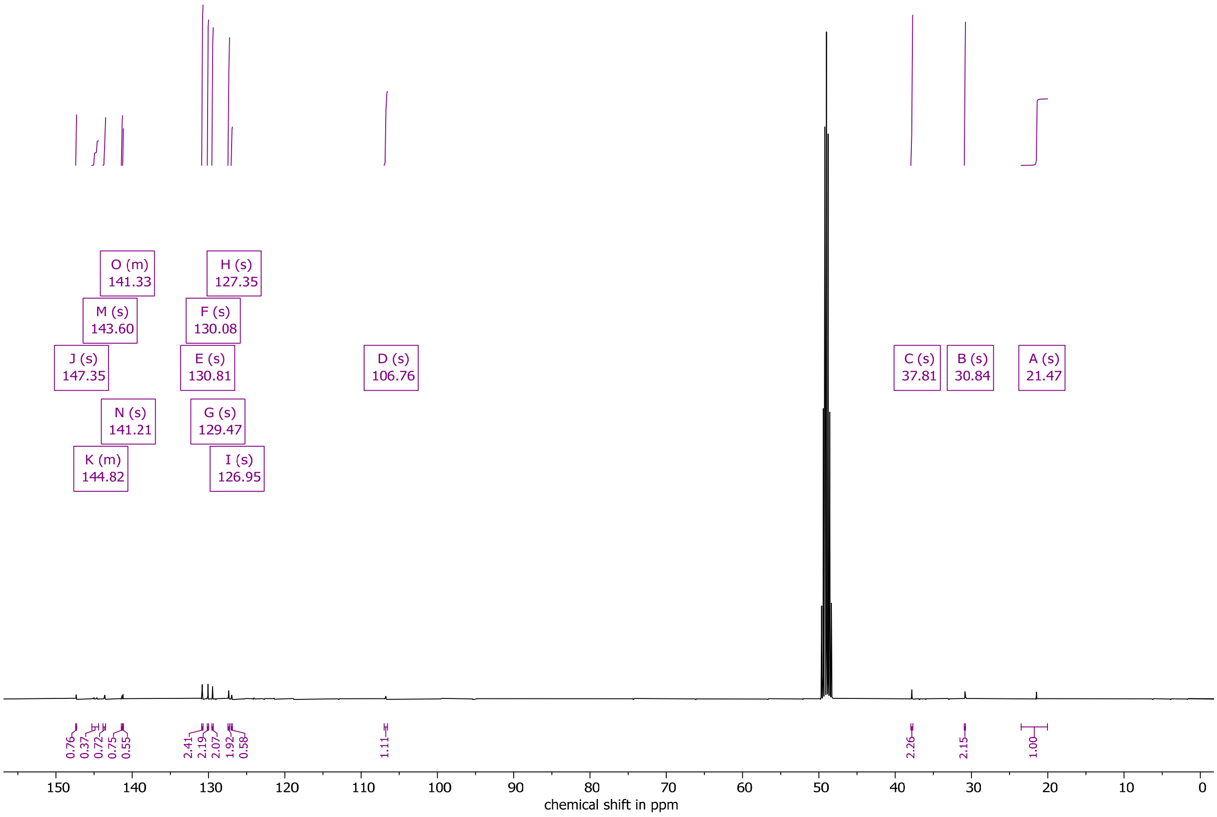

Figure S25. $^{13}C\{^{1}H\}$ NMR spectrum of compound **10** in CD_3OD.

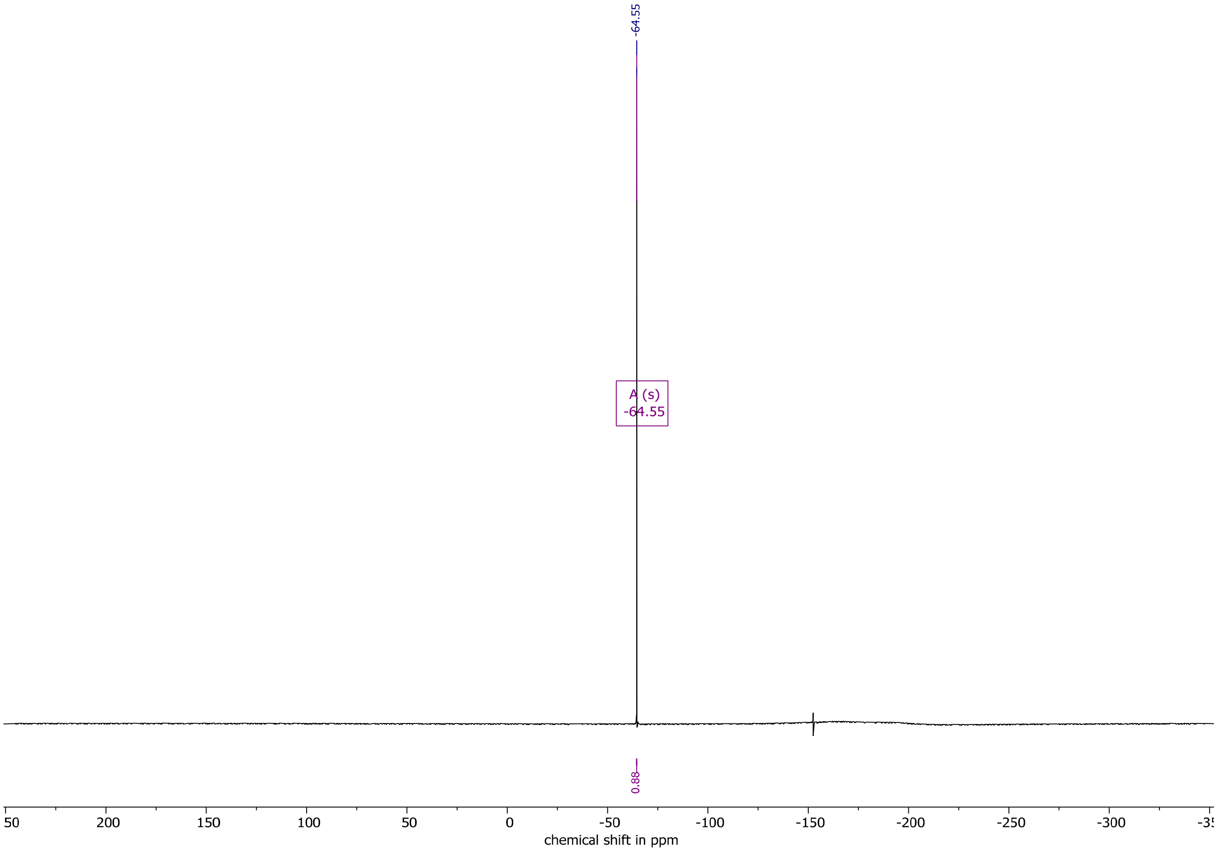

Figure S26. $^{19}F\{^{1}H\}$ NMR spectrum of compound **10** in CD_3OD.

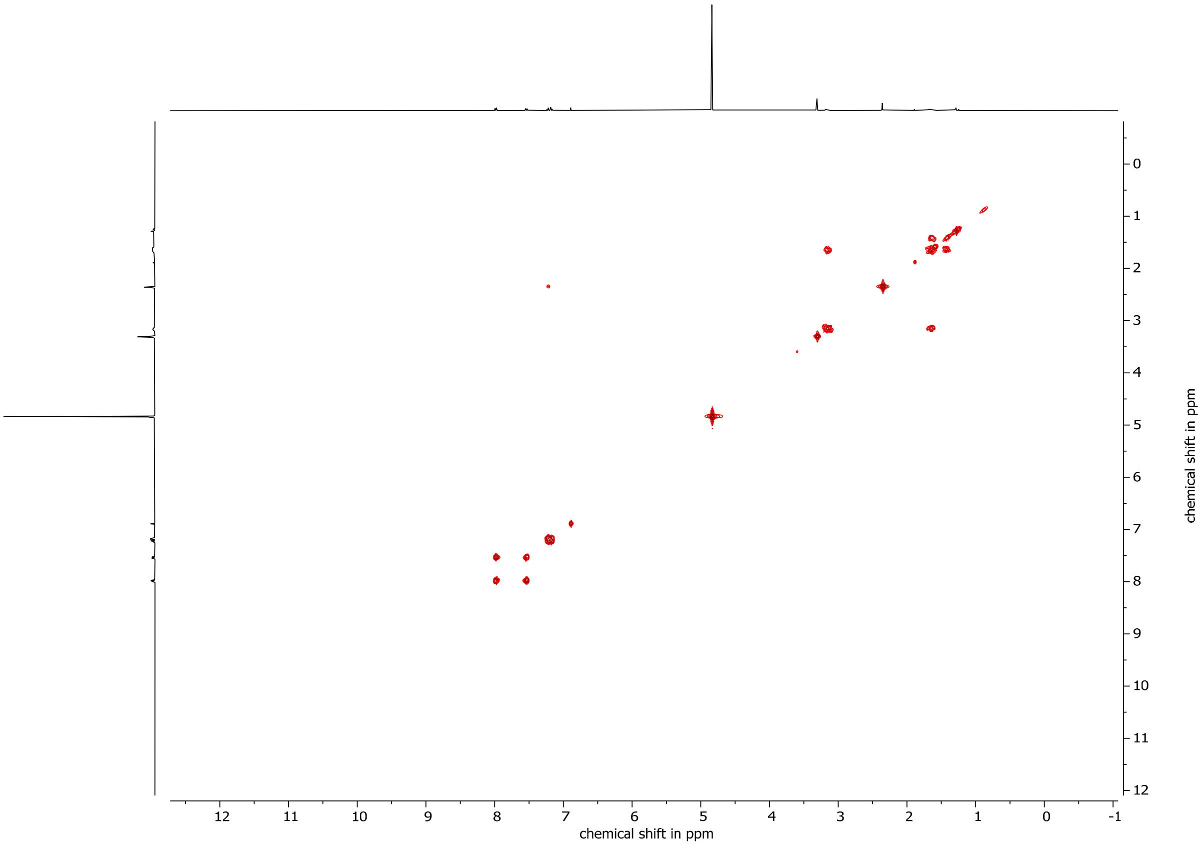

Figure S27. COSY (¹H, ¹H) NMR spectrum of compound **10** in CD_3OD.

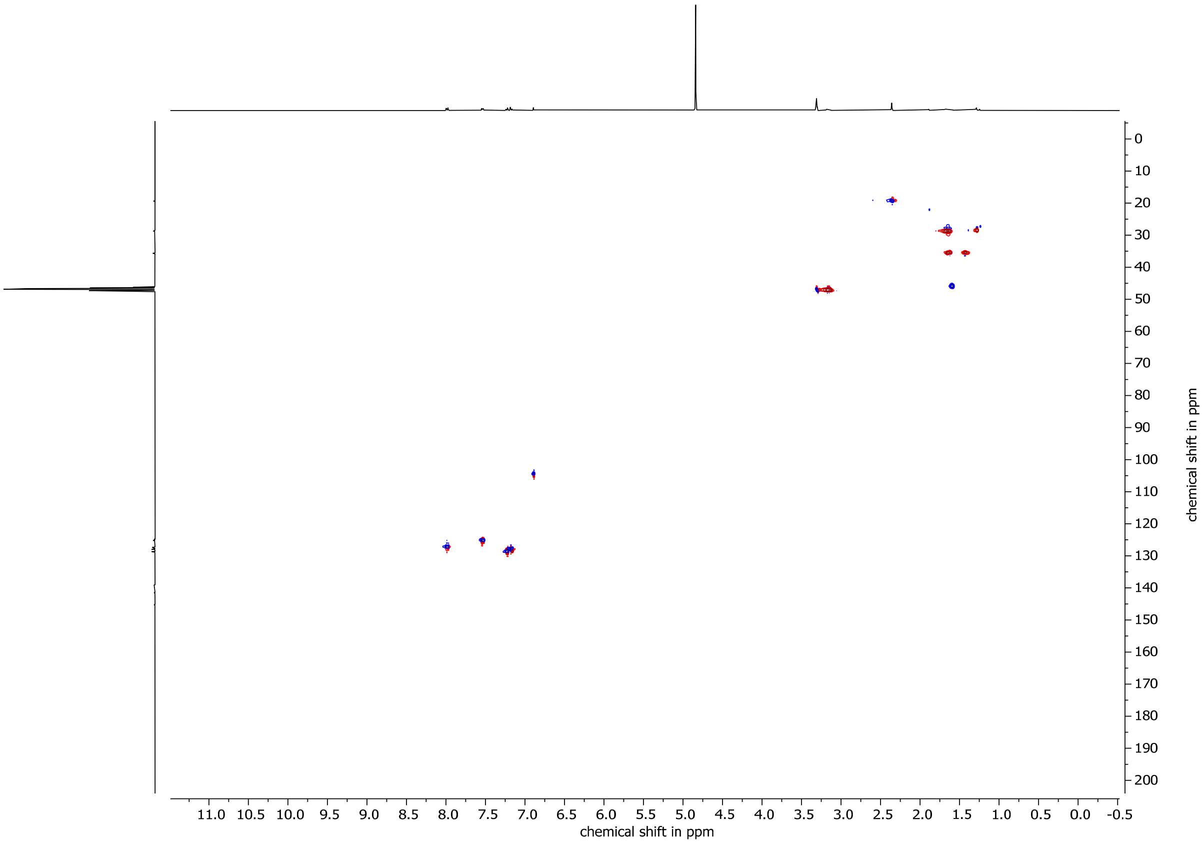

Figure S28. HSQC (¹H, ¹³C) NMR spectrum of compound **10** in CD_3OD.

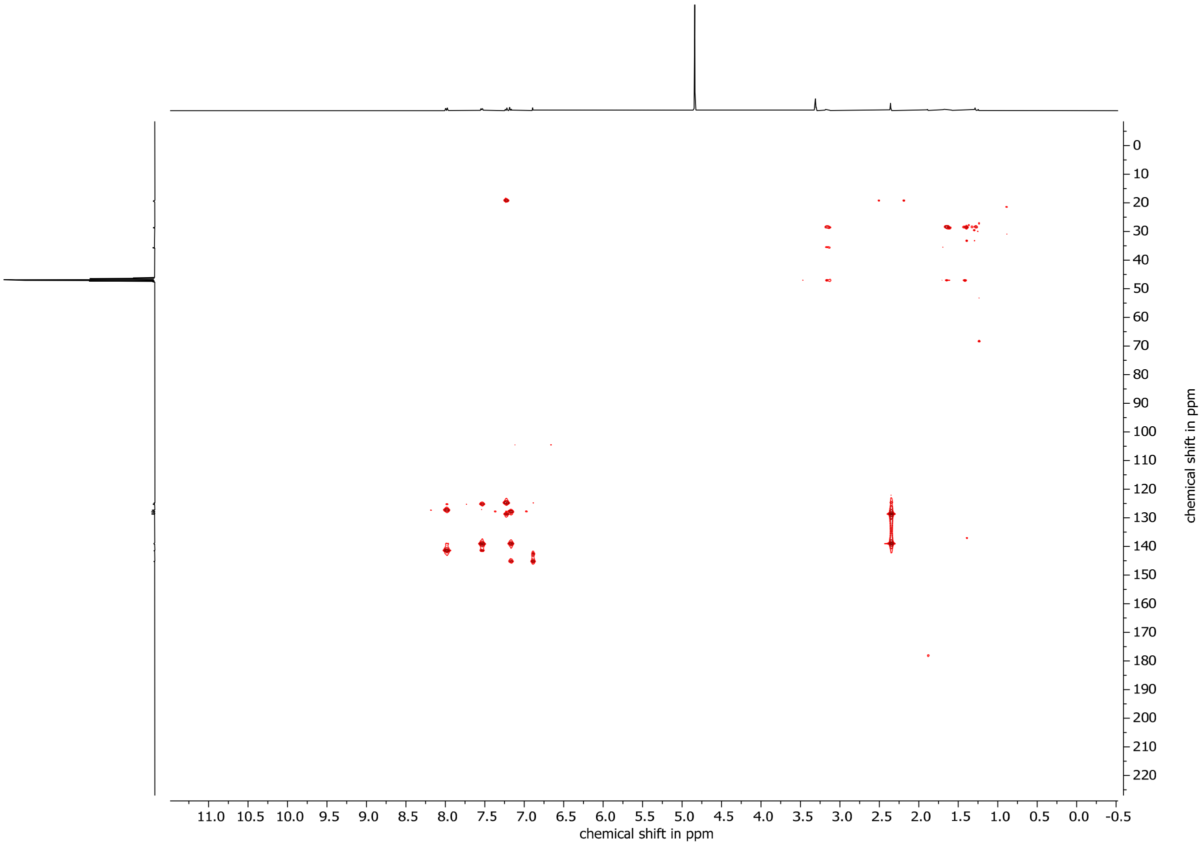

Figure S29. HMBC (^{1}H, ^{13}C) NMR spectrum of compound **10** in CD_3OD.

Compound 13a

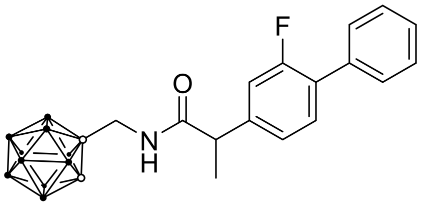

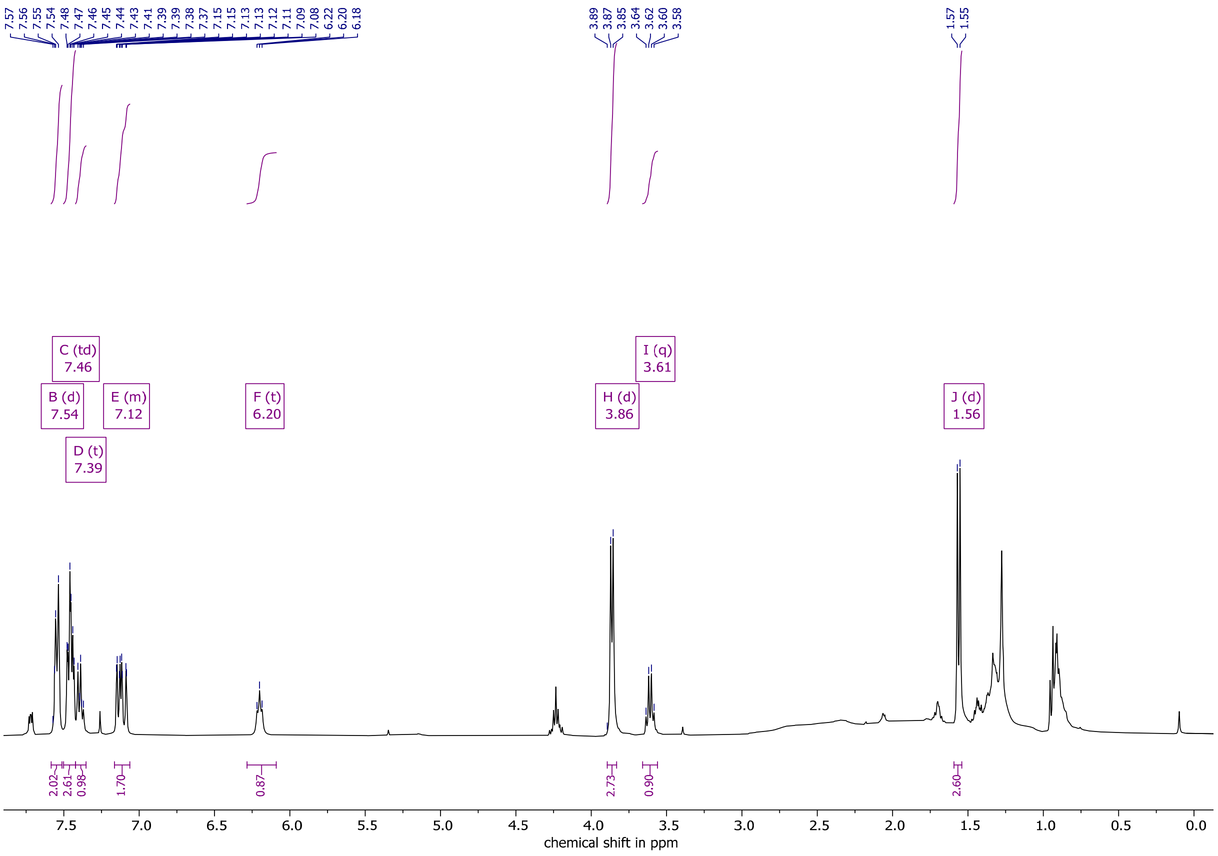

Figure S30. ^{1}H NMR spectrum of compound **13a** in $CDCl_3$.

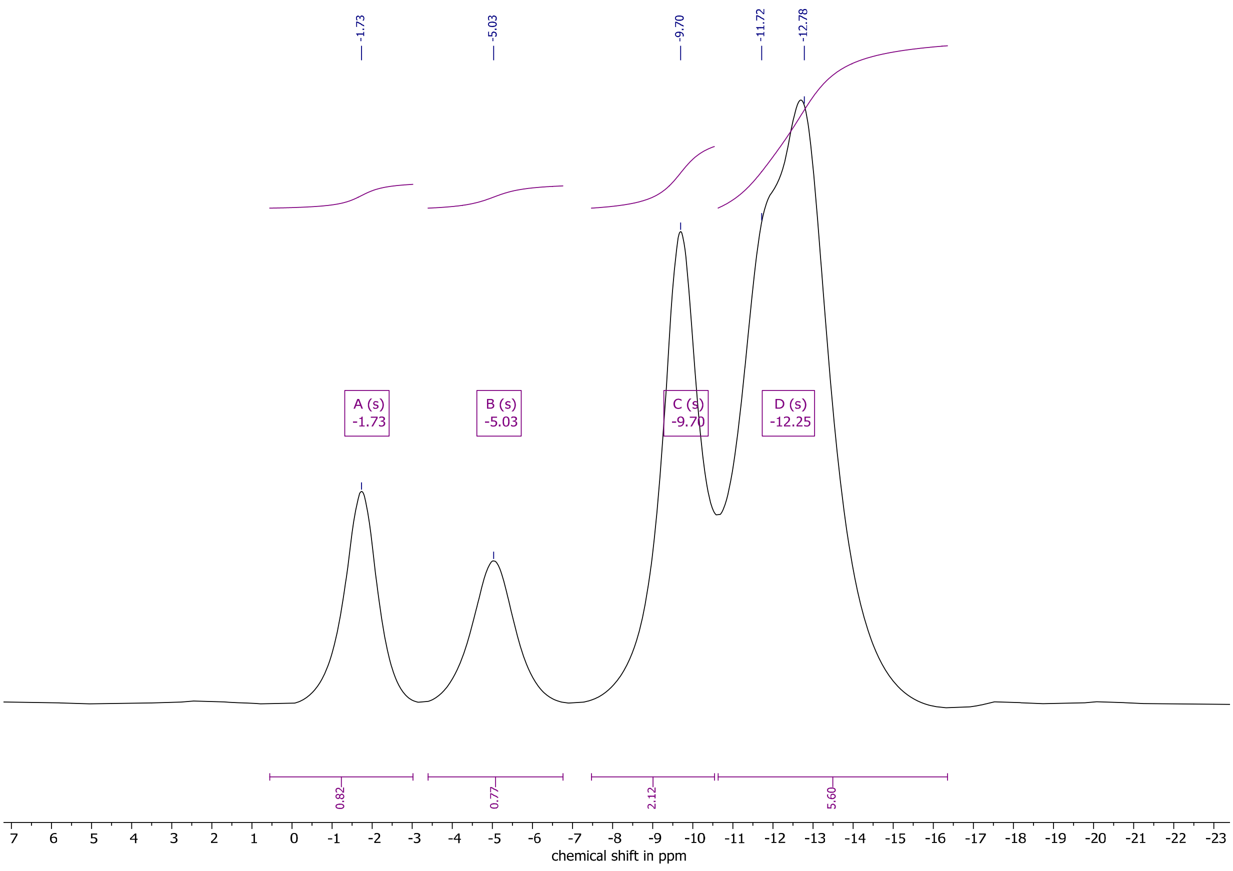

Figure S31. ^{11}B{^{1}H} NMR spectrum of compound **13a** in $CDCl_3$.

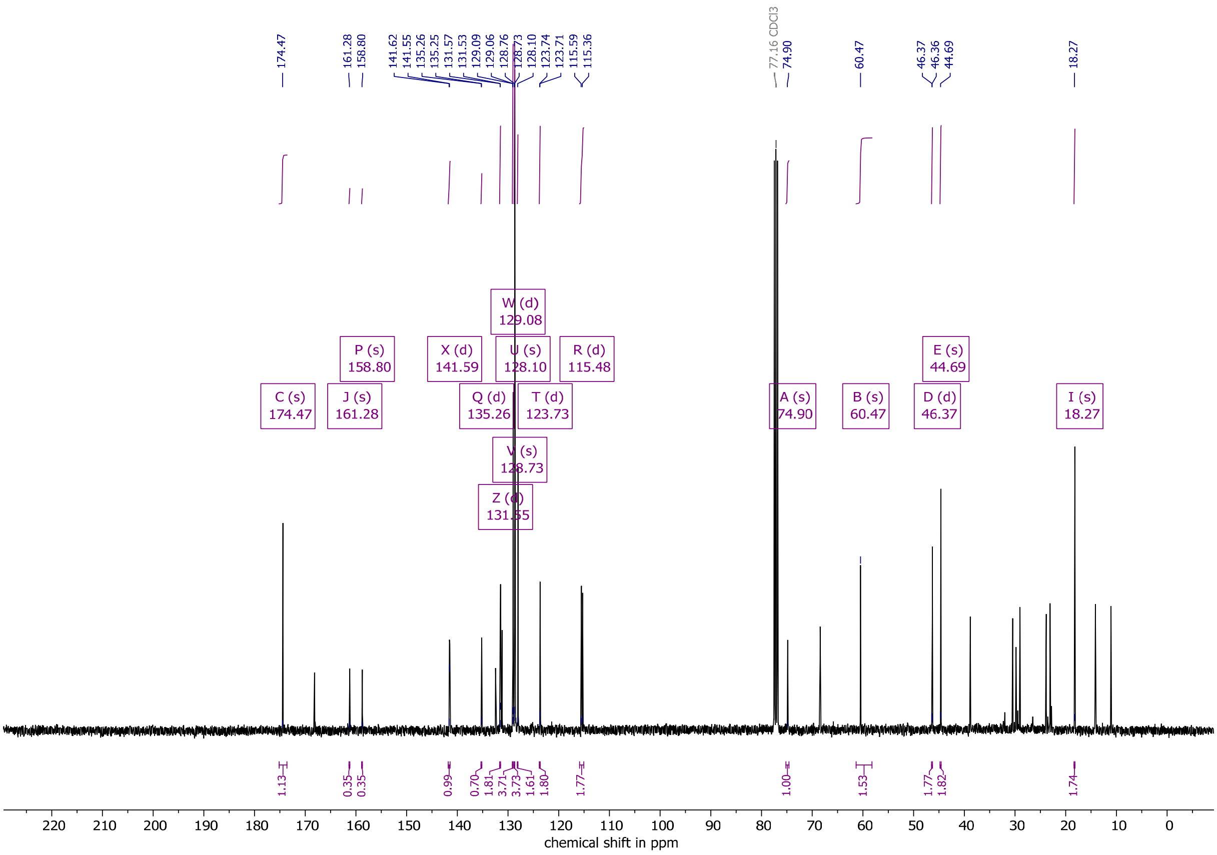

Figure S32. $^{13}C\{^{1}H\}$ NMR spectrum of compound **13a** in $CDCl_3$.

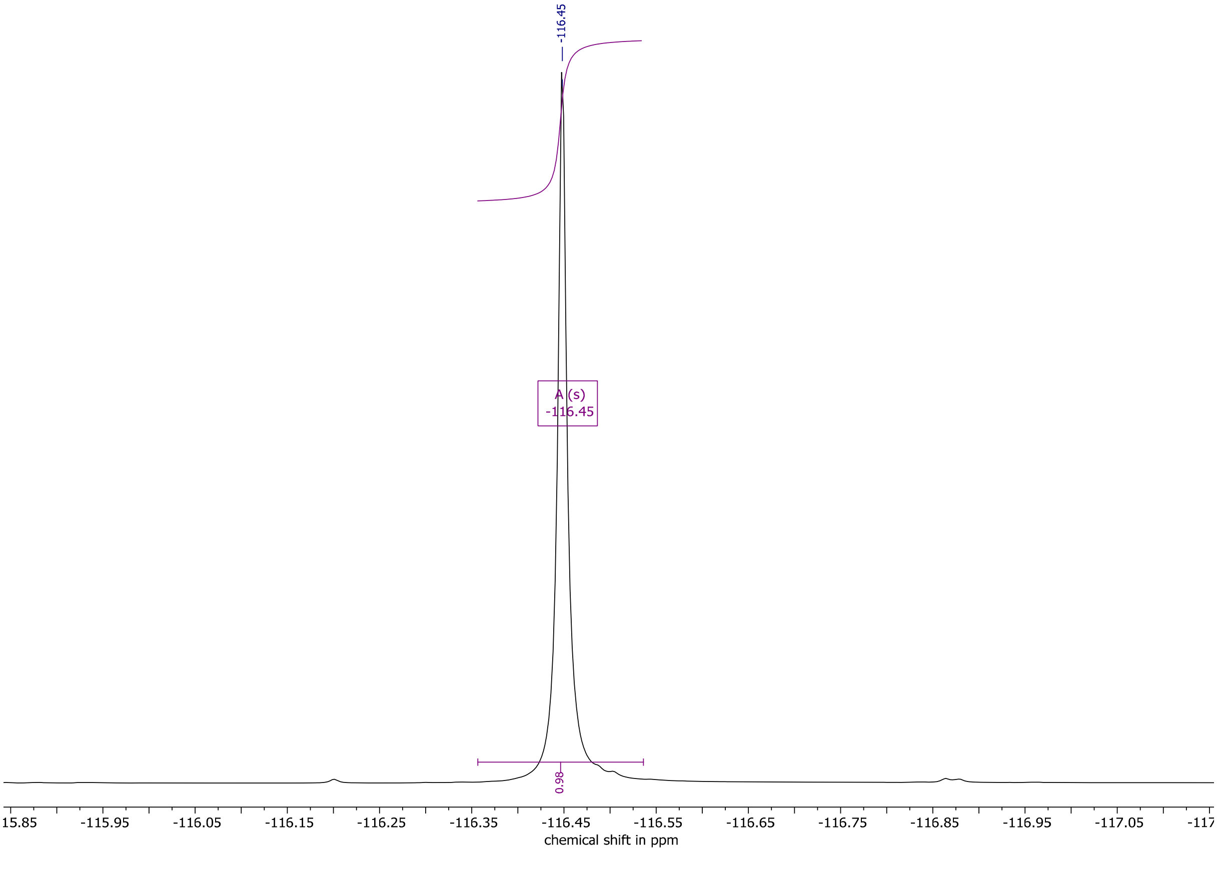

Figure S33. $^{19}F\{^{1}H\}$ NMR spectrum of compound **13a** in $CDCl_3$.

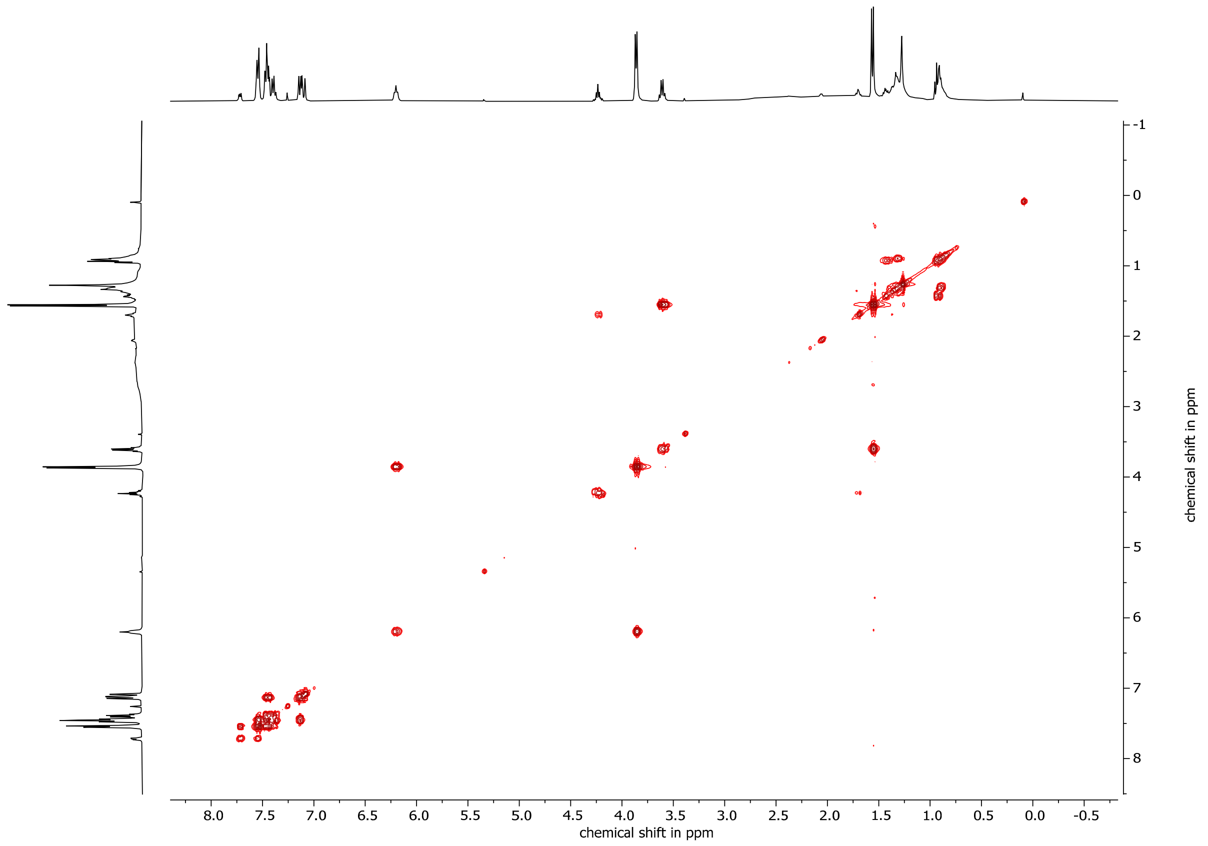

Figure S34. COSY (^{1}H, ^{1}H) NMR spectrum of compound **13a** in $CDCl_3$.

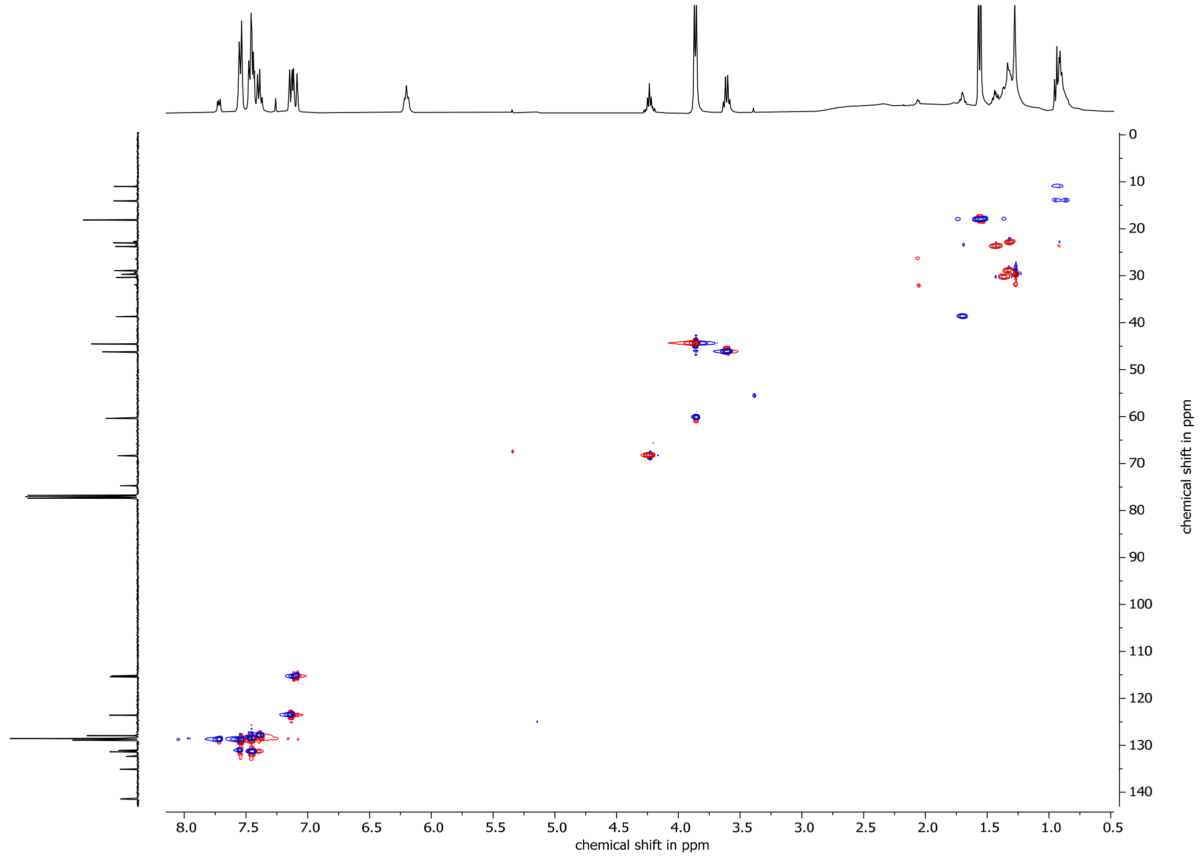

Figure S35. HSQC (^{1}H, ^{13}C) NMR spectrum of compound **13a** in $CDCl_3$.

Compound 14a

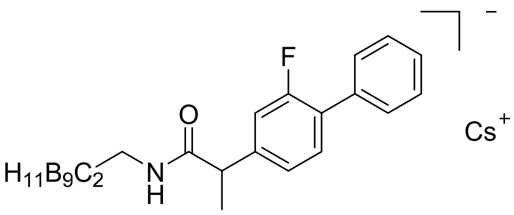

The $^{11}B\{^1H\}$ and $^{19}F\{^1H\}$ NMR spectra have been measured before final purification with semipreparative column chromatography. The ^1H NMR spectrum was measured afterwards.

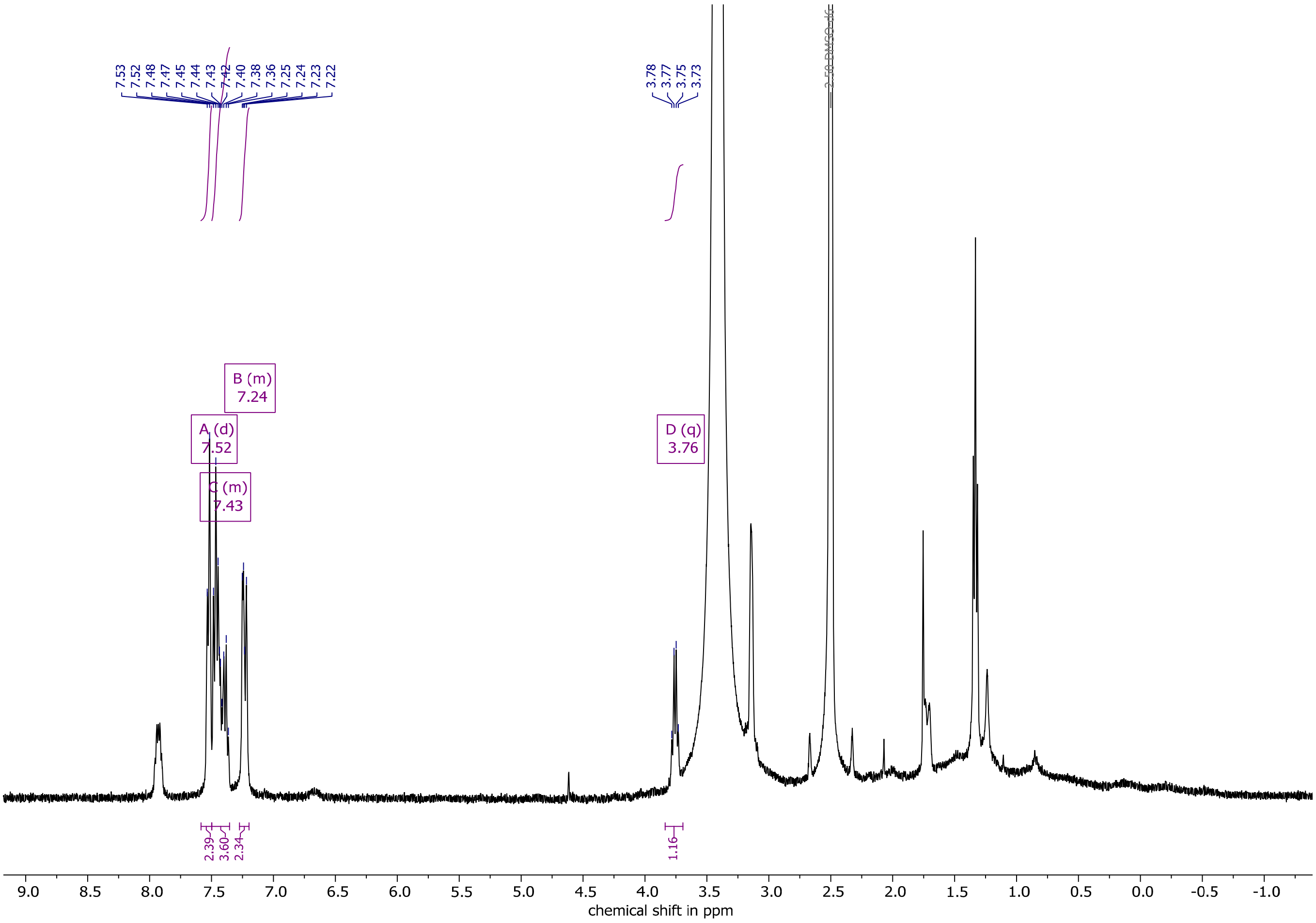

Figure S36. ^1H NMR spectrum of compound **14a** in $(CD_3)_2SO$.

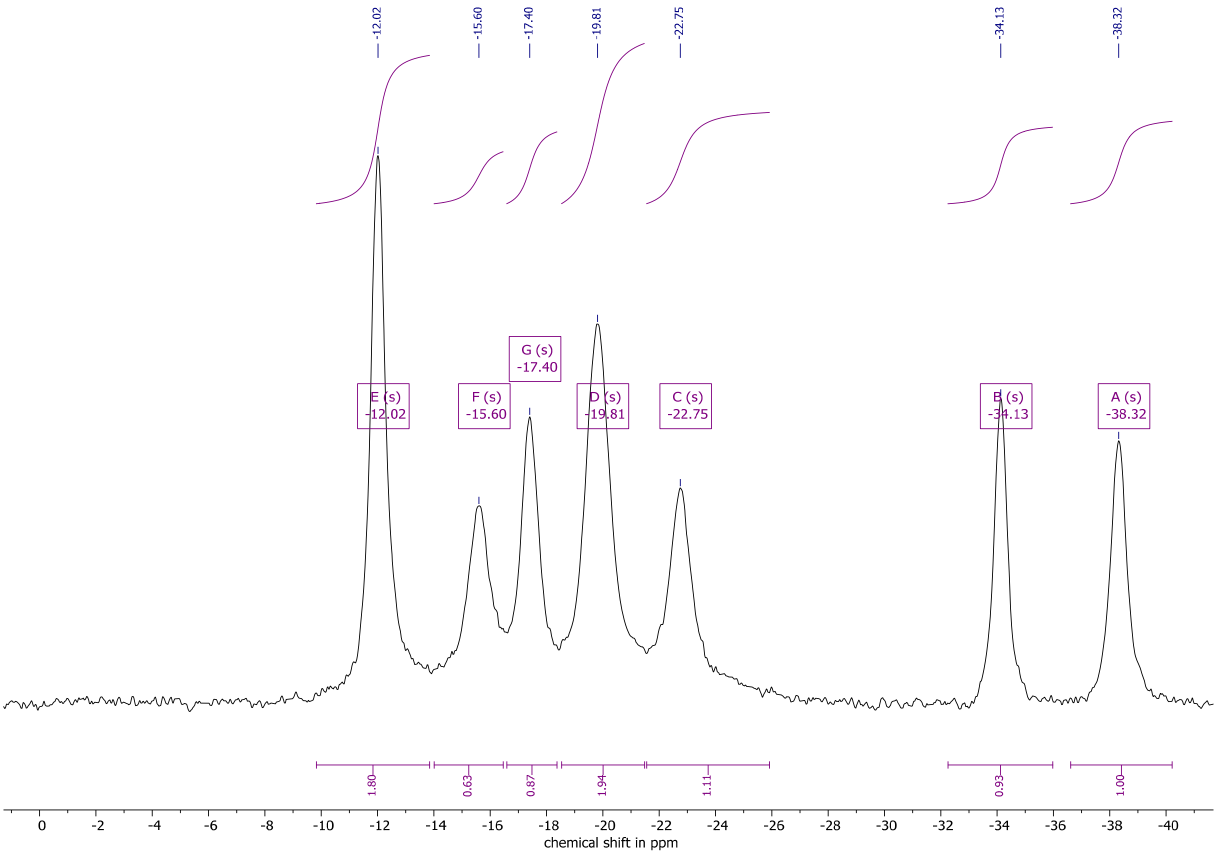

Figure S37. $^{11}B\{^1H\}$ NMR spectrum of compound **14a** in CD_3OD.

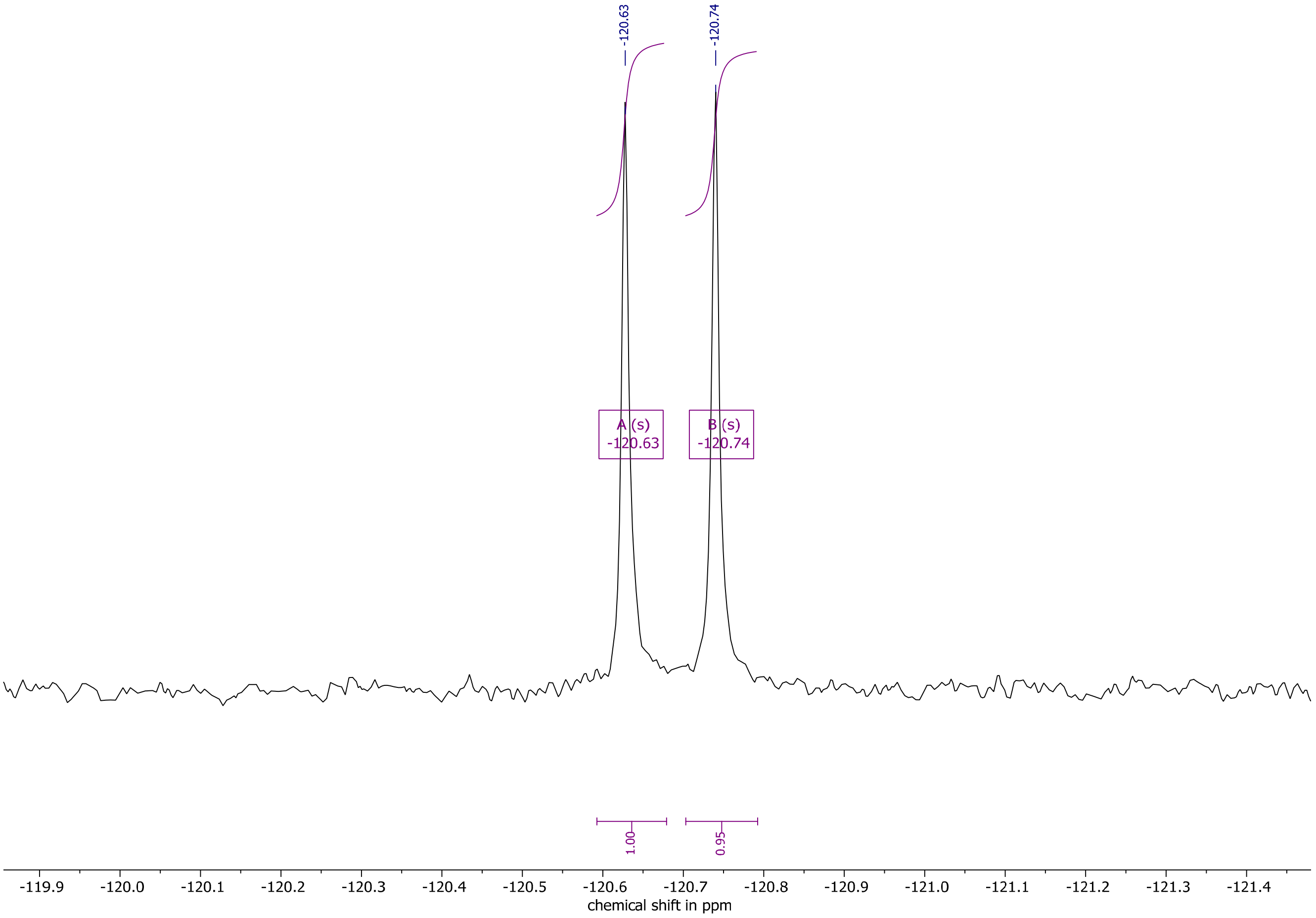

Figure S38. $^{19}F\{^1H\}$ NMR spectrum of compound **14a** in CD_3OD.

Compound 13b

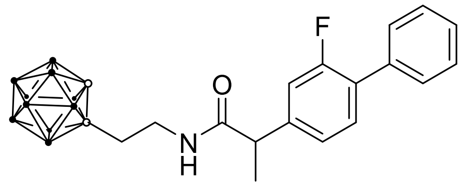

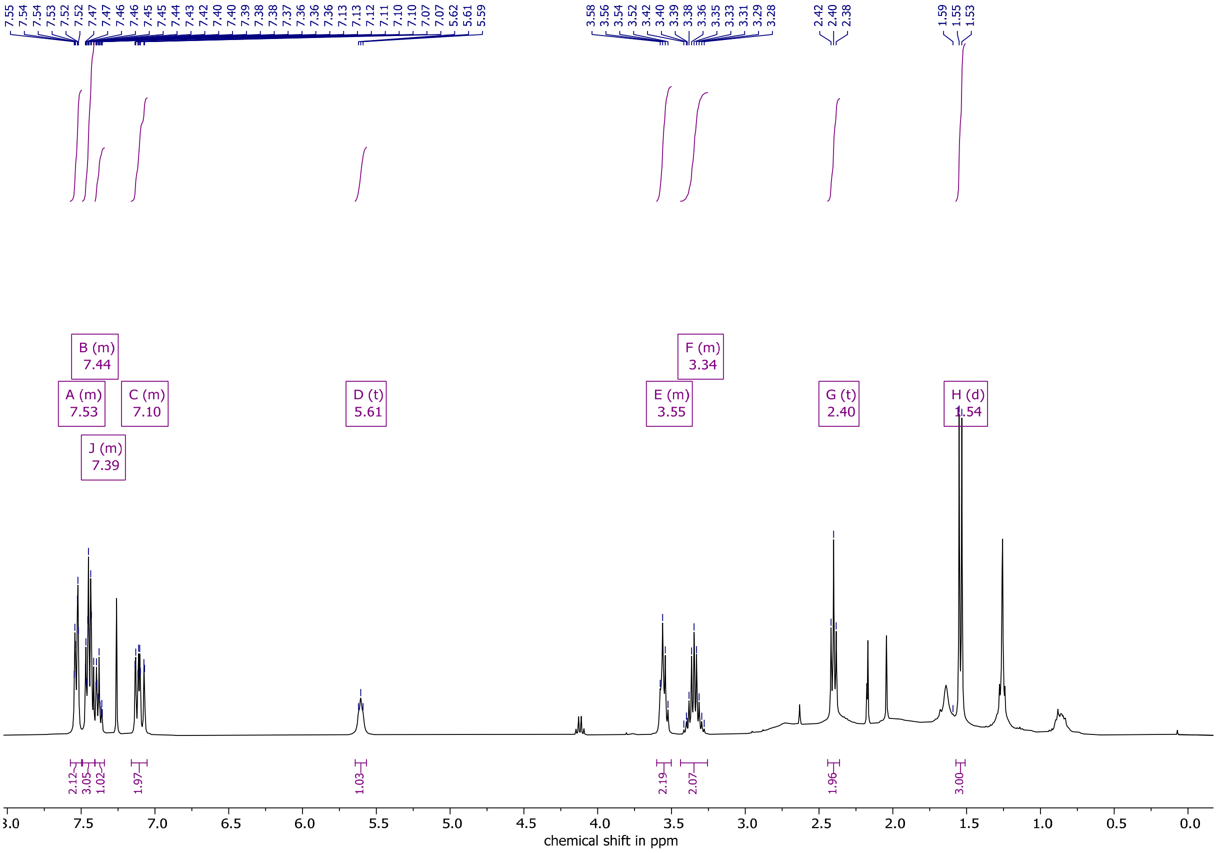

Figure S39. ^{1}H NMR spectrum of compound **13b** in $CDCl_3$.

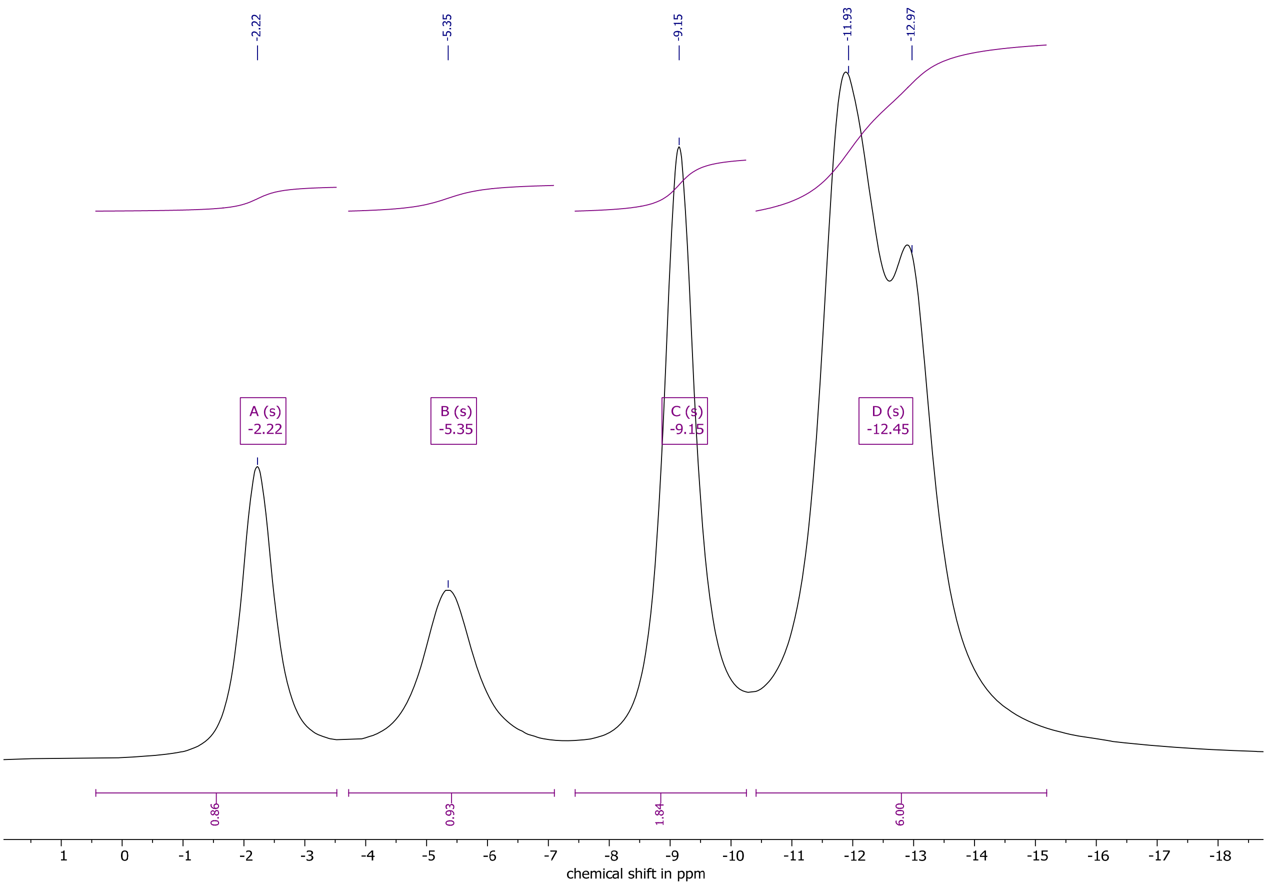

Figure S40. $^{11}B\{^1H\}$ NMR spectrum of compound **13b** in $CDCl_3$.

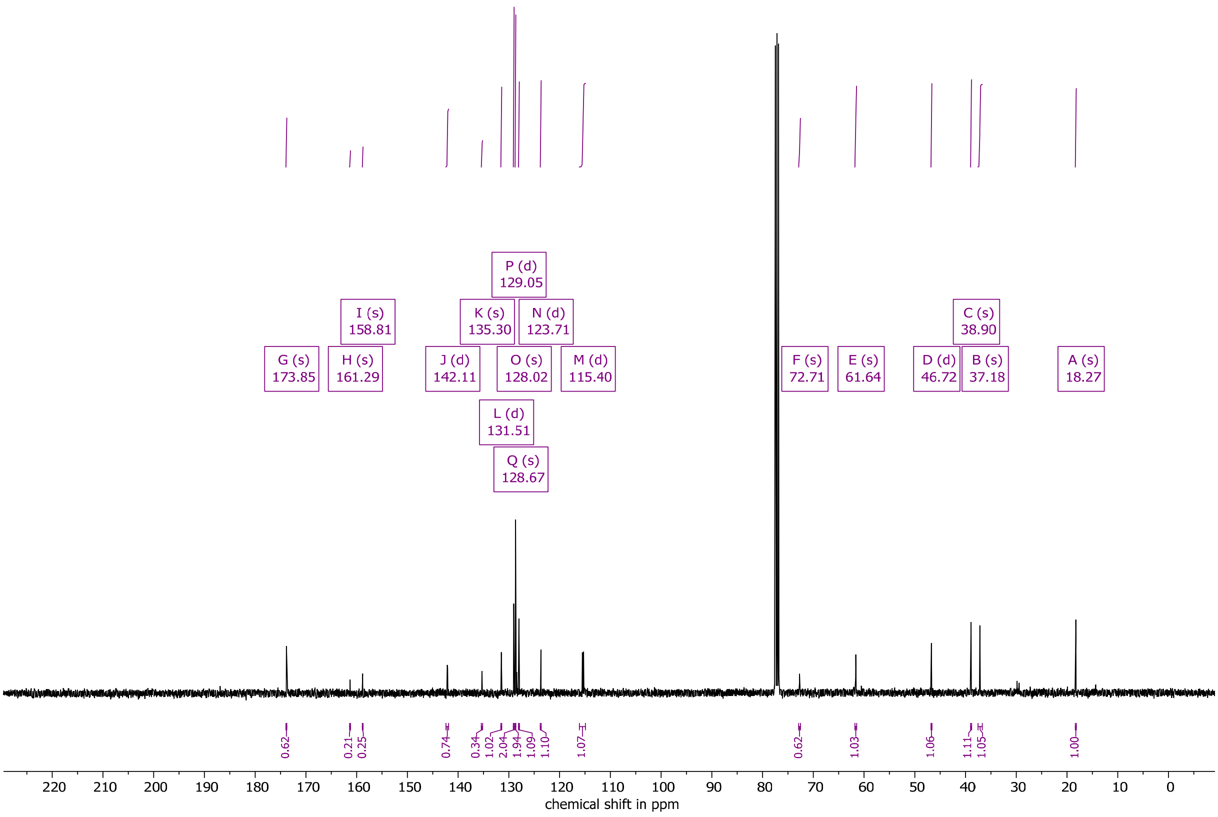

Figure S41. $^{13}C\{^1H\}$ NMR spectrum of compound **13b** in $CDCl_3$.

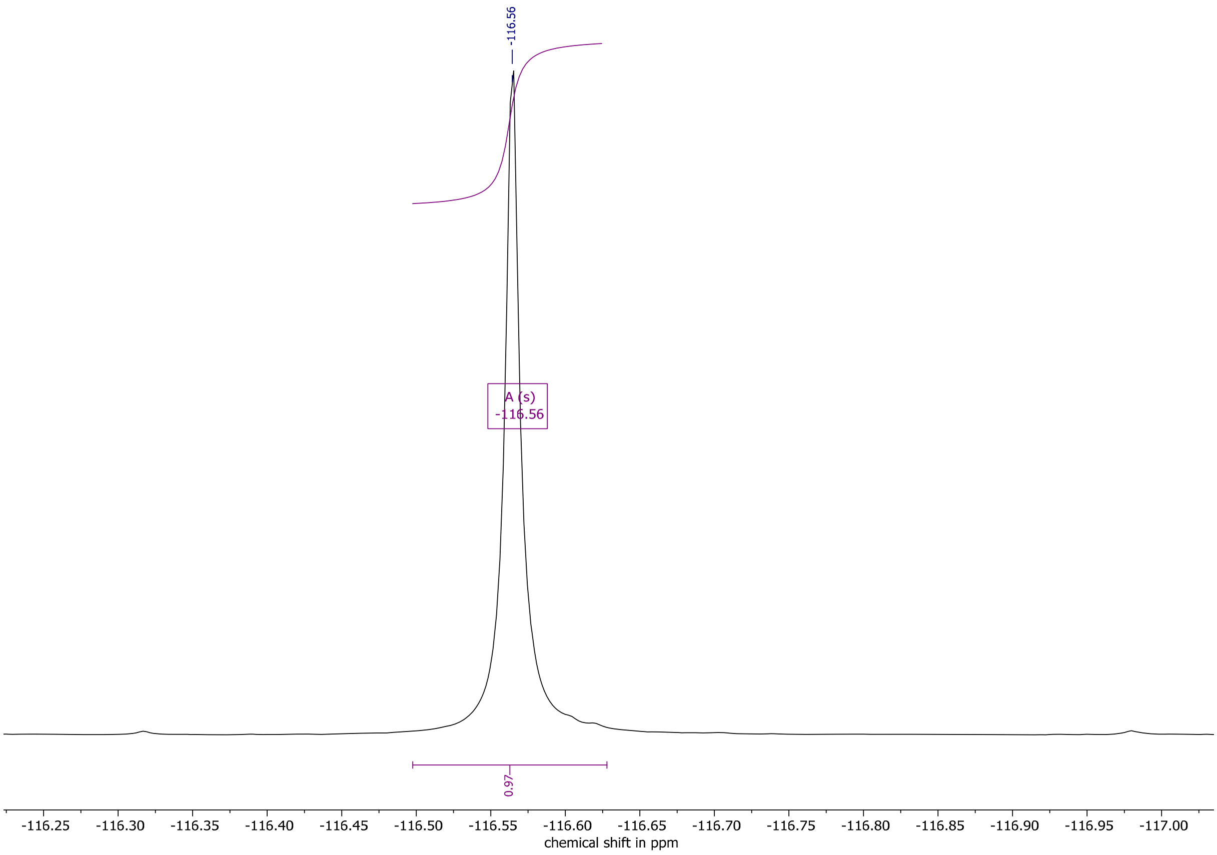

Figure S42. $^{19}F\{^{1}H\}$ NMR spectrum of compound **13b** in $CDCl_3$.

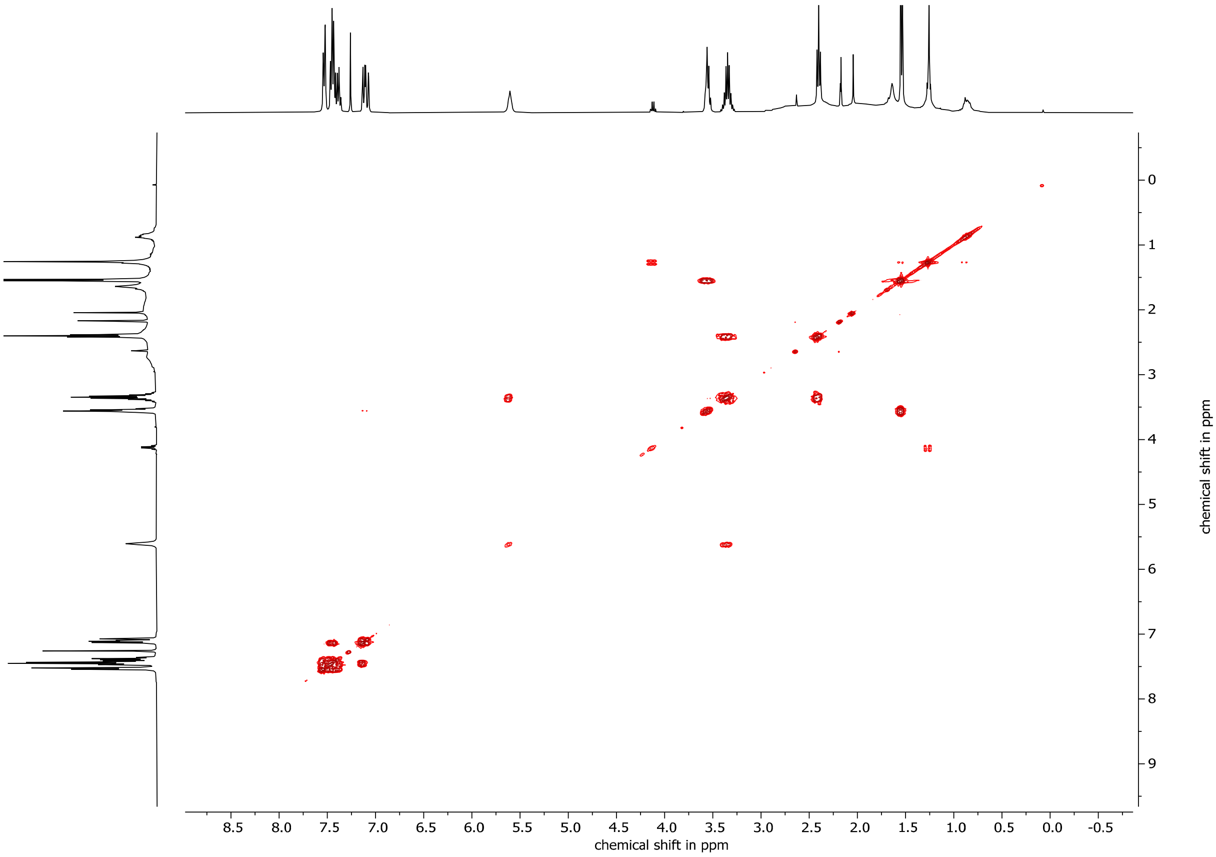

Figure S43. COSY (^{1}H, ^{1}H) NMR spectrum of compound **13b** in $CDCl_3$.

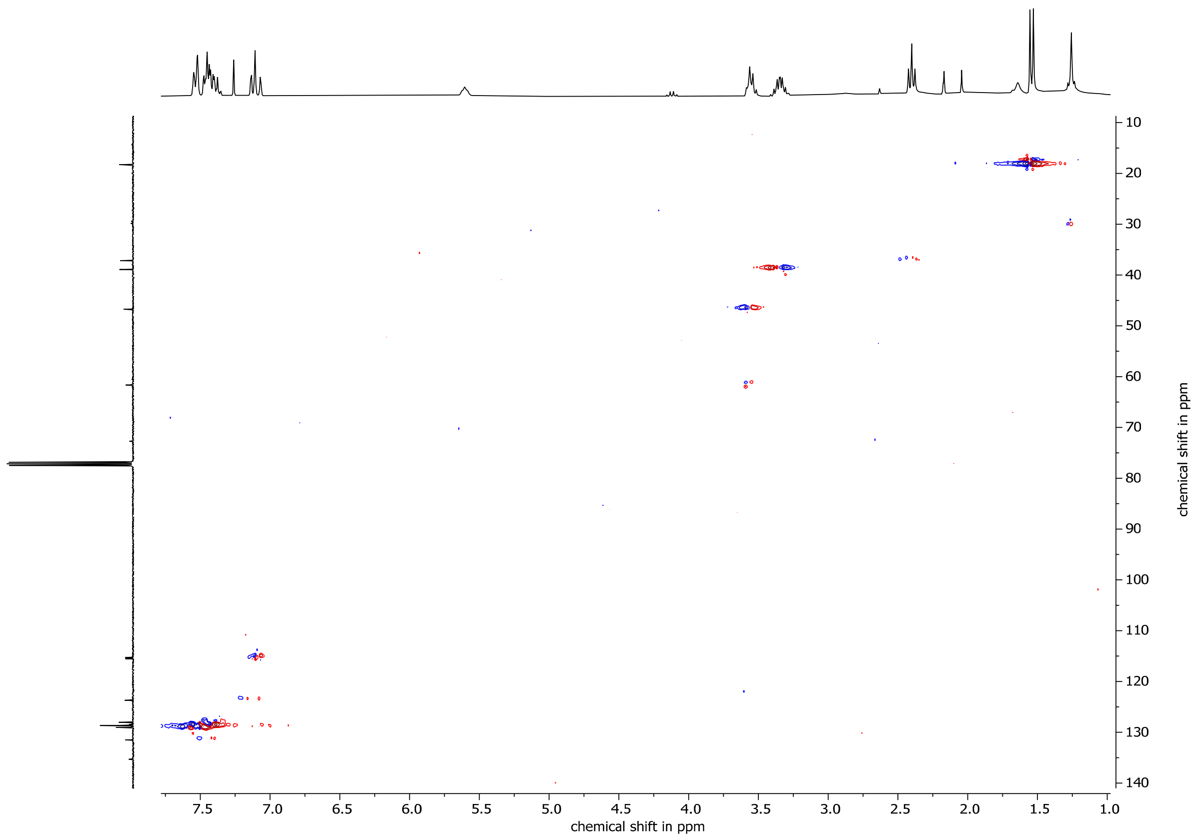

Figure S44. HSQC (1H, 13C) NMR spectrum of compound **13b** in CDCl3.

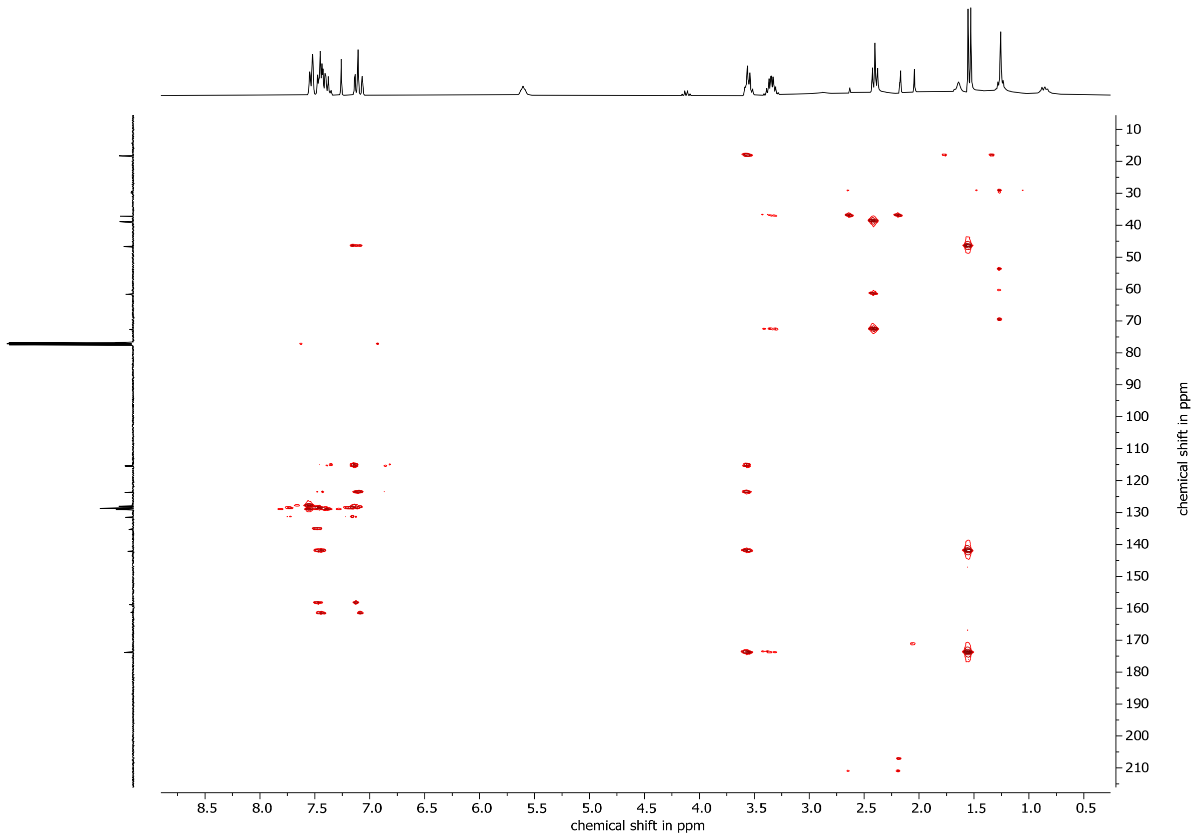

Figure S45. HMBC (1H, 13C) NMR spectrum of compound **13b** in CDCl3.

Compound 14b

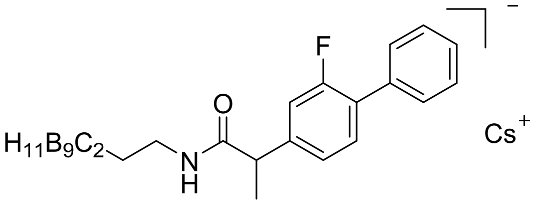

The $^{11}B\{^1H\}$ and $^{19}F\{^1H\}$ NMR spectra have been measured before final purification with semipreparative column chromatography. The ^1H NMR spectrum was measured afterwards.

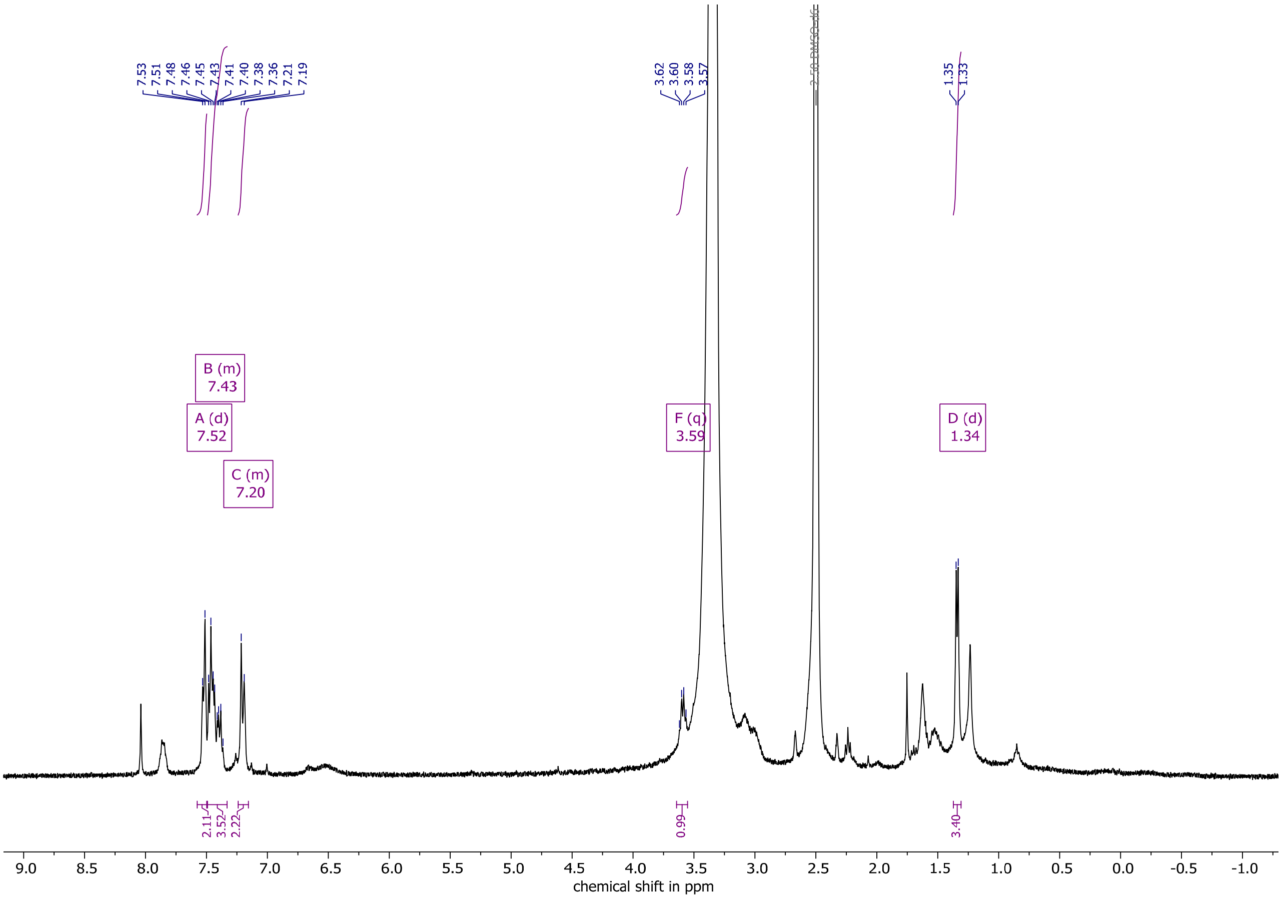

Figure S46. ^1H NMR spectrum of compound **14b** in $(CD_3)_2SO$).

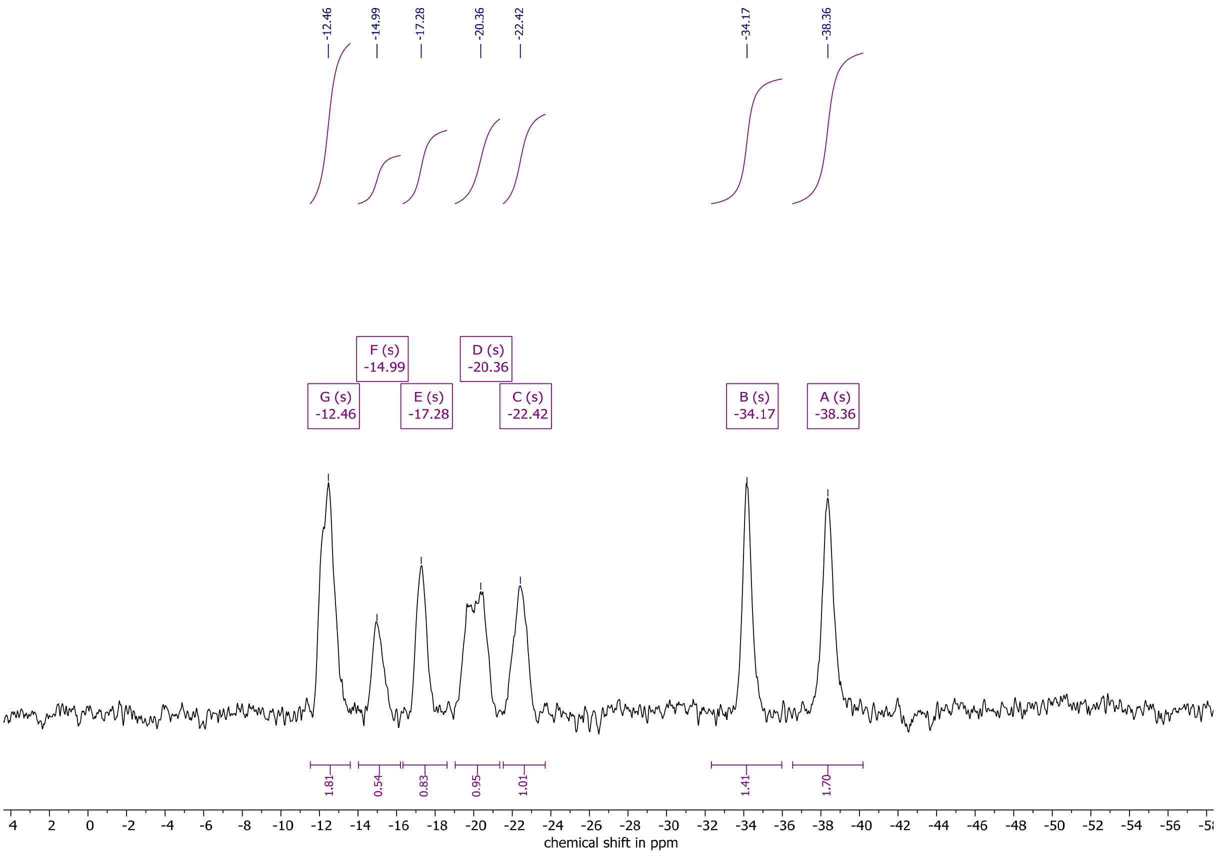

Figure S47. $^{11}B\{^1H\}$ NMR spectrum of compound **14b** in CD_3OD.

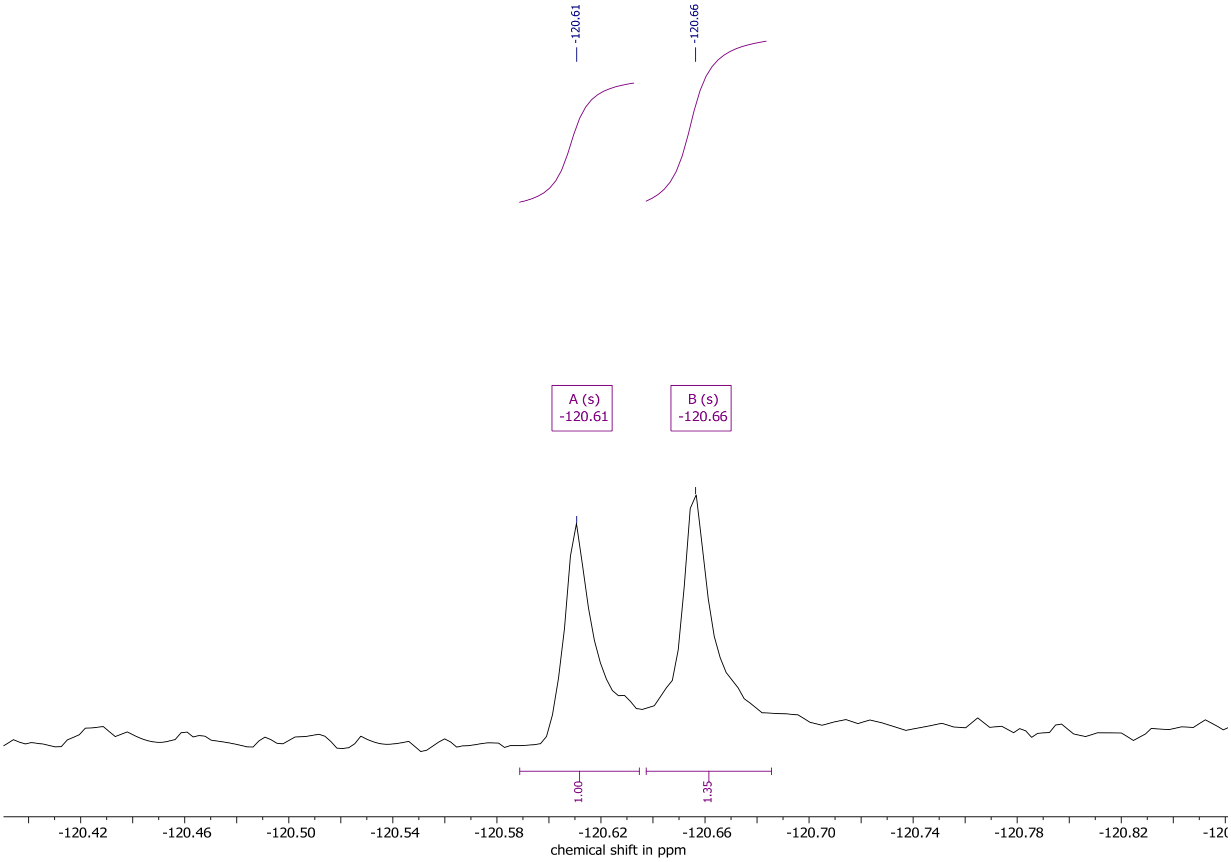

Figure S48. $^{19}F\{^1H\}$ NMR spectrum of compound **14b** in CD_3OD.

2 HR-ESI Mass Spectra of Compounds 6, 7, 9, 10, 13a, 13b, 14a, 14b

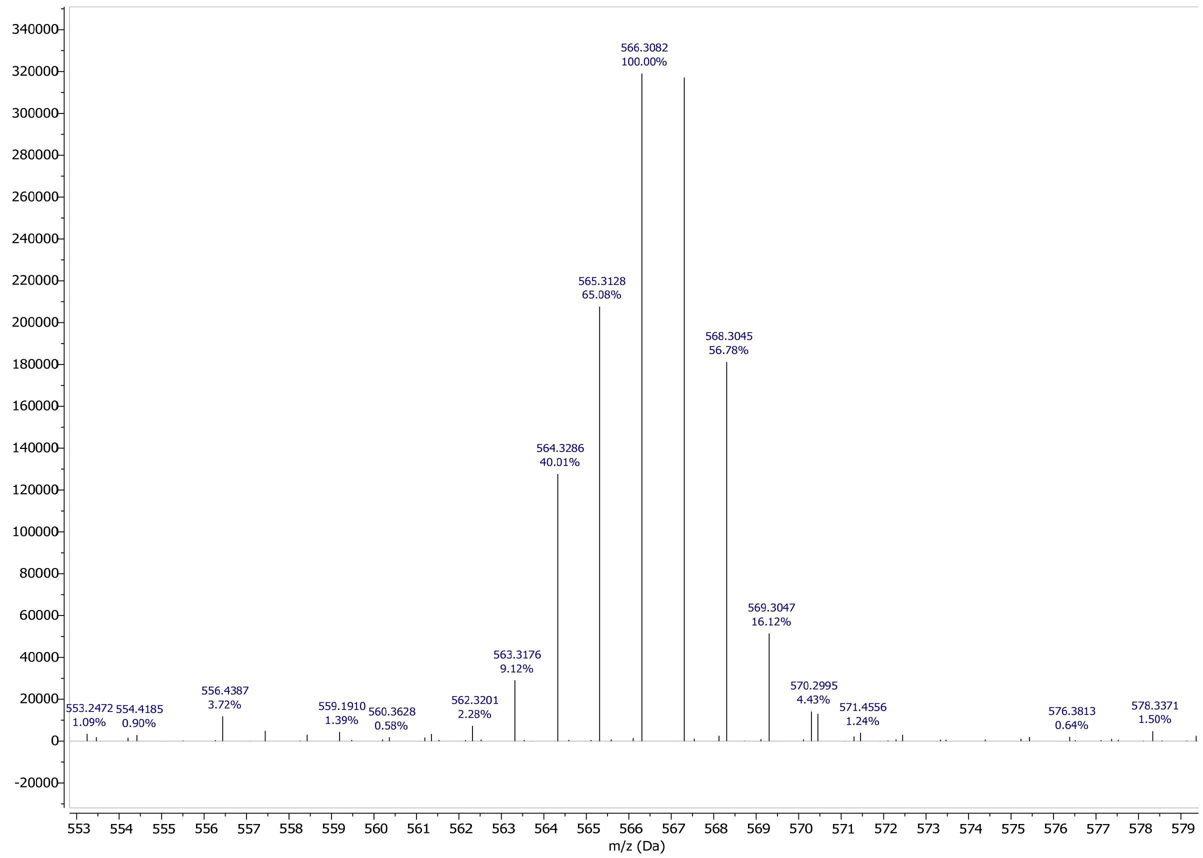

Figure S49. HRMS (ESI+) of compound **6** in CH_3CN.

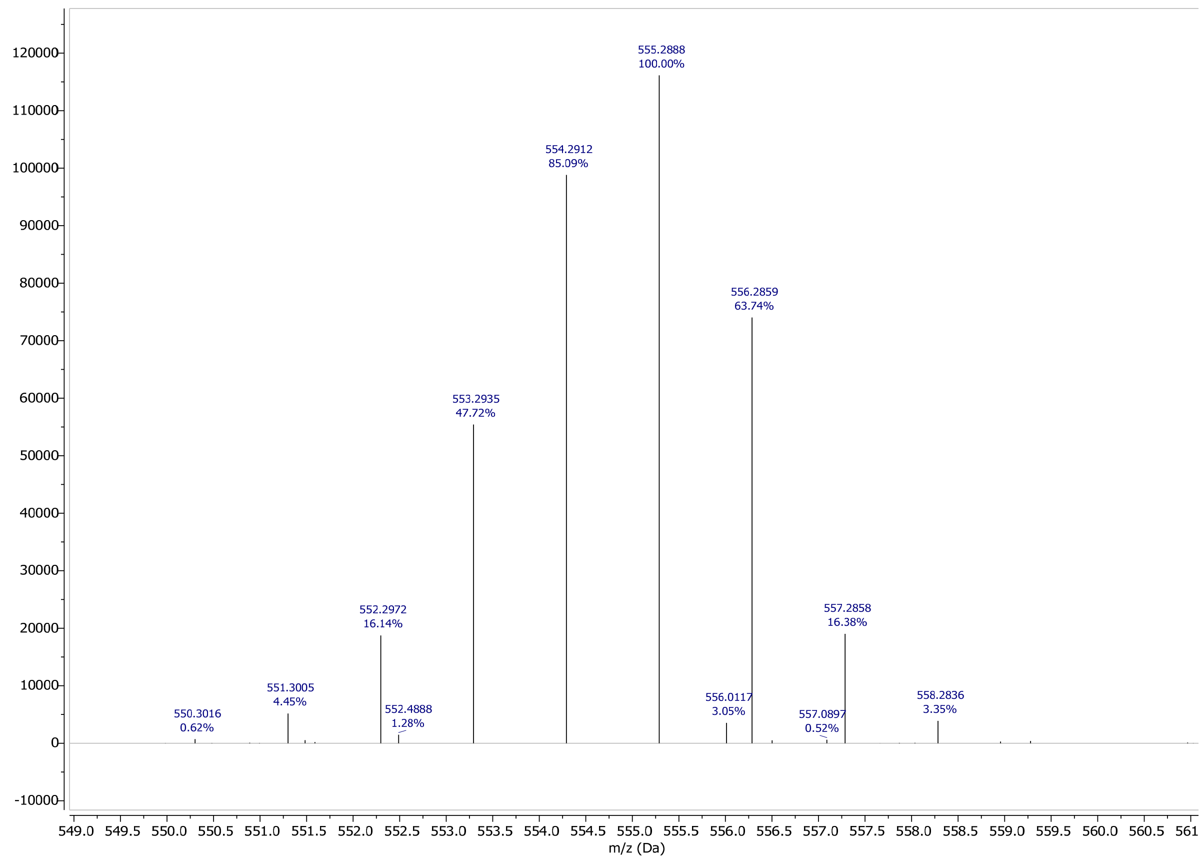

Figure S50. HRMS (ESI-) of compound **7** in MeOH.

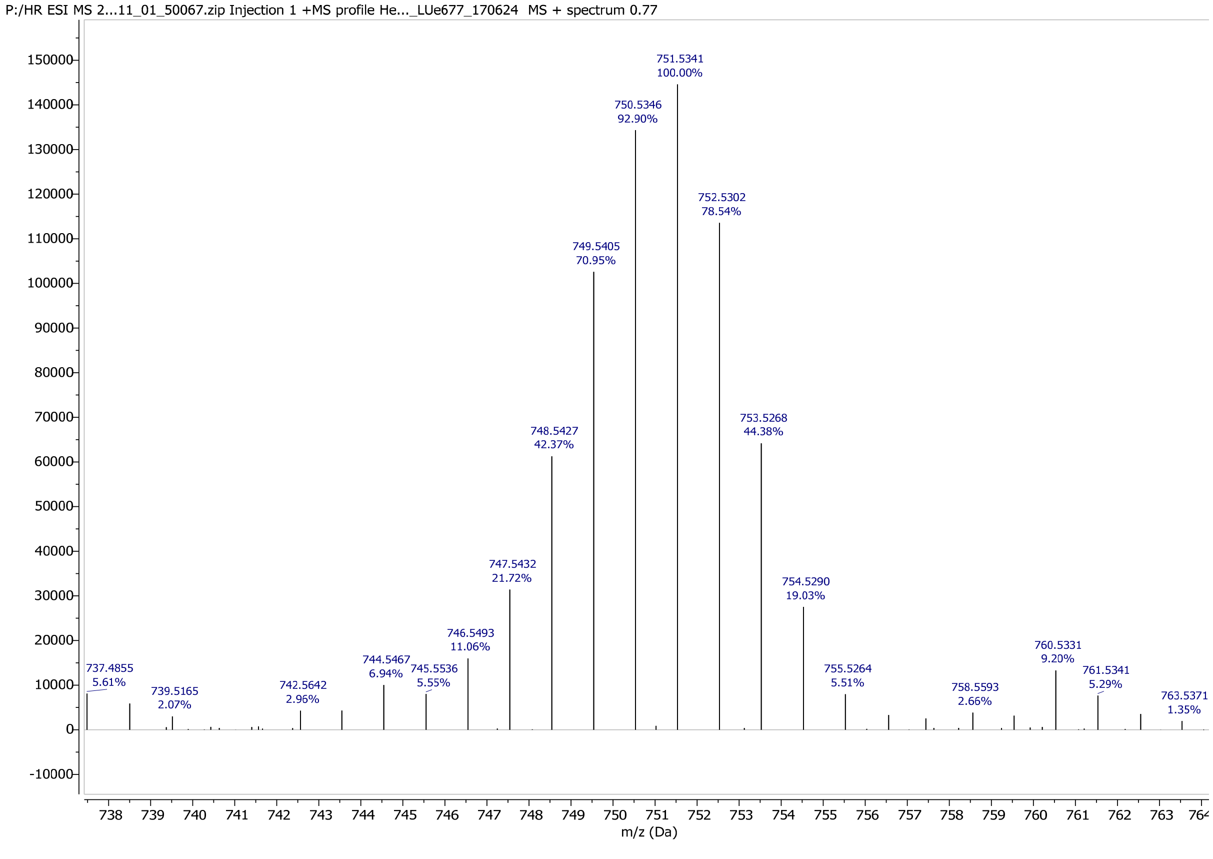

Figure S51. HRMS (ESI+) of compound **9** in CH_3CN.

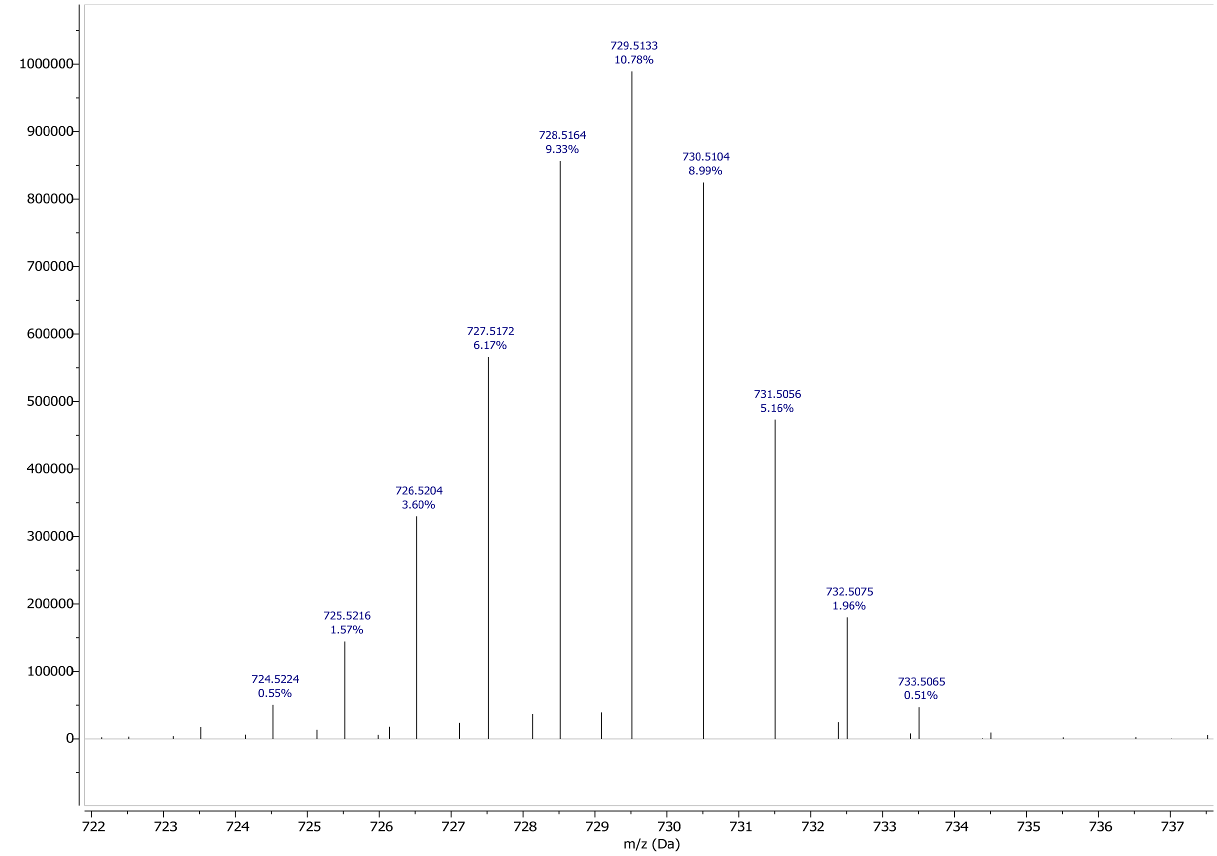

Figure S52. HRMS (ESI-) of compound **10** in MeOH, $[M{-}2Cs{+}H]^{-}$.

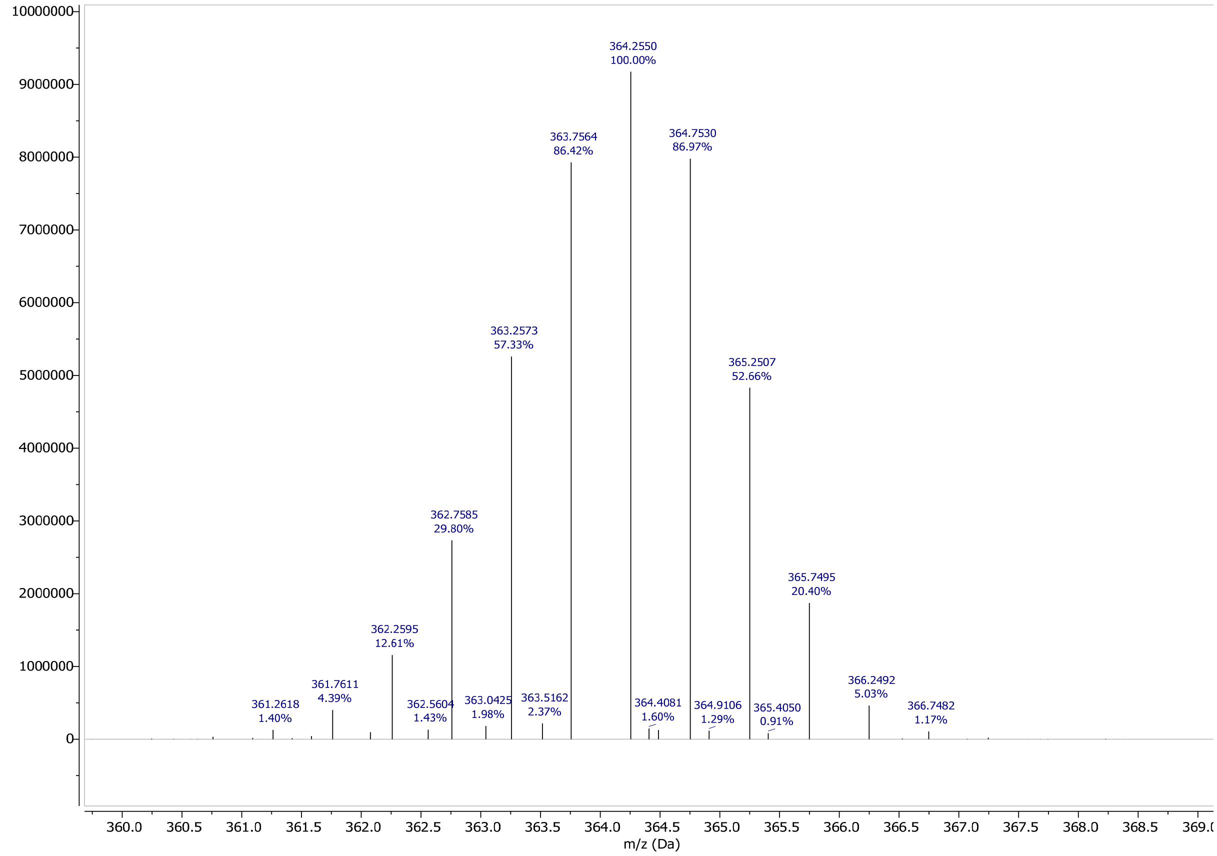

Figure S53. HRMS (ESI-) of compound **10** in MeOH $[M{-}2Cs]^{2-}$.

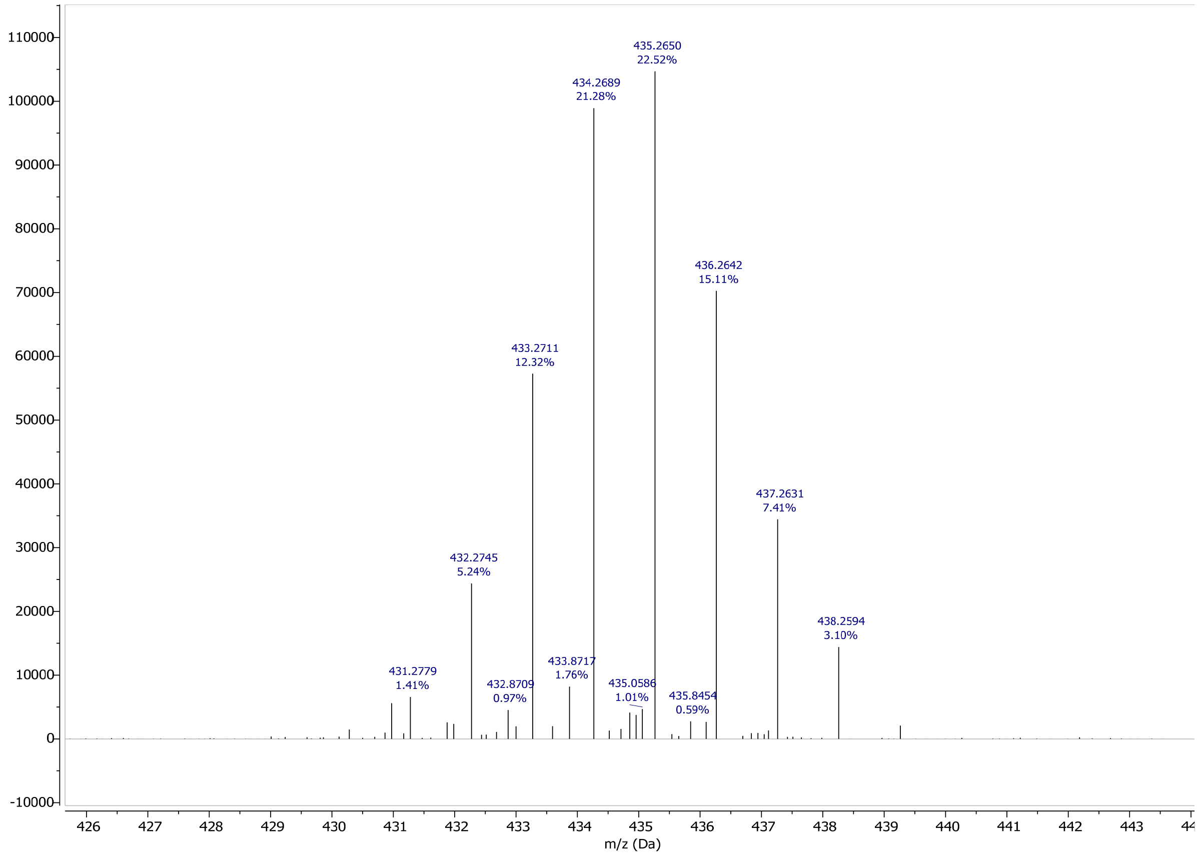

Figure S54. HRMS (ESI-) of compound **13a** in CH_3CN, $[M+Cl]^-$.

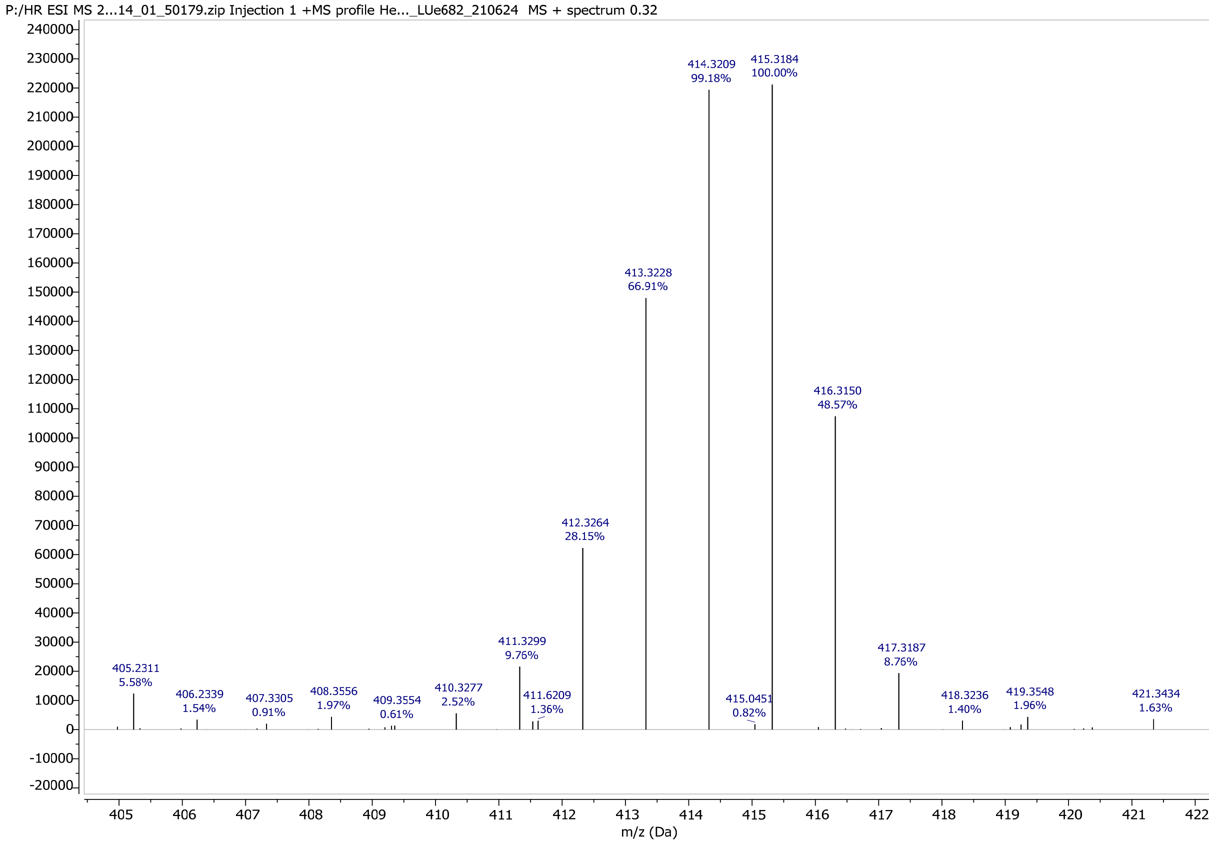

Figure S55. HRMS (ESI+) of compound **13b** in CH_3CN.

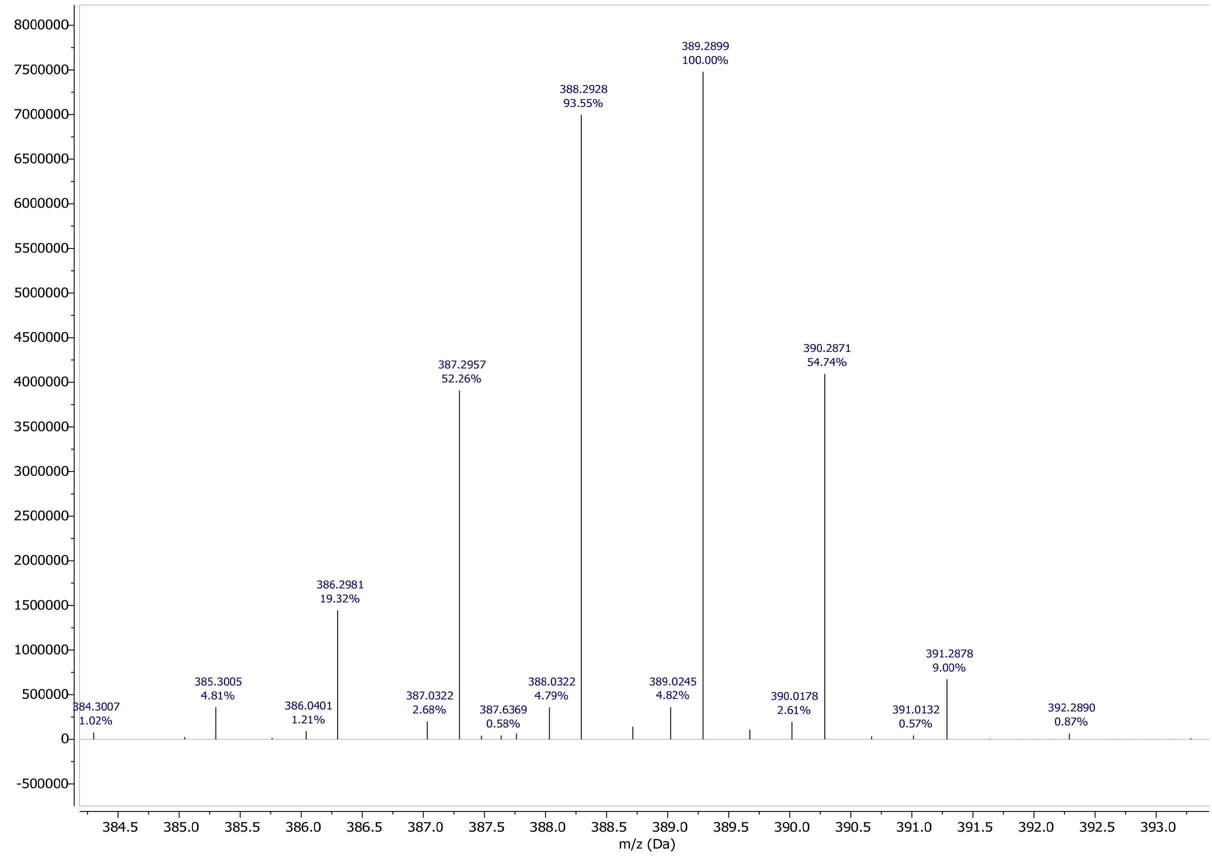

Figure S56. HRMS (ESI-) of compound **14a** in MeOH.

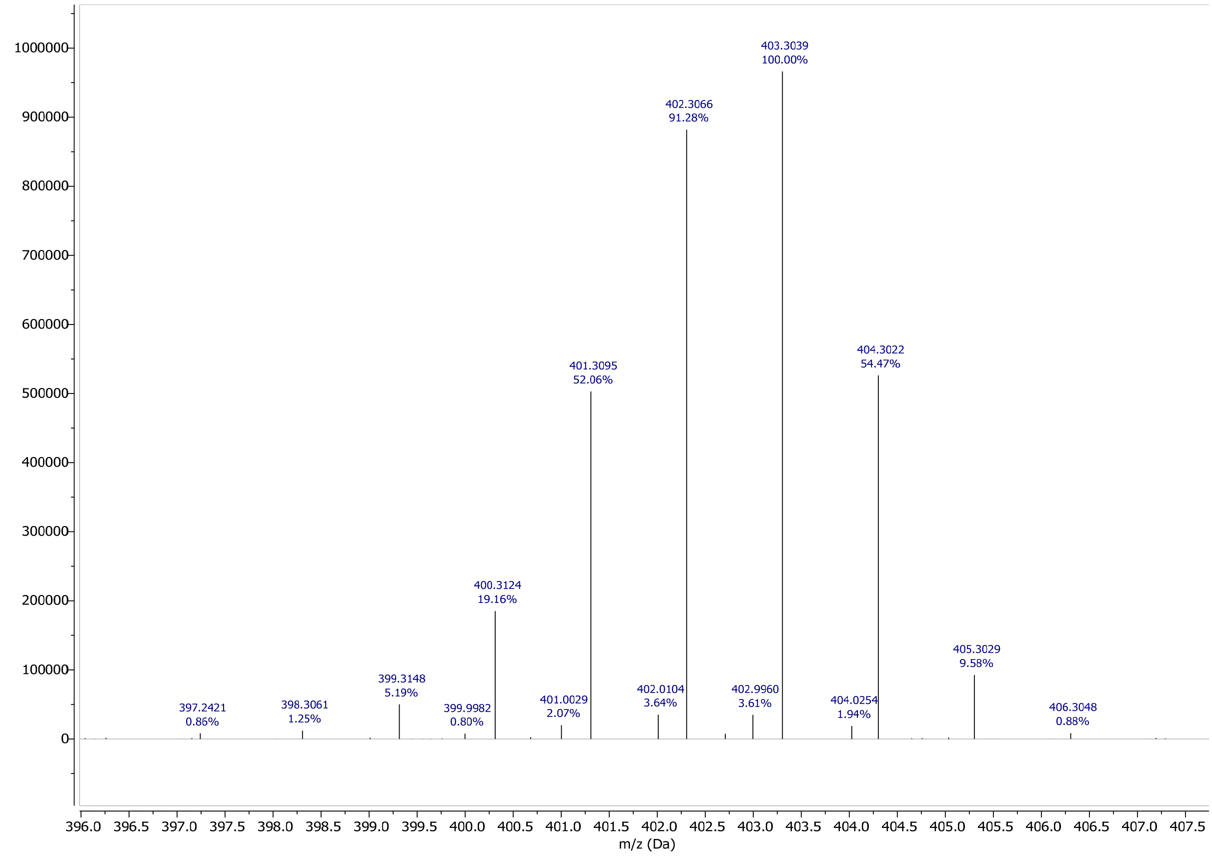

Figure S57. HRMS (ESI-) of compound **14b** in MeOH.

3 Purity Determination of Compounds 6, 7, 9, 10, 13a, 13b, 14a, 14b by HPLC

Purity measurements have been performed with HPLC-UV-MS or analytical HPLC (in that case: UPLC-MS for mass confirmation used prior to that separately) on an RP column. The *ortho*-carborane compounds have been dissolved in CH_3CN and the *nido*-carborane compounds in MeOH or dimethyl sulfoxide (DMSO). All compounds had a purity ≥95%. Broad signals have been observed for the *nido*-carborane derivatives. Double-signals are appearing most likely because of the column that has been used. A compound-related reason was excluded, since variation of gradient (start already with higher degree of eluent B: CH_3CN + 0.1% formic acid) did not resolve the double-peaks. The behavior was always the same.

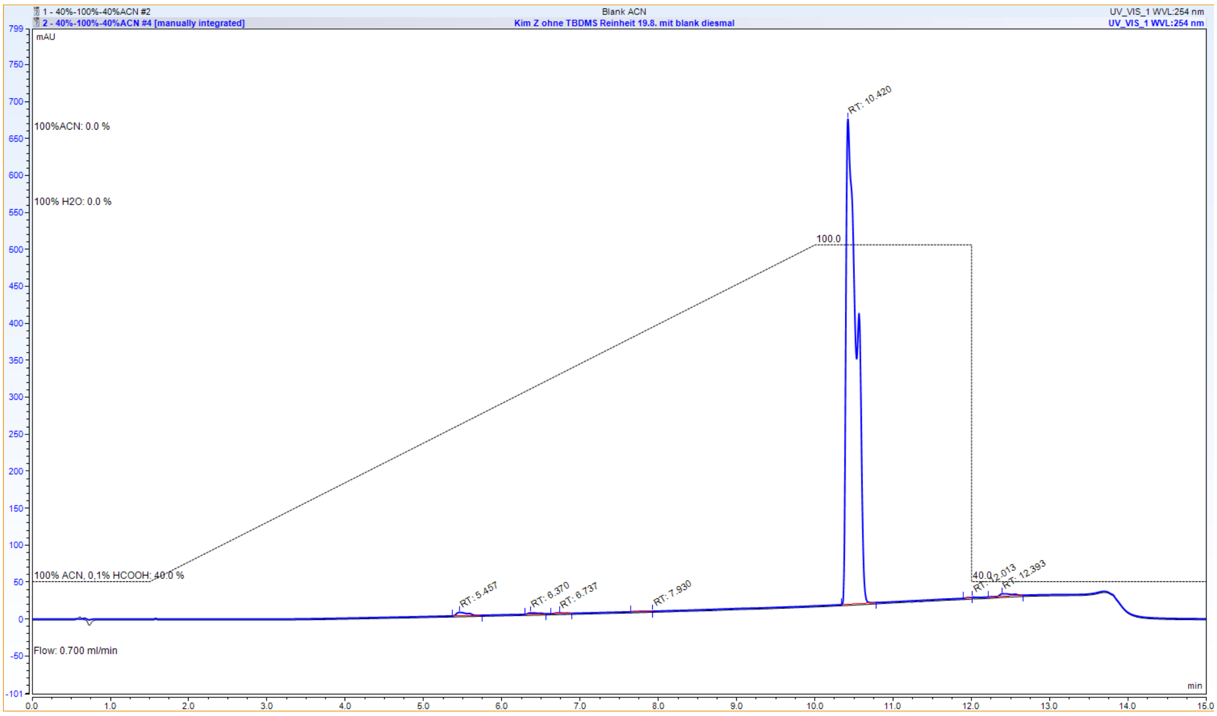

Figure S58. RP-HPLC chromatogram of blank (CH_3CN) and compound **6**, retention time: 10.4 min.

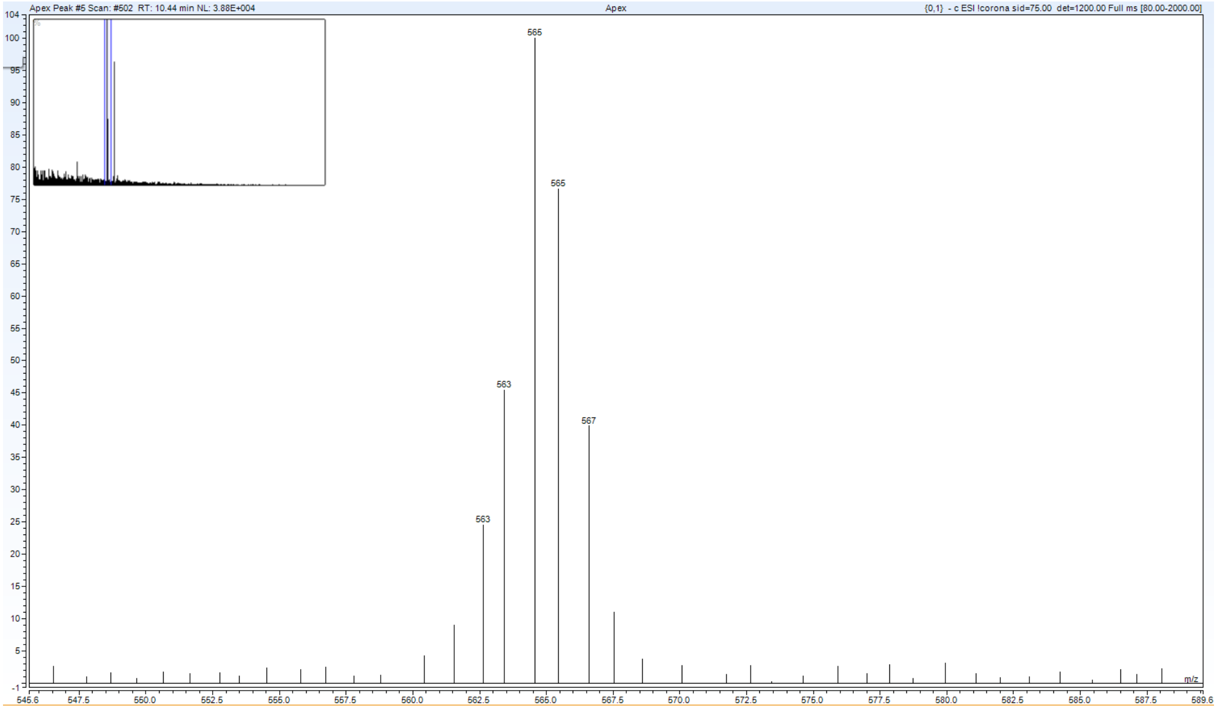

Figure S59. MS(-) of compound **6**, retention time: 10.4 min.

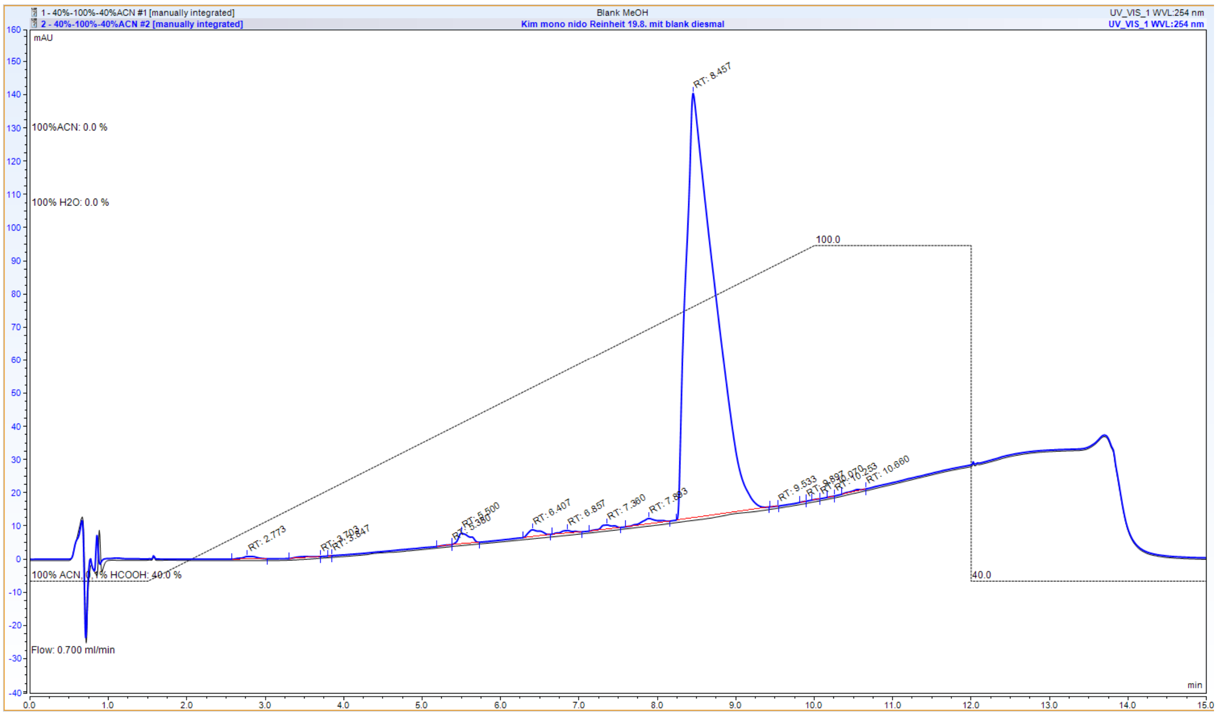

Figure S60. RP-HPLC chromatogram of blank (MeOH) and compound **7**, retention time: 8.5 min.

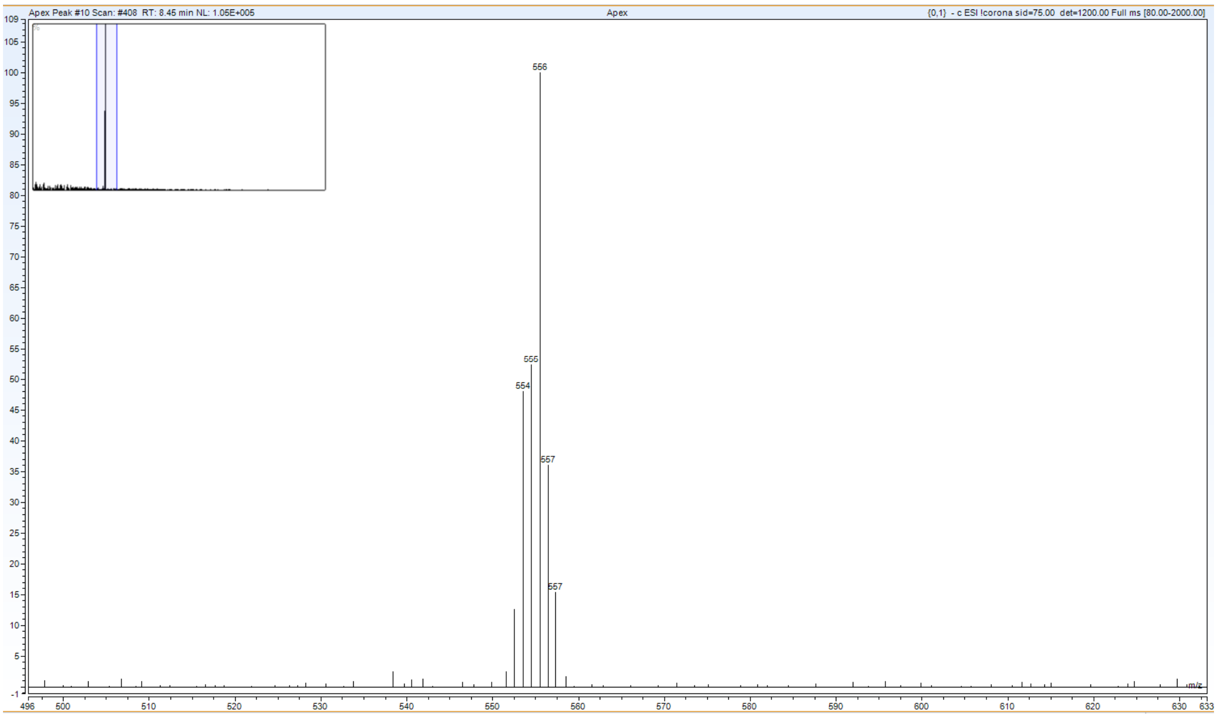

Figure S61. MS(-) of compound **7**, retention time: 8.5 min.

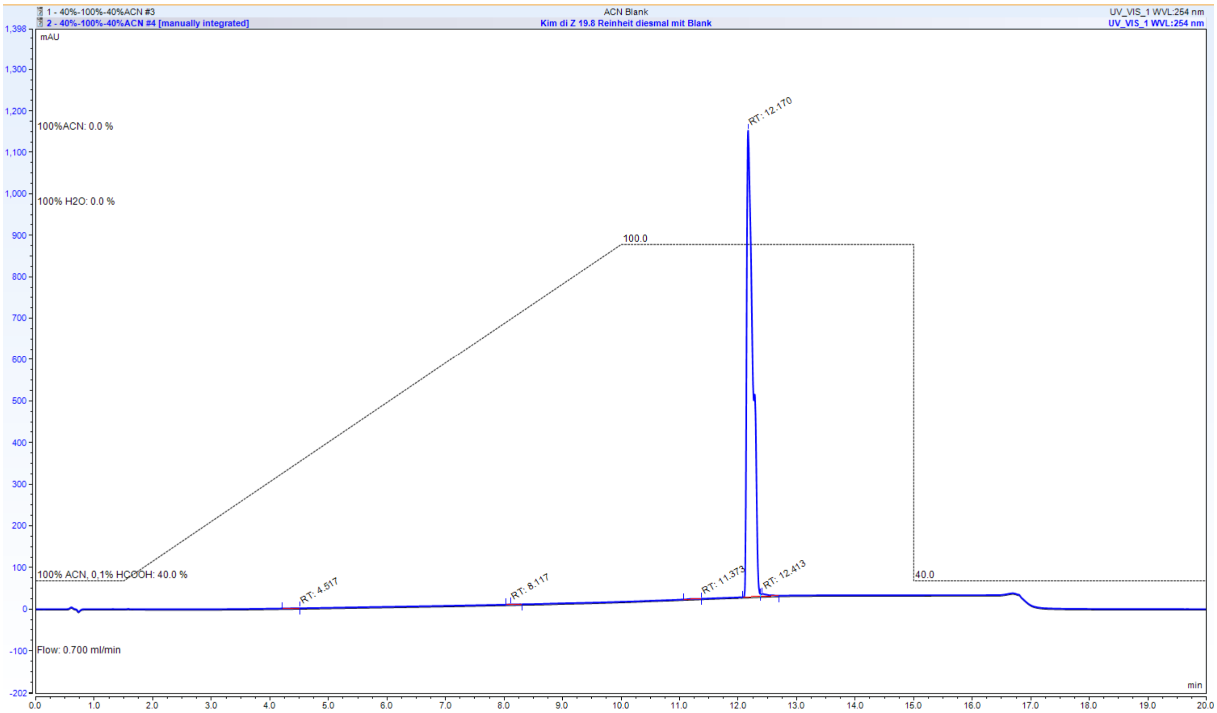

Figure S62. RP-HPLC chromatogram of blank (CH_3CN) and compound **9**, retention time: 12.2 min.

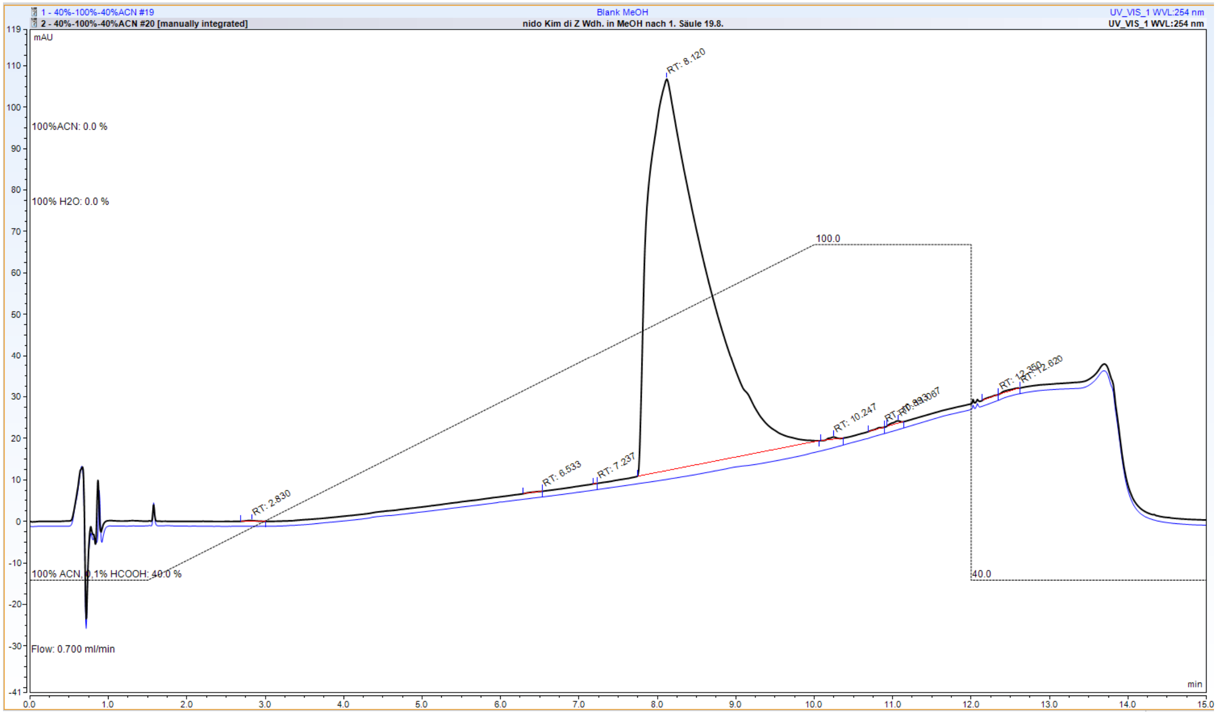

Figure S63. RP-HPLC chromatogram of blank (MeOH) and compound **10**, retention time: 8.1 min.

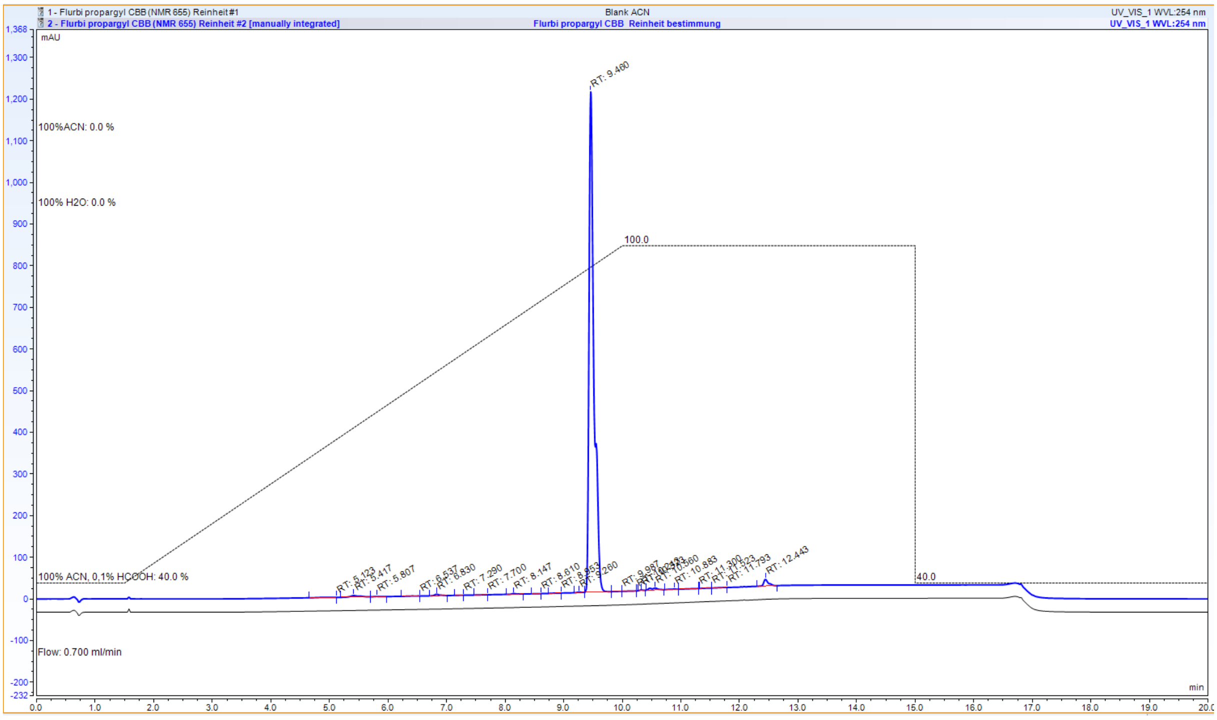

Figure S64. RP-HPLC chromatogram of blank (CH_3CN) and compound **13a**, retention time: 9.5 min.

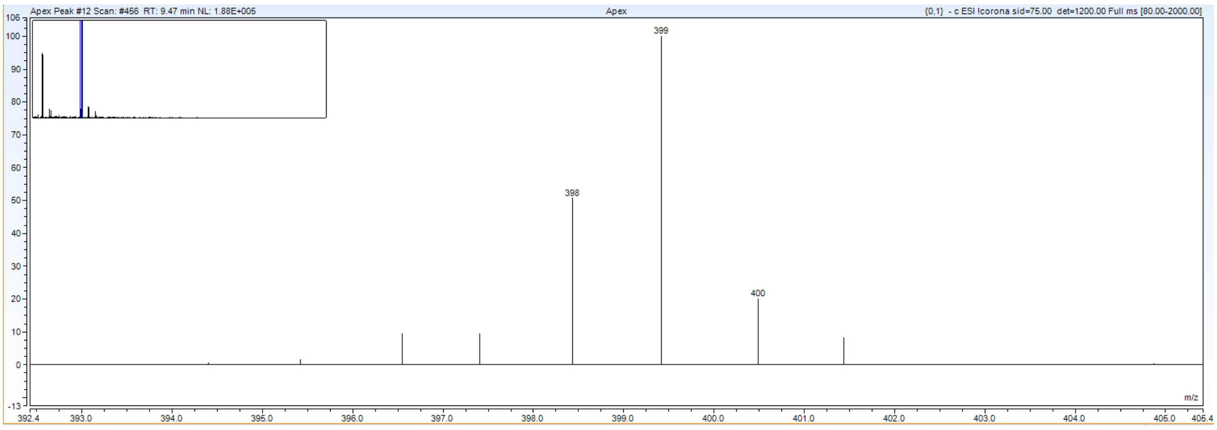

Figure S65. MS(-) of compound **13a**, retention time: 9.5 min.

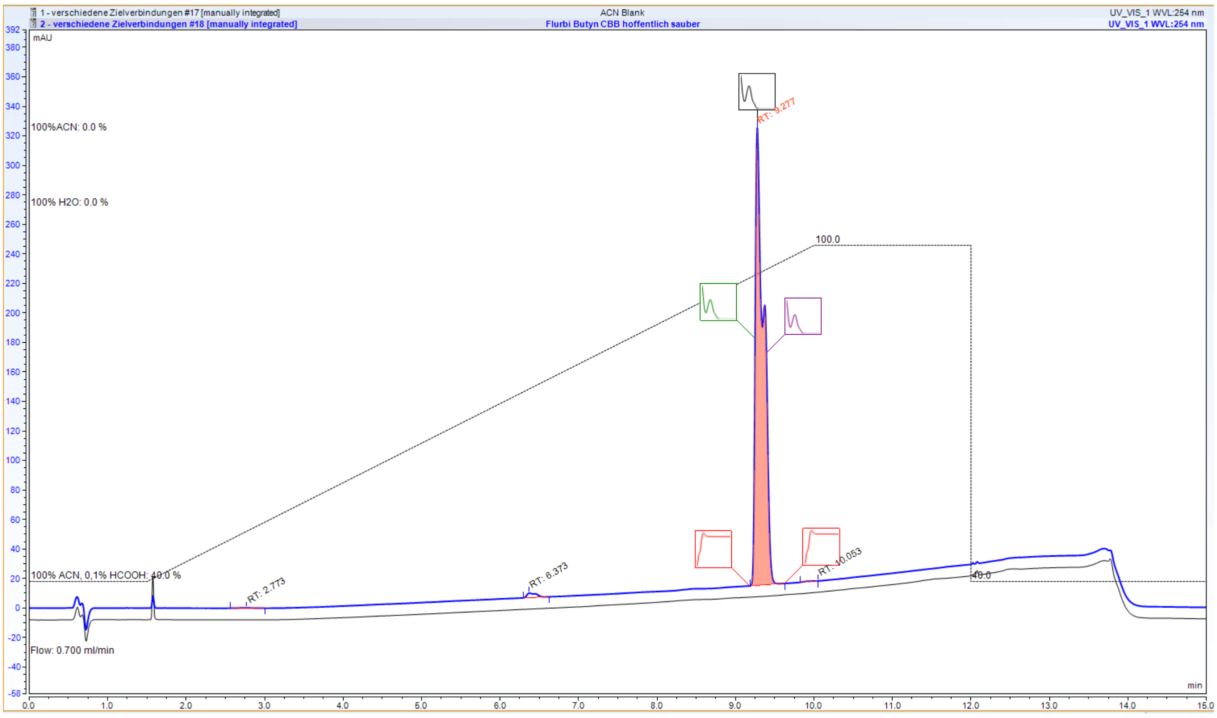

Figure S66. RP-HPLC chromatogram of blank (CH_3CN) and compound **13b**, retention time: 9.3 min.

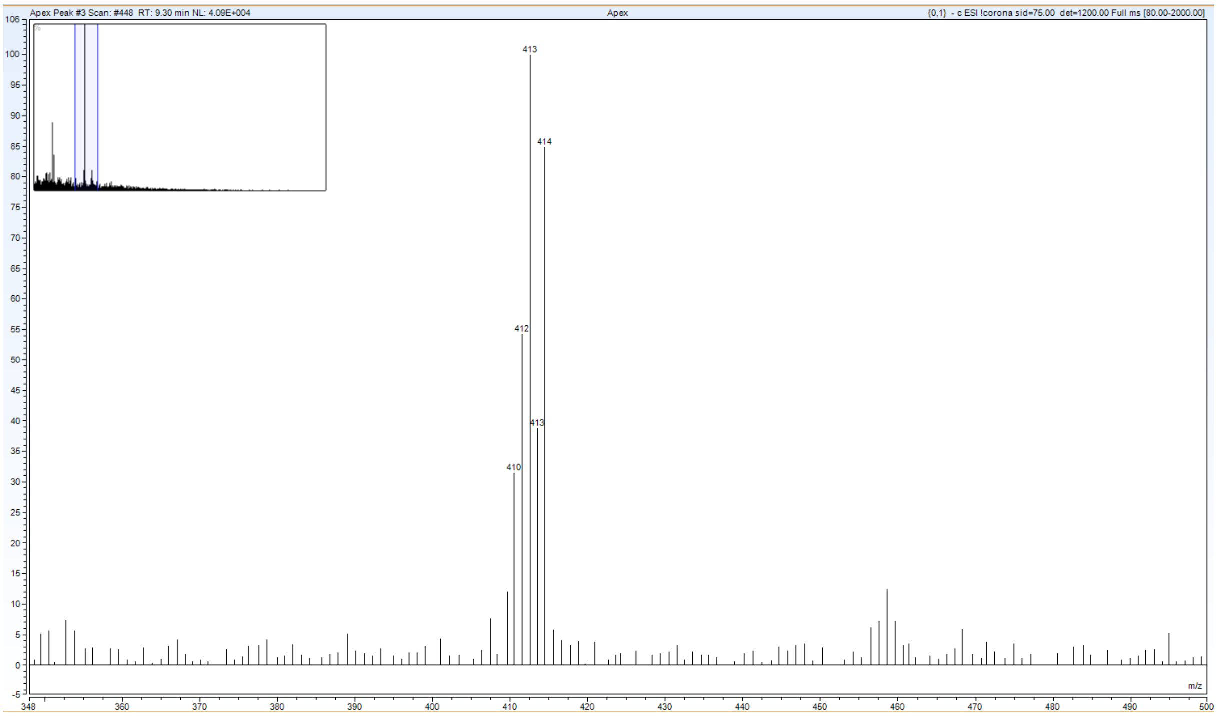

Figure S67. MS(-) of compound **13b**, retention time: 9.3 min.

Compound 14a

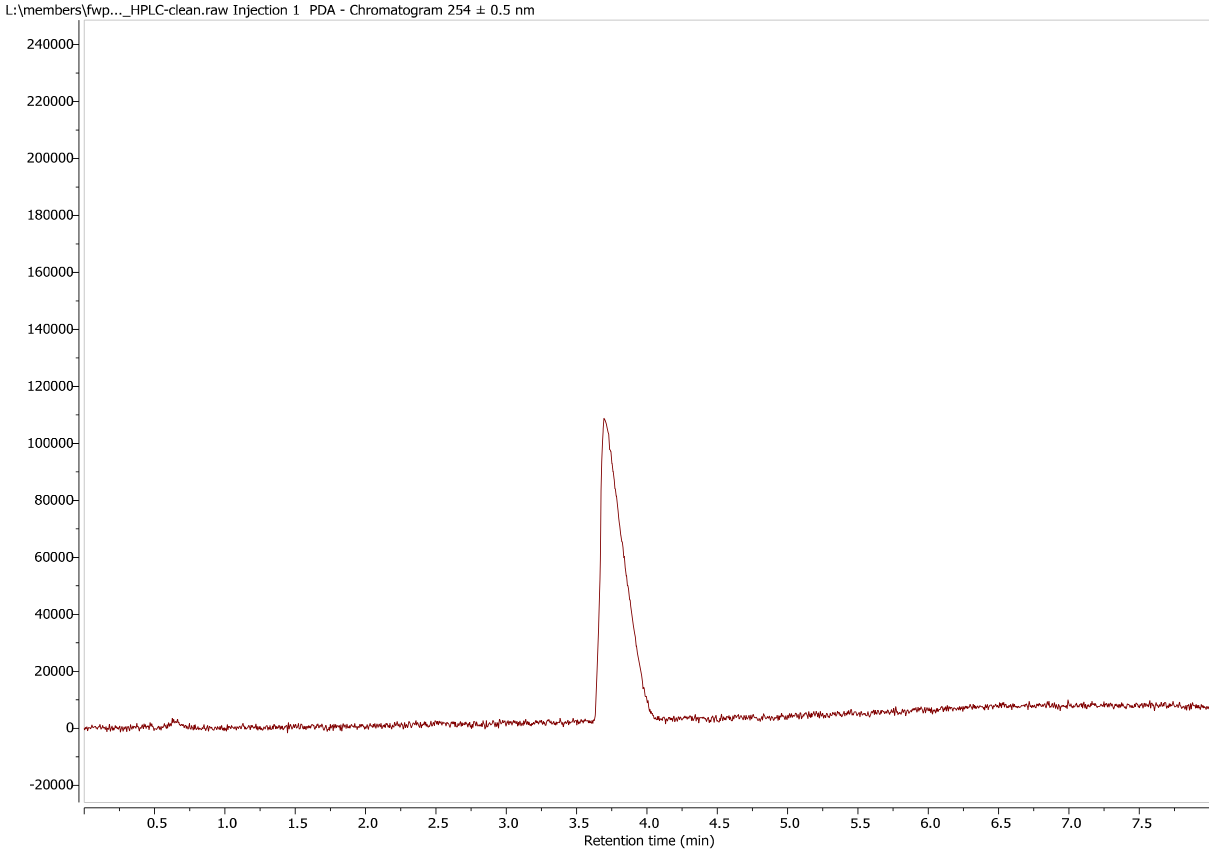

Figure S68. RP-UHPLC chromatogram of compound **14a**, retention time: 3.7 min.

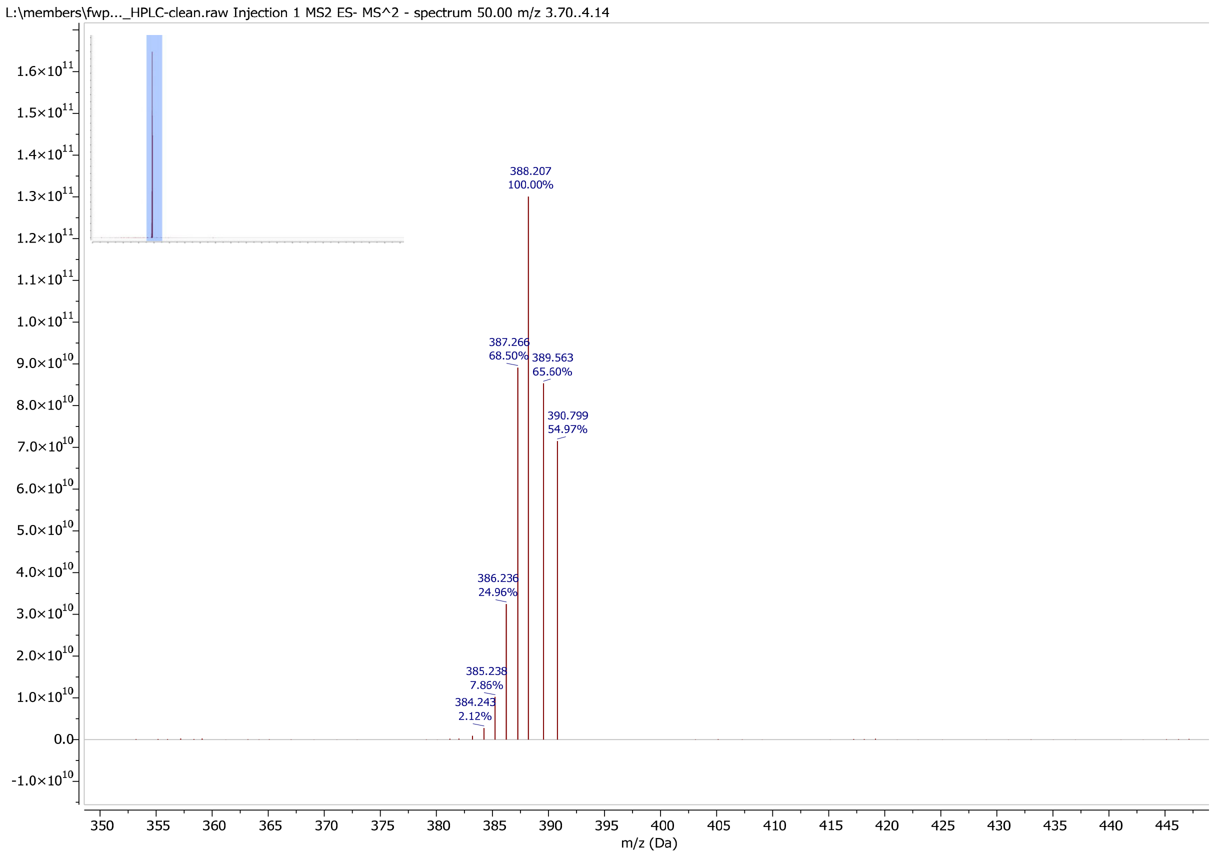

Figure S69. MS(-) of compound **14a**, retention time: 3.7 min.

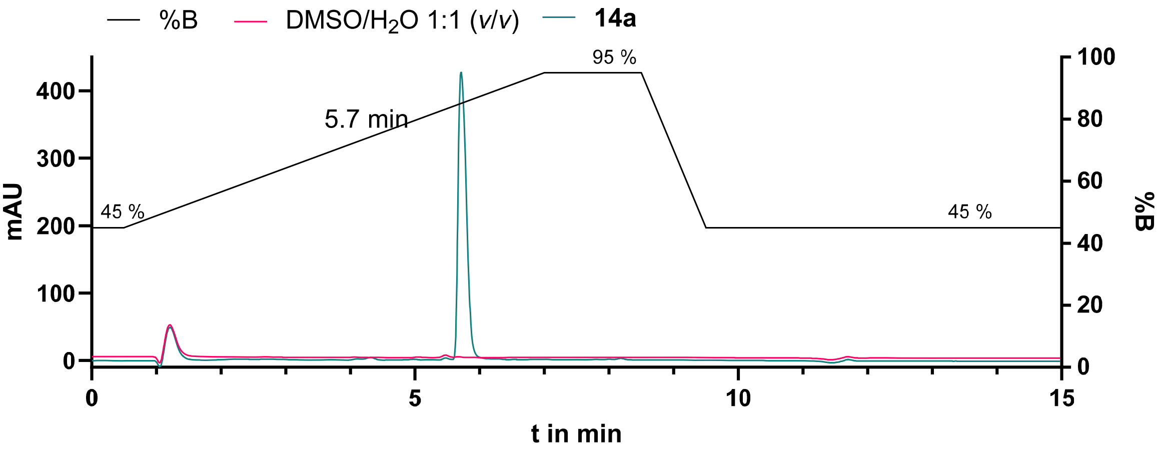

Figure S70. RP-HPLC chromatogram of compound **14a**, retention time: 5.7 min.

Compound 14b

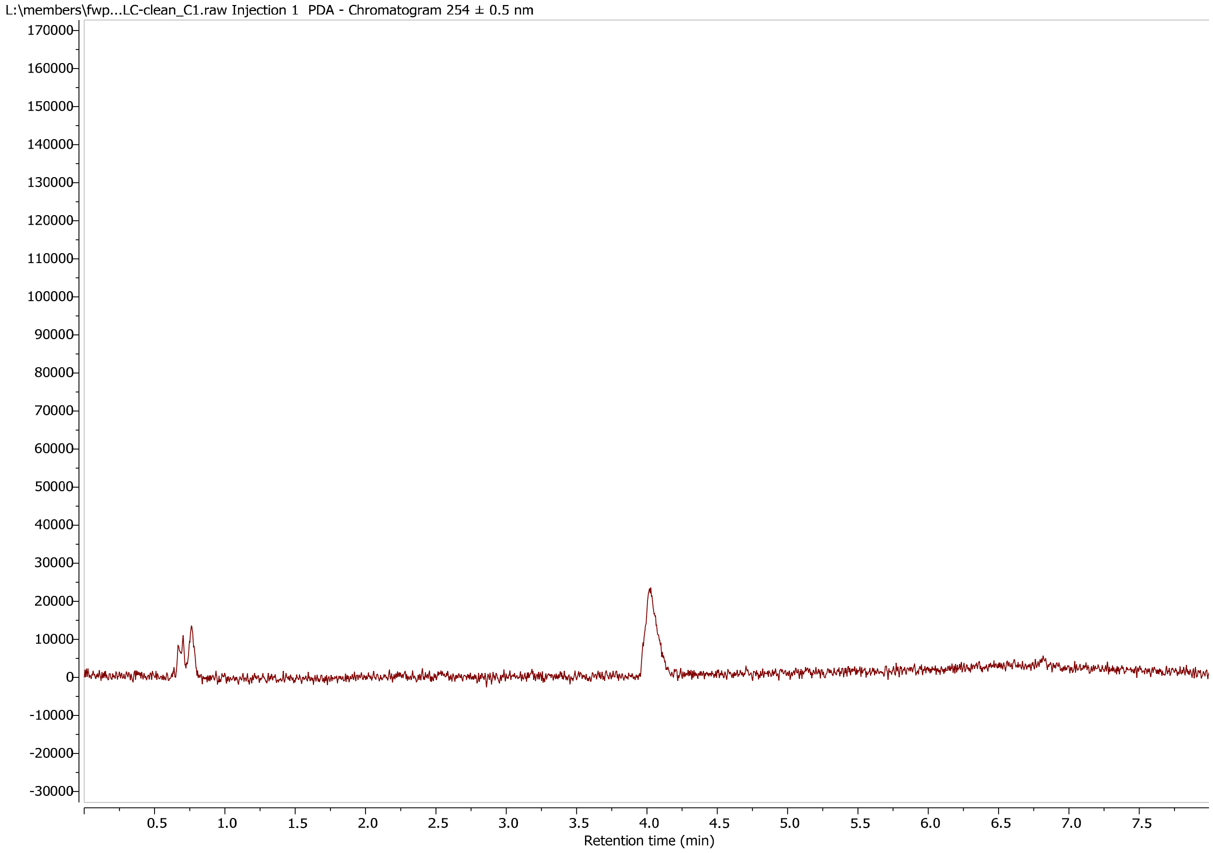

Figure S*71*. RP-UHPLC chromatogram of compound **14b**, retention time: 4.0 min.

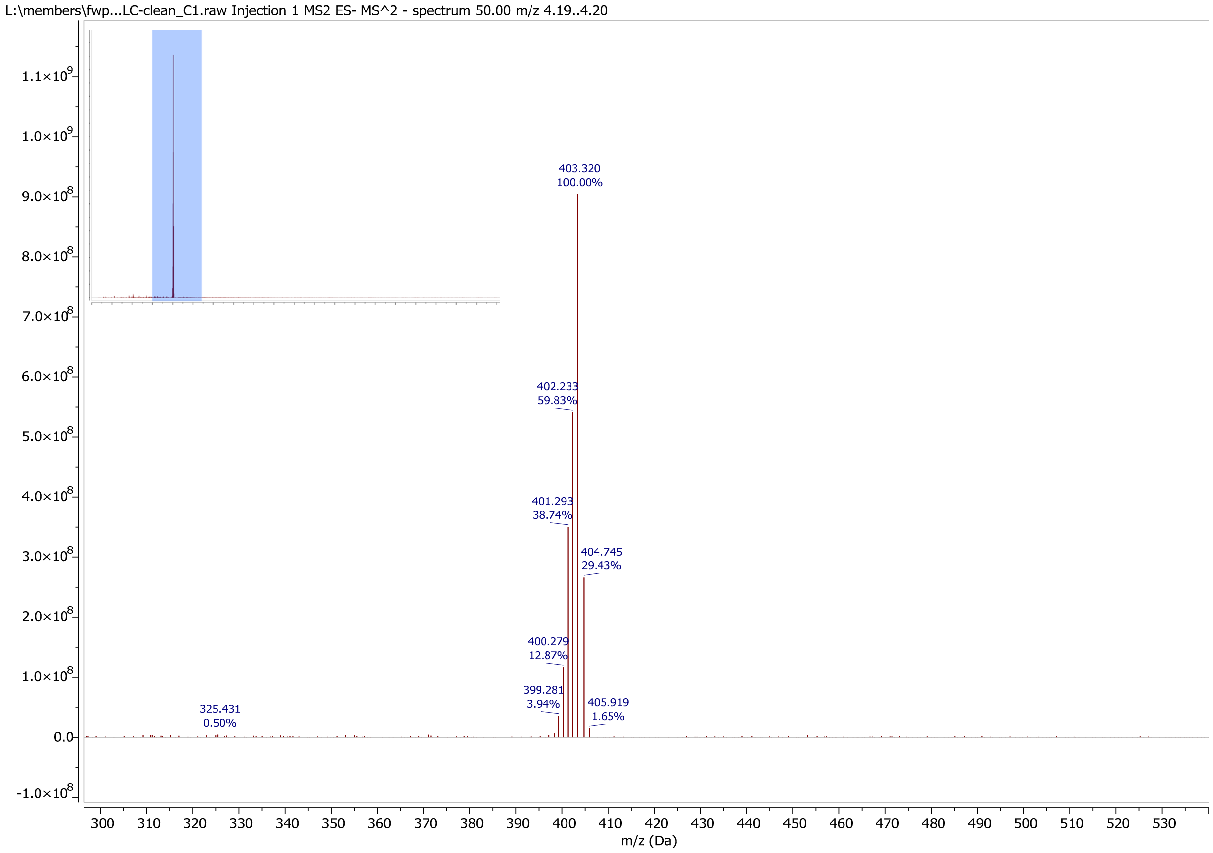

Figure S72. MS(-) of compound **14b**, retention time: 4.0 min.

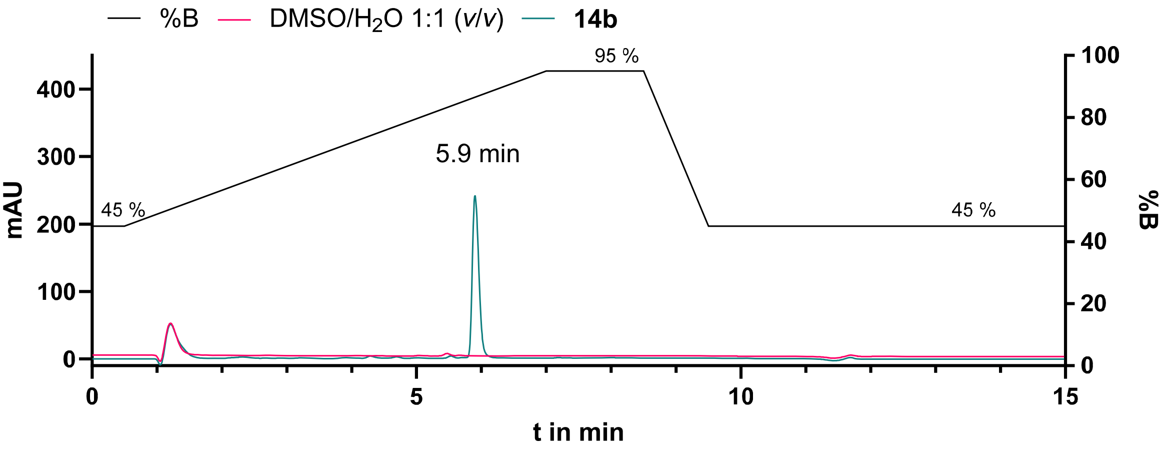

Figure S73. RP-HPLC chromatogram of compound **14b**, retention time: 5.9 min.

4 Stability Determination of Compounds 6, 7, 9, 10, 13a, 13b, 14a, 14b by HPLC

A few milligrams of each compound have been dissolved in 1 mL DMSO, which was stored over molecular sieve. 50-100 µL of the DMSO solution have been transferred into a separate vial together with the same amount of LC-MS grade water. The mixture was measured directly after water addition and in time intervals from 20 min to 1 d by HPLC-UV-MS or analytical HPLC measurements. All compounds were stable for at least one day except for compound **13a**. Since the biological results, did not show promising values for this *ortho*-carborane derivative, but rather for its *nido*-carborane analog, which was anyway the most prominent side product, no further stability measurements have been performed.

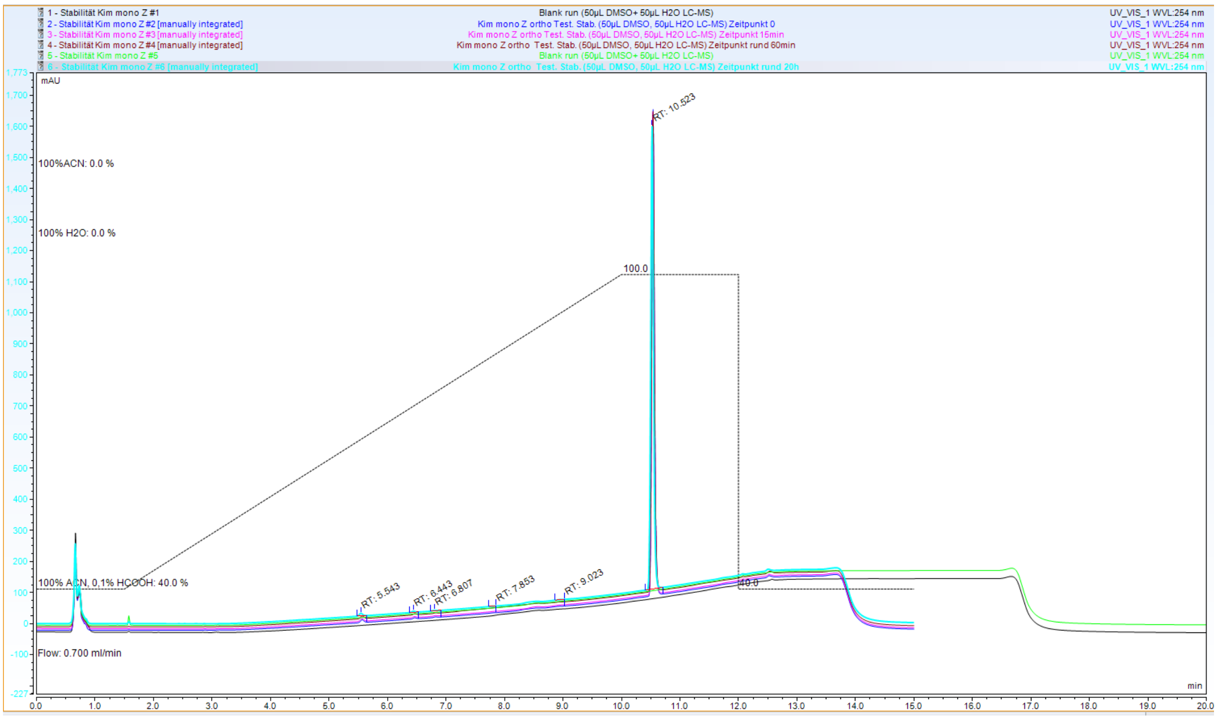

Figure S74. RP-HPLC chromatograms of blank (DMSO/H_2O, black and green) and compound **6**, retention time: 10.5 min, purity after 1 d: >98%.

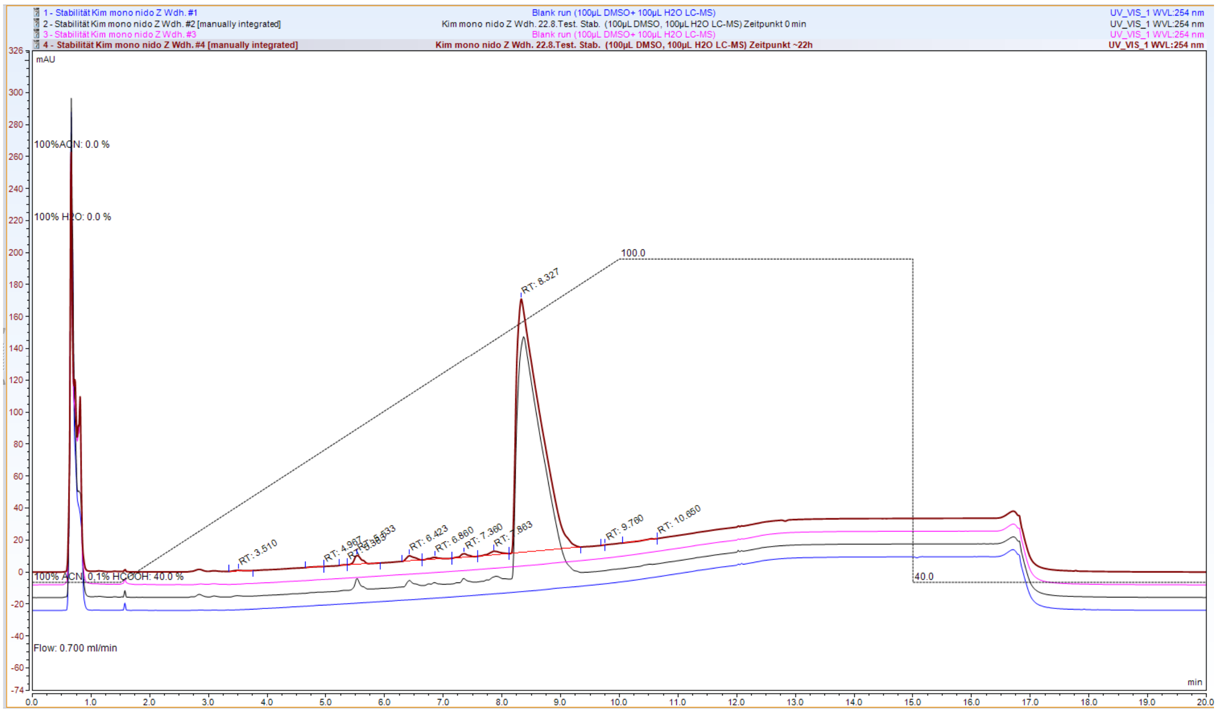

Figure S75. RP-HPLC chromatograms of blank (DMSO/H_2O, blue and pink) and compound **7**, retention time: 8.3 min, purity after 1 d: >96%.

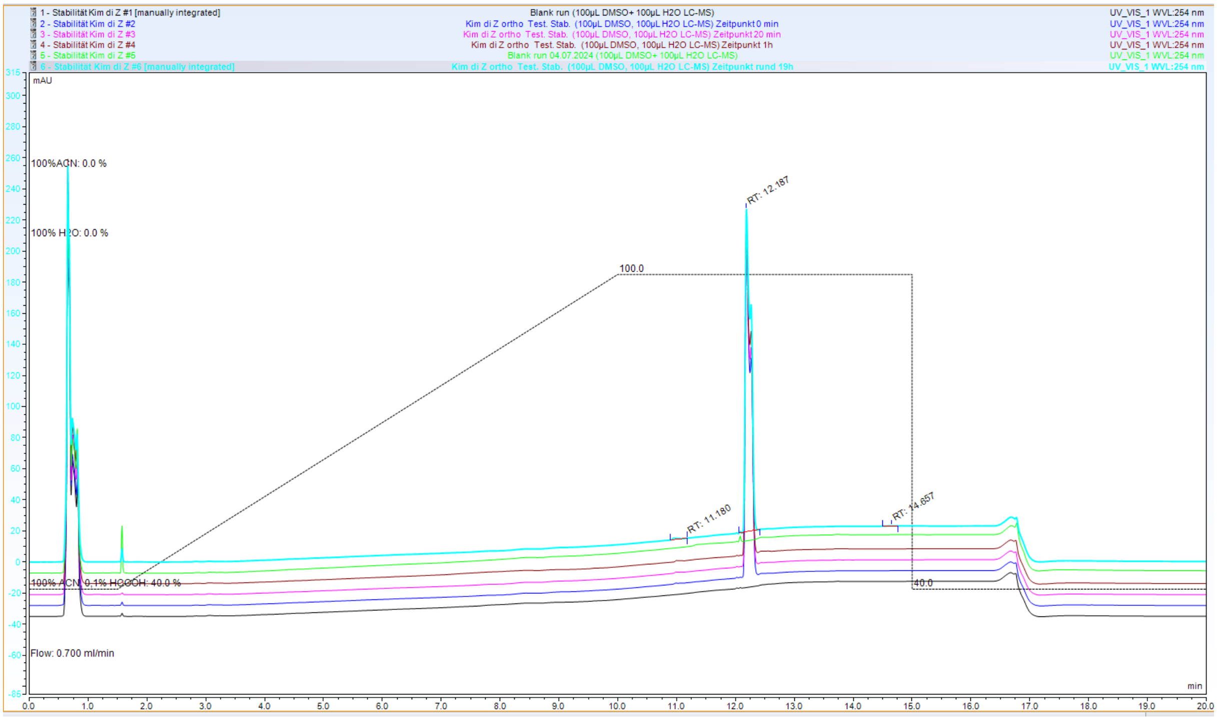

Figure S76. RP-HPLC chromatograms of blank (DMSO/H_2O, black and green) and compound **9**, retention time: 12.2 min, purity after 1 d: >99%.

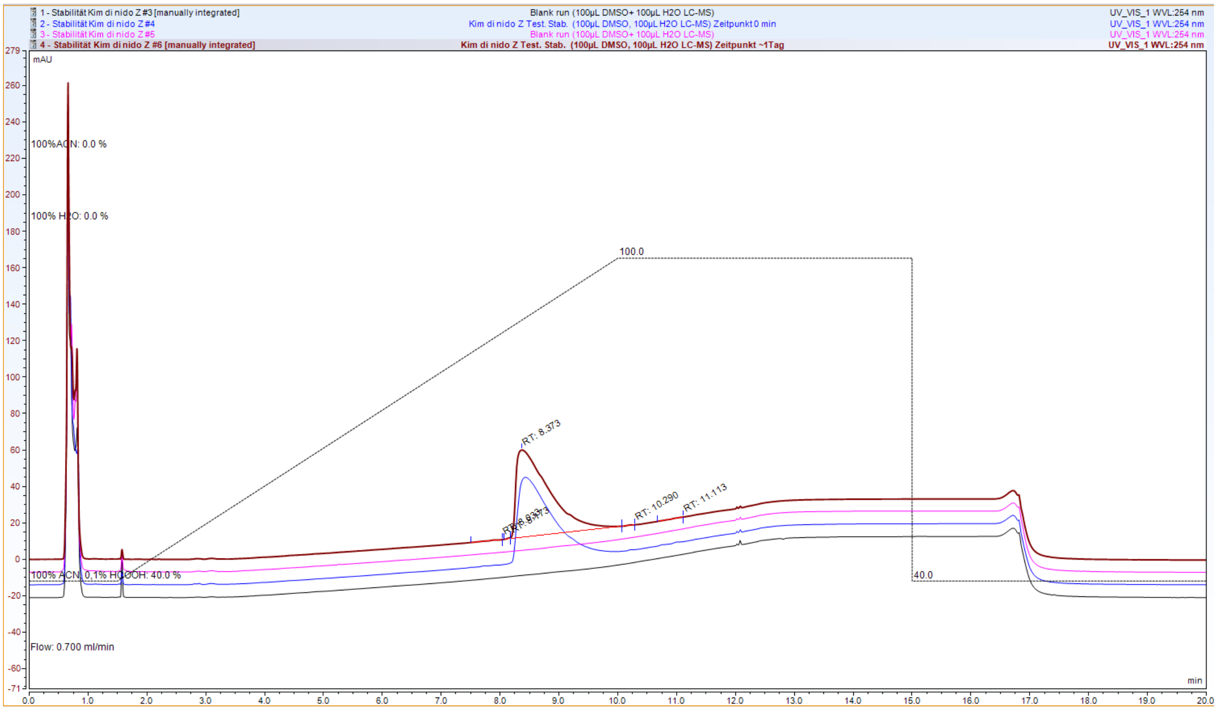

Figure S 77. RP-HPLC chromatograms of blank (DMSO/H_2O, black and pink) and compound **10**, retention time: 8.4 min, purity after 1 d: >99%.

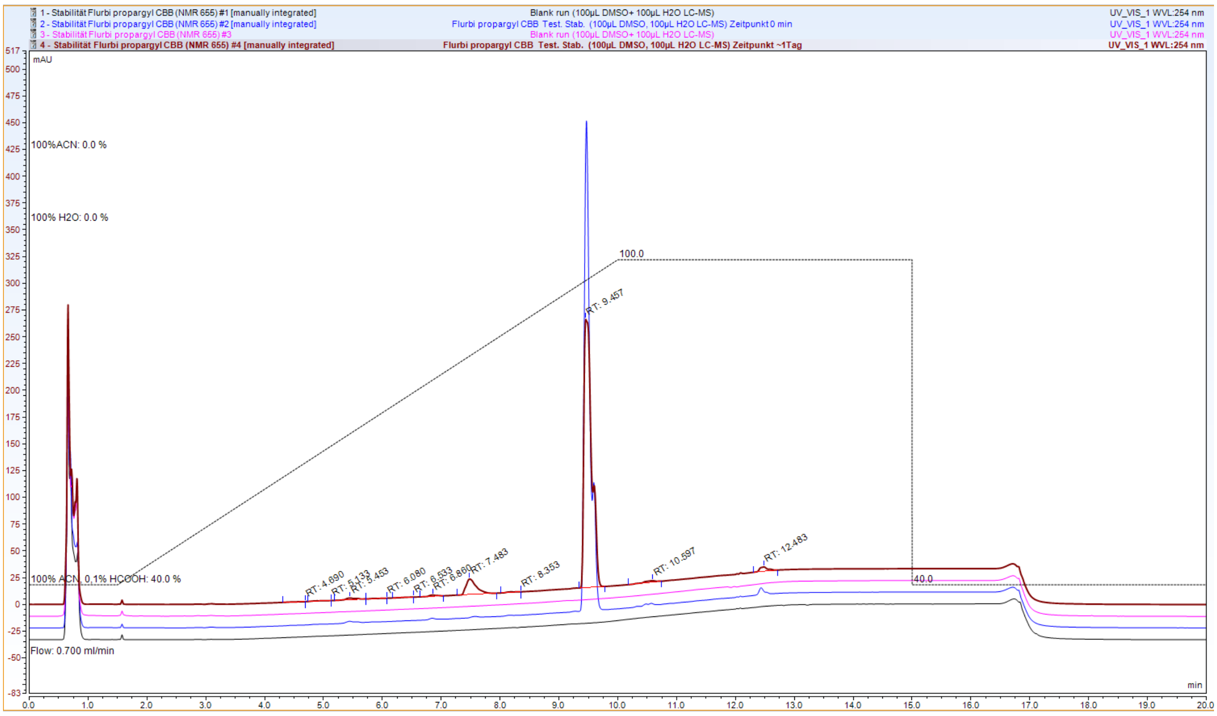

Figure S78. RP-HPLC chromatograms of blank (DMSO/H_2O, black and pink) and compound **13a**, retention time: 9.5 min, purity after 1 d: ~89%.

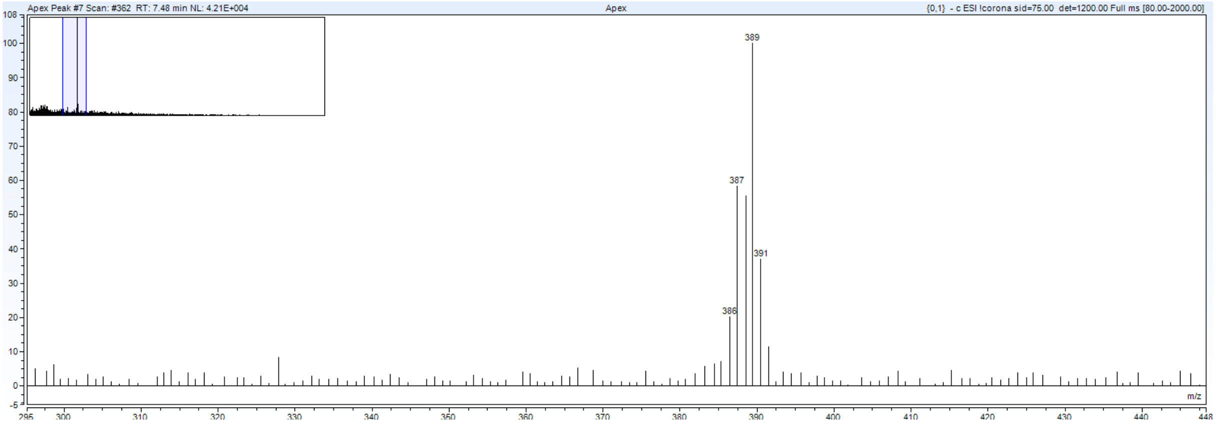

Figure S79. MS(-) of compound **13a**, retention time: 7.5 min, side product = *nido*-carborane derivative of **13a**.

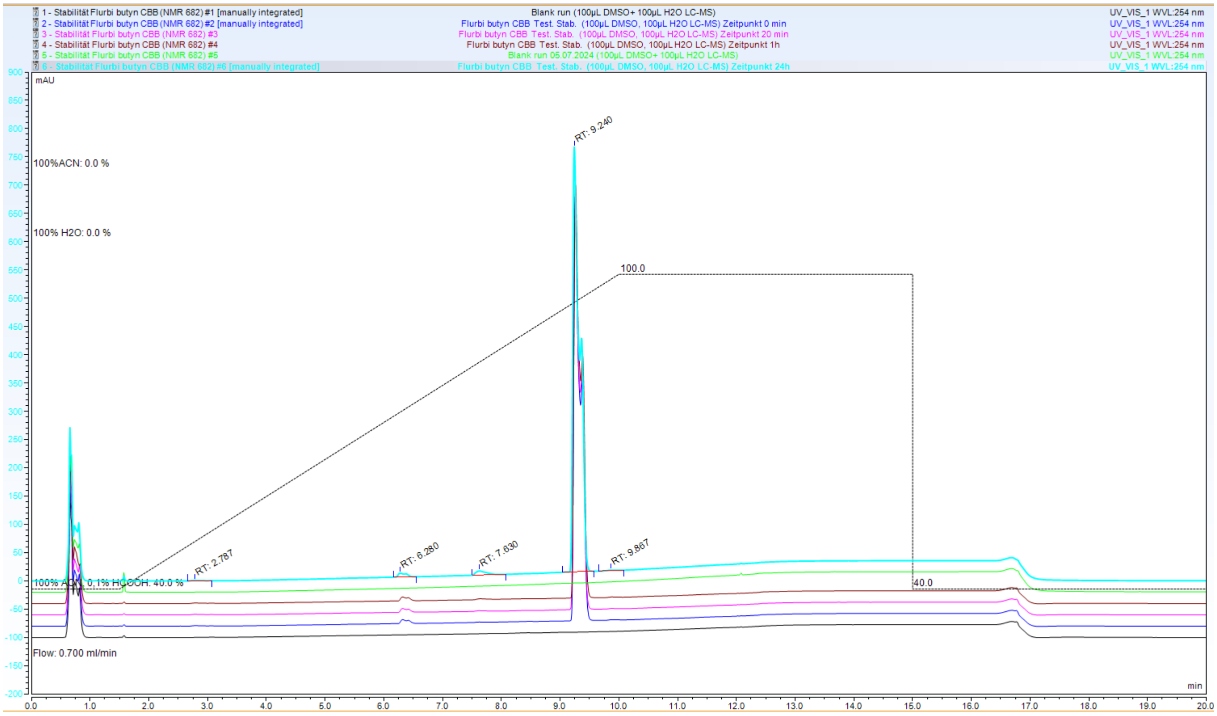

Figure S80. RP-HPLC chromatograms of blank (DMSO/H_2O, black and green) and compound **13b**, retention time: 9.2 min, purity after 1 d: >97%.

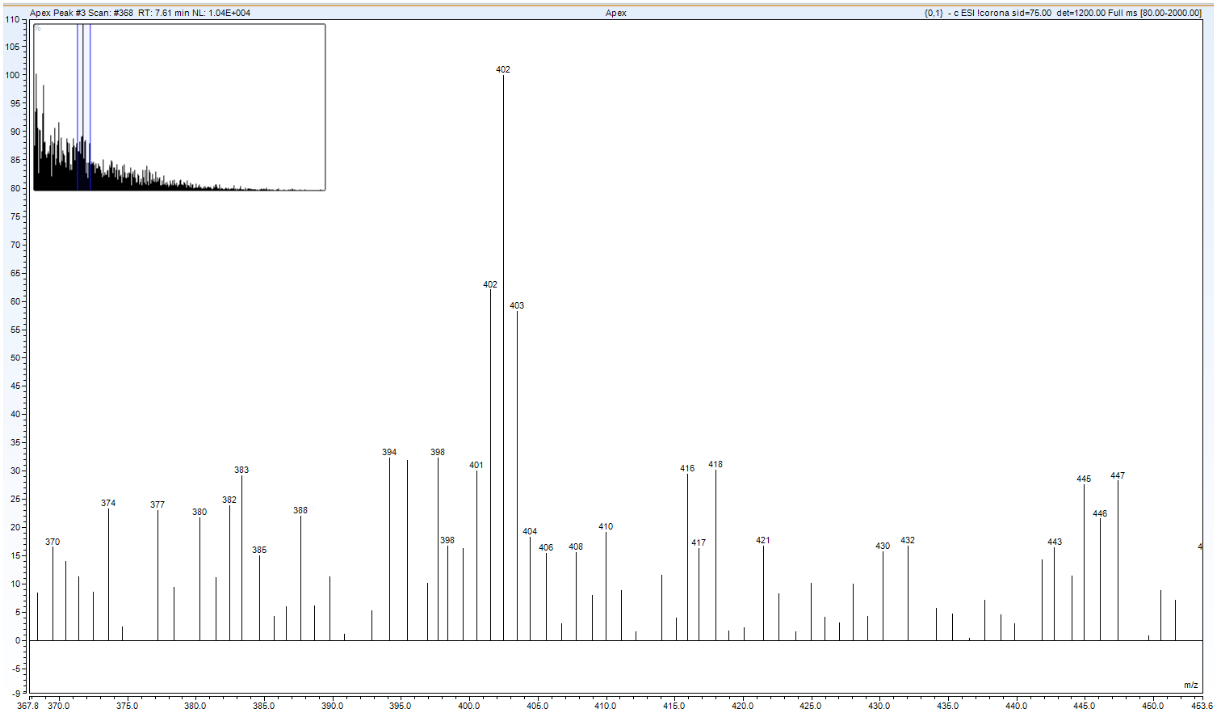

Figure S81. MS(-) of compound **13b**, retention time: 7.6 min, side product = *nido*-carborane derivative of **13b**.

Compound 14a

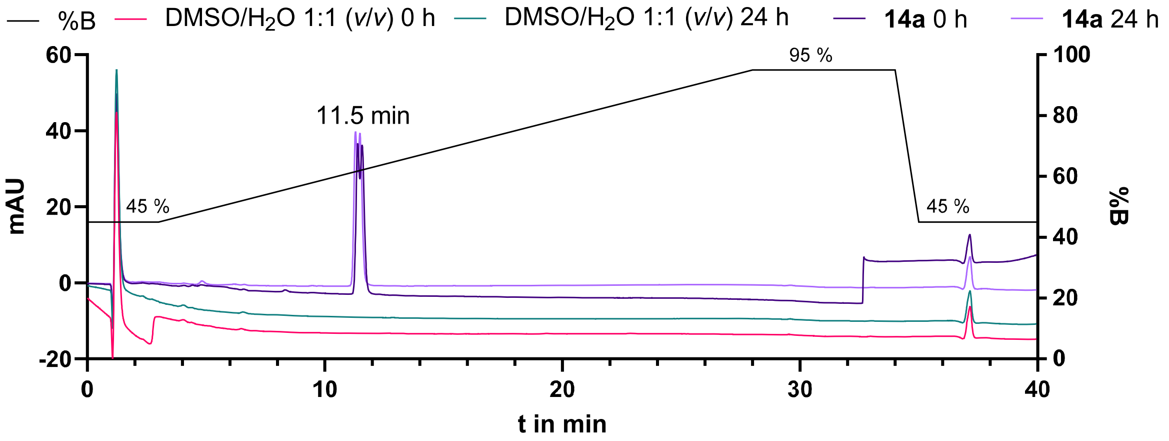

Figure S82. RP-HPLC chromatograms of blank (DMSO/H_2O, red and green) and compound **14a**, retention time: 11.5 min, purity after 1 d: ~95%.

Compound 14b

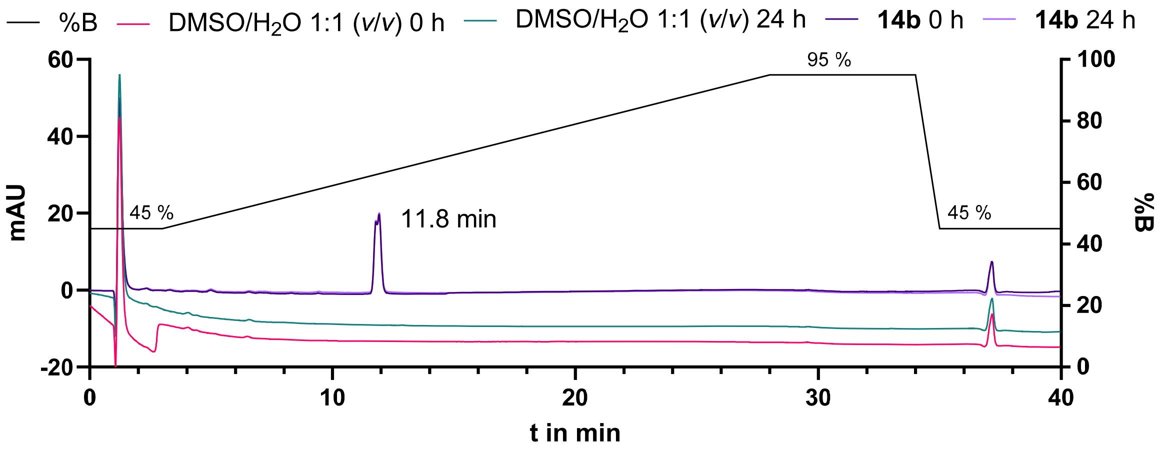

Figure S83. RP-HPLC chromatograms of blank (DMSO/H_2O, red and green) and compound **14b**, retention time:11.8 min, purity after 1 d: ~95%.

5 X-ray Crystallography Data of Compound 9

The data were collected on a Gemini diffractometer (Rigaku Oxford Diffraction) using Mo-Kα radiation and ω-scan mode. Data reduction was performed with CrysAlisPro[1] including the program SCALE3 ABSPACK for empirical absorption correction. The structures were solved by dual space methods with SHELXT[2] and the refinement was performed with SHELXL[3]. All non-hydrogen atoms were refined with anisotropic displacement parameters. For the room temperature measurement (**9-rt**), all hydrogen atoms (except CH and BH of the carborane units) are calculated on idealized positions, whereas for the low temperature measurement (**9**) all hydrogen atoms were located on difference Fourier maps calculated at the final stage of the structure refinement. Carborane carbon atoms could be localized with a bond length and displacement parameter analysis. Structure figures were generated with DIAMOND-4.[4] CCDC 2421113 (**9-rt**) and 2420756 (**9**) contains the supplementary crystallographic data for this paper. The data can be obtained free of charge via https://summary.ccdc.cam.ac.uk/structure-summary-form (or from the Cambridge Crystallographic Data Centre, 12 Union Road, Cambridge CB2 1EZ, UK; fax: (+44)1223-336-033; or deposit@ccdc.cam.uk).

[1] Rigaku Oxford Diffraction, (1995-2024), CrysAlisPro Software system, Rigaku Corporation, Wroclaw, Poland.
[2] SHELXT: G. M. Sheldrick, Acta Cryst. A71 (2015) 3–8.
[3] SHELXL: G. M. Sheldrick, Acta Cryst. C71 (2015) 3–8.
[4] DIAMOND 4: K. Brandenburg, Crystal Impact GbR, Bonn, Germany.

Table S1: Fundamental structure parameters

Compound	**9-rt**	**9**
Empirical formula	$C_{27}H_{46}B_{20}F_3N_3O_2S$	$C_{27}H_{46}B_{20}F_3N_3O_2S$
Formula weight	749.93	749.93
Temperature [K]	298(2)	130(2)
Wavelength [pm]	71.073	71.073
Crystal system	Triclinic	Triclinic
Space group	$P\overline{1}$	$P\overline{1}$
Unit cell dimensions		
a [pm]	1075.96(4)	1061.47(5)
b [pm]	1214.89(4)	1201.65(6)
c [pm]	1655.01(6)	1646.14(8)
α [deg]	98.599(3)	97.339(4)
β [deg]	104.221(3)	104.553(4)
γ [deg]	93.242(3)	94.157(4)
Volume [nm^3]	2.0637(1)	2.0037(2)
Z	2	2
$\rho_{(calculated)}$ [Mg/m^3]	1.207	1.243
μ [mm^{-1}]	0.124	0.128
F(000)	776	776
Crystal size [mm^3]	0.47 · 0.17 · 0.03	0.28 · 0.16 · 0.03
Θ_{Min} / Θ_{Max} [deg]	2.303 / 28.317	2.302 / 27.930
Index ranges	-13 ≤ h ≤ 14<br>-16 ≤ k ≤ 14<br>-21 ≤ l ≤ 21	-13 ≤ h ≤ 13<br>-15 ≤ k ≤ 15<br>-21 ≤ l ≤ 20
Reflections collected	28777	28070
Indep. reflections (R_{int})	8924 (0.0497)	8547 (0.0760)
Completeness (Θ) [deg]	99.9 % (25.35)	99.9 % (25.35)
T_{Max} / T_{Min}	1.00000 / 0.97588	1.00000 / 0.98246
Restraints / parameters	49 / 626	0 / 689
Gof on F^2	1.009	1.015
R1 / wR2 (I>2σ(I))	0.0626 / 0.1223	0.0616 / 0.1225
R1 / wR2 (all data)	0.1490 / 0.1562	0.1221 / 0.1512
Residual electron density [e·$Å^{-3}$]	0.187 / -0.263	0.269 / -0.382
Comments	†[1]	-
CCDC No	2421113	2420756

†[1]: The carborane atoms C6 and B11 are disordered with a ratio 0.62(3):0.38(3). The CF_3 substituent (C27, F1 to F3) is disordered as well with a ratio 0.81(1):0.19(1).

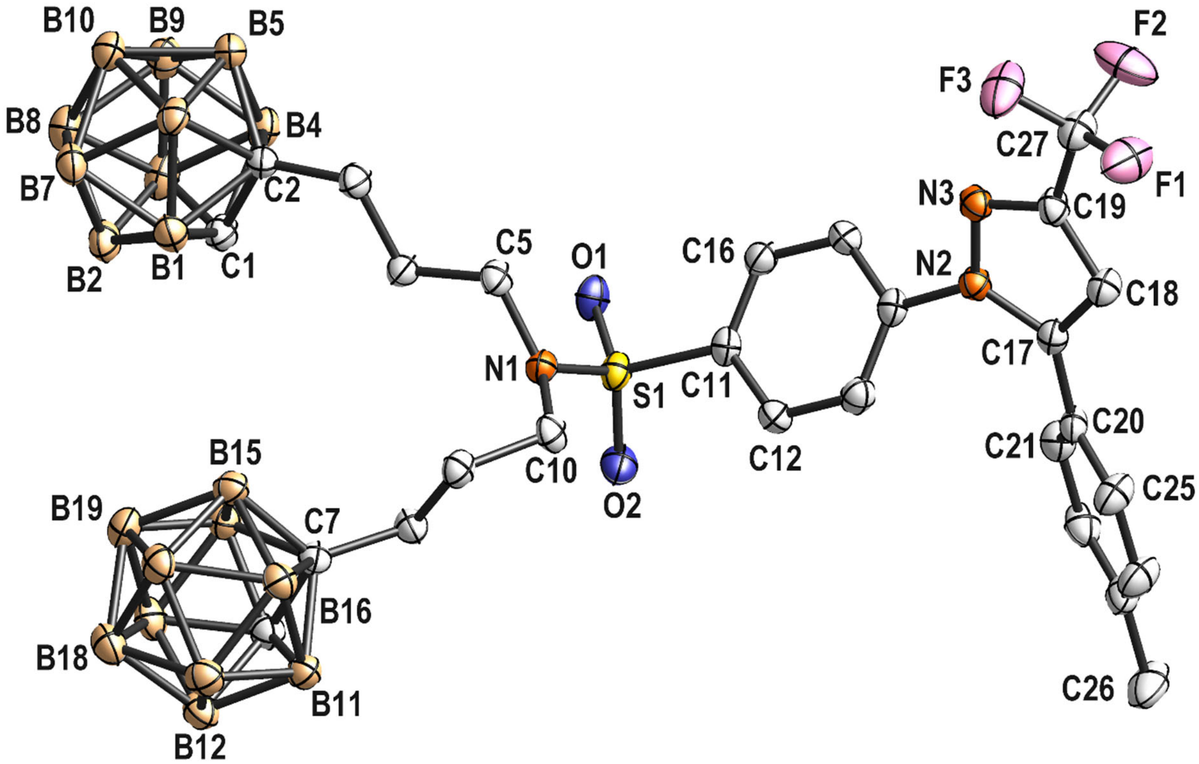

Figure S84. Molecular structure and labeling scheme of **9**. Hydrogen atoms were omitted for clarity. Displacement ellipsoids are drawn at the 50% probability level.

6 COX Inhibition Assay

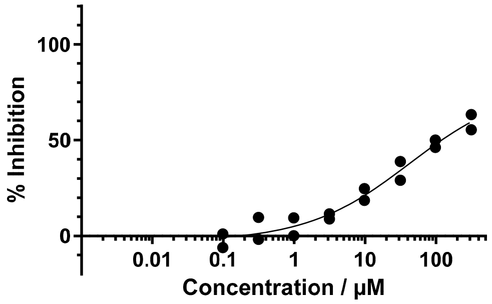

Figure S85. Inhibition of COX-2 by **6** in the concentration range between 0.1–320 µM.

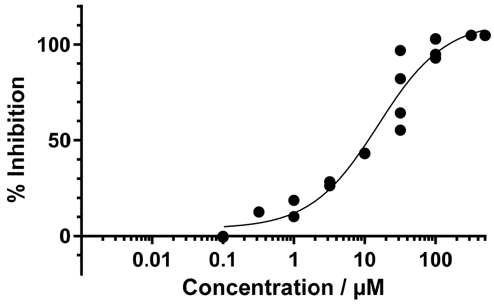

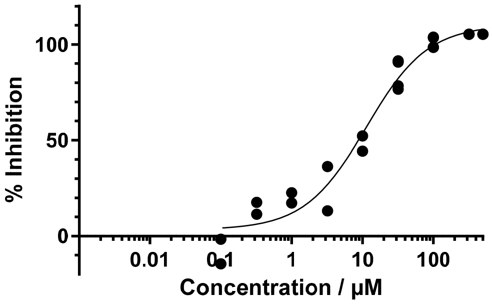

Figure S86. Inhibition of COX-1 (left) and COX-2 (right) by **7** in the concentration range between 0.1–500 µM.

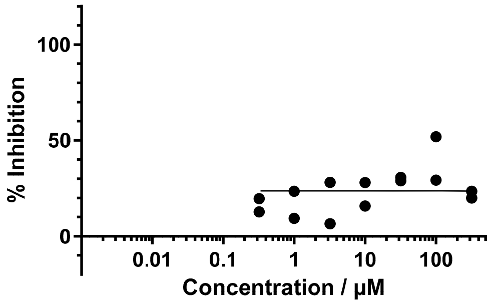

Figure S87. Inhibition of COX-1 by **9** in the concentration range between 0.32–320 µM.

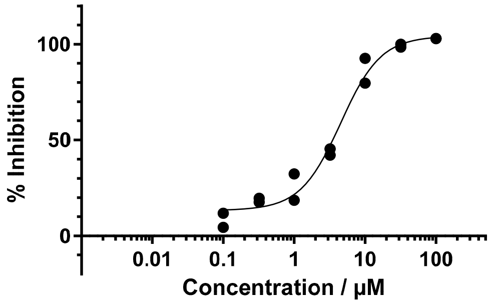

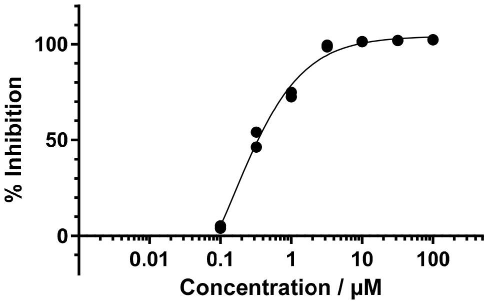

Figure S88. Inhibition of COX-1 (left) and COX-2 (right) by **10** in the concentration range between 0.1–100 µM.

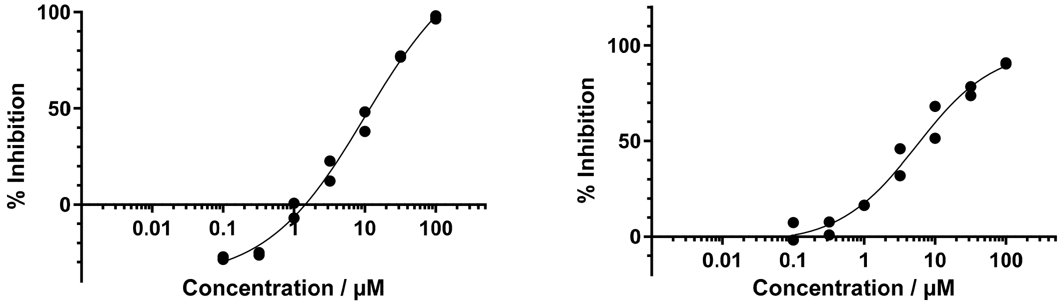

Figure S89. Inhibition of COX-1 (left) and COX-2 (right) by **14a** in the concentration range between 0.1–100 μM.

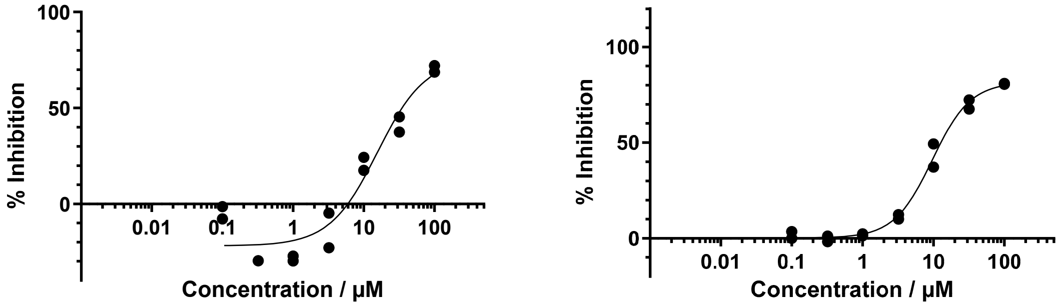

Figure S90. Inhibition of COX-1 (left) and COX-2 (right) by **14b** in the concentration range between 0.1–100 μM.
